# Astrocytes as Guardians of Neuronal Excitability: Mechanisms Underlying Epileptogenesis

**DOI:** 10.3389/fneur.2020.591690

**Published:** 2020-11-26

**Authors:** Quirijn P. Verhoog, Linda Holtman, Eleonora Aronica, Erwin A. van Vliet

**Affiliations:** ^1^Leiden Academic Center for Drug Research, Leiden University, Leiden, Netherlands; ^2^Department of Neuropathology, Amsterdam Neuroscience, Amsterdam UMC, University of Amsterdam, Amsterdam, Netherlands; ^3^Stichting Epilepsie Instellingen Nederland (SEIN), Heemstede, Netherlands; ^4^Center for Neuroscience, Swammerdam Institute for Life Sciences, University of Amsterdam, Amsterdam, Netherlands

**Keywords:** glia, astrogliosis, seizures, epilepsy, treatment, gliotransmission, blood-brain barrier, neuroinflammation

## Abstract

Astrocytes are key homeostatic regulators in the central nervous system and play important roles in physiology. After brain damage caused by e.g., status epilepticus, traumatic brain injury, or stroke, astrocytes may adopt a reactive phenotype. This process of reactive astrogliosis is important to restore brain homeostasis. However, persistent reactive astrogliosis can be detrimental for the brain and contributes to the development of epilepsy. In this review, we will focus on physiological functions of astrocytes in the normal brain as well as pathophysiological functions in the epileptogenic brain, with a focus on acquired epilepsy. We will discuss the role of astrocyte-related processes in epileptogenesis, including reactive astrogliosis, disturbances in energy supply and metabolism, gliotransmission, and extracellular ion concentrations, as well as blood-brain barrier dysfunction and dysregulation of blood flow. Since dysfunction of astrocytes can contribute to epilepsy, we will also discuss their role as potential targets for new therapeutic strategies.

## Introduction

Epilepsy is a common neurological disease that is estimated to affect roughly 1–2% of the population (1). Despite the fact that quite some anti-epileptic drugs (AEDs) have been developed in the last decades, a large number of patients still fail to respond to these AEDs. This is associated with increased morbidity and mortality and since these patients need life-long care this is also an economic burden for society. Furthermore, patients feel stigmatized and report a reduced quality of life (2). Therefore, it is of crucial importance to find novel drug targets in order to develop novel therapeutic strategies. Moreover, disease-modifying therapies are currently not available and require a better understanding of the underlying disease processes. In the past two decades, astrocytes have been increasingly acknowledged as key players in the etiology and pathogenesis of epilepsy. Therefore, astrocytes should be considered as promising targets for new therapeutic strategies.

The human brain is comprised of ~100 billion cells, classically divided into neurons and glial cells, although new types of brain cells are still being discovered up to date (3, 4). Glia cells in the central nervous system are typically classified into four cell types: (1) astrocytes, (2) microglia, (3) oligodendrocytes, and (4) their progenitors, neuron-glial antigen 2(NG2)-glia (5). For almost a century it was believed that glial cells outnumbered neurons 10:1 (6). However, it has been shown that the actual ratio of glial cells compared to neurons is closer to 1:1 and may in fact be lower than 1 (6, 7). Nevertheless, the remarkably conserved numerical relationship between glia and neurons over 90 million years of evolution supports the notion that glial cells are crucial for normal brain functioning (8). These numbers suggest a far more prominent role for astrocytes in the brain than long considered. In addition to its vast number, it is estimated that a single astrocyte touches and interacts with up to 2 million synapses with its processes (9).

Although all four glial cell types play a pivotal role in normal brain function, in this review we will focus on astrocytes which are key homeostatic regulators in the central nervous system and play important roles in the pathophysiology of epilepsy (10).

For many years, astrocytes were regarded as “glue” that bound neuronal elements together, providing mere structural support for the brain. In fact, astrocytes are playing a pivotal role in brain homeostasis. From recent transcriptome studies it became clear that different subtypes of astrocytes exist that are not only anatomically and spatiotemporally restricted, but also show varying degrees of heterogeneity of morphology and physiology in distinct brain regions (11, 12). The relevance of astrocytic heterogeneity is also evident in its distinct subpopulations and cortical layer-specific gene signatures that underline the comprehensive involvement of astrocytes in physiology (13–15). More sophisticated research strategies paired with a systemic evaluation and comparison of different glial markers will lead to a better understanding of the role of astrocytes in the central nervous system under physiological and pathophysiological conditions.

**Figure 1:**
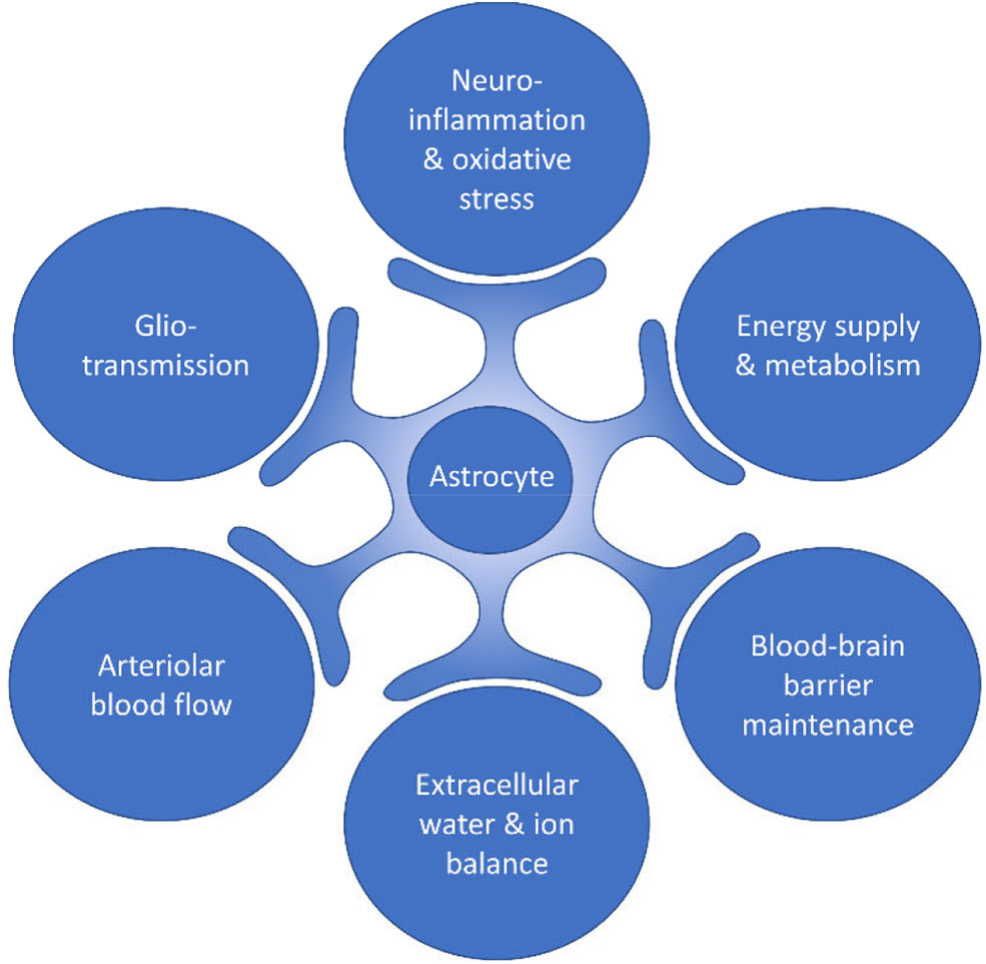
Processes within the brain in which astrocytes are involved. Astrocytes have been shown to be involved in important processes such as neuroinflammation and oxidative stress, energy supply and metabolism, blood-brain barrier maintenance, extracellular water and ion balance, arteriolar blood flow, and gliotransmission.

Astrocytes have been shown to be involved in important processes such as brain inflammation (16, 17) and oxidative stress (18), energy supply and metabolism (19–21), support of synaptic function and plasticity (22, 23), extracellular balance of neurotransmitters (24, 25), extracellular water and ion homeostasis (26, 27), blood-brain barrier (BBB) maintenance (28, 29), and regulation of blood flow [(30, 31); Figure 1].

**Figure 2:**
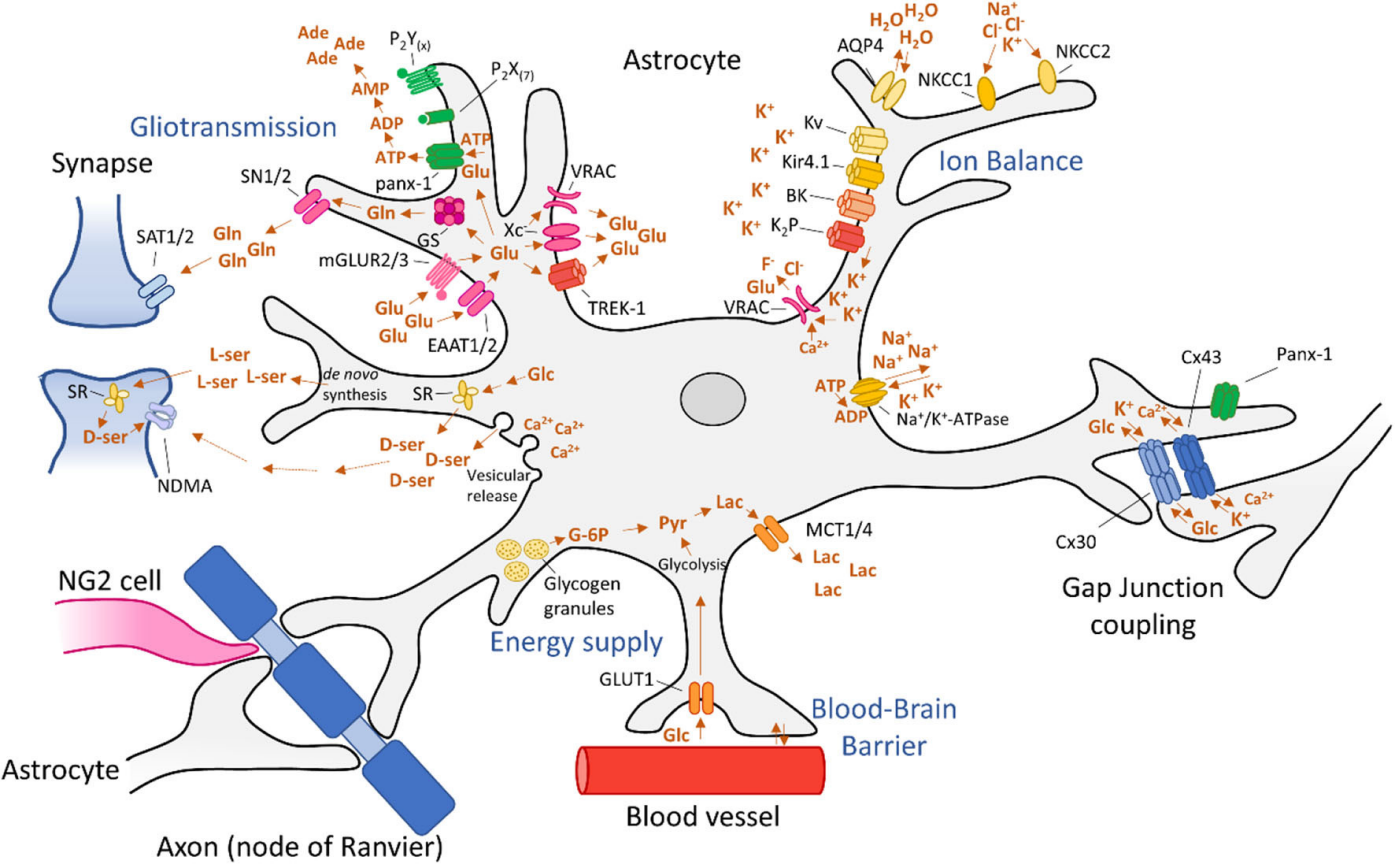
Astrocytic processes involved in epileptogenesis. Dysregulation of astrocyte functions can lead to epileptogenesis via disturbed energy metabolism and gliotransmission, alterations in extracellular ion concentrations, as well as dysfunction of the blood-brain barrier and dysregulation of blood flow. These mechanisms are discussed in detail in the main text.

Although astrocytes employ many processes that protect the brain from hyperexcitability, dysregulation of glial functions may cause hyperexcitability or promote the development of epilepsy by a multitude of mechanisms. In the following paragraphs, we will focus on the underlying processes that can promote epileptogenesis, including astrogliosis, disturbed energy metabolism and gliotransmission, alterations in extracellular ion concentrations, as well as dysfunction of the BBB and dysregulation of blood flow (Figure 2).

## Astrogliosis

**Figure 3:**
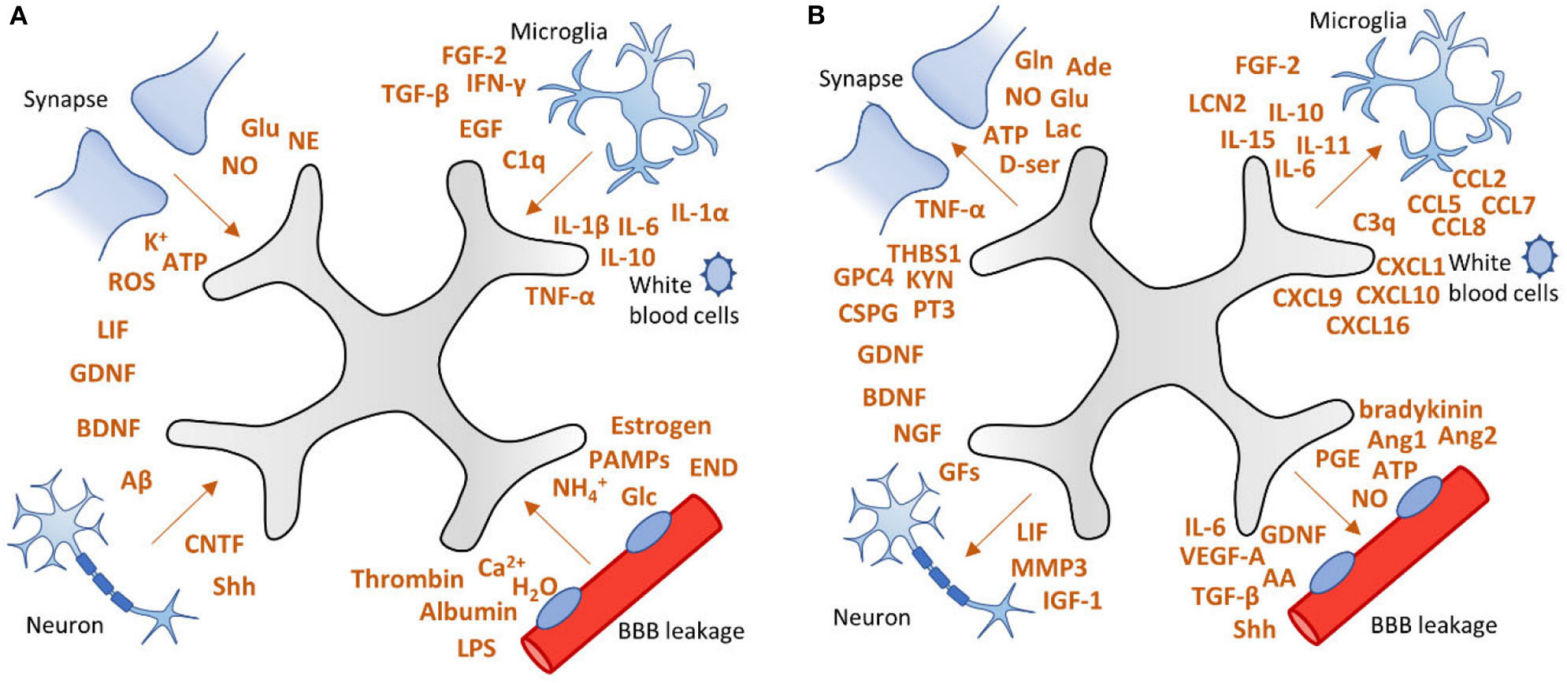
Factors involved in astrogliosis. **(A)** After brain injury, astrocytes can receive “instructions” from their environment and respond to a plethora of signaling molecules. **(B)** In turn, astrocytes send “instructions” to their environment by releasing a variety of factors, including pro-inflammatory cytokines, growth factors, neurotransmitters, as well as vascular mediators. This vicious cycle may lead to persistent activation of astrocytes which can contribute to epileptogenesis. Adapted from Sofroniew (32).

Due to brain injury induced by status epilepticus, stroke or traumatic brain injury, astrocytes receive “instructions” from their environment (Figure 3A) and in response to these molecular signals, the number of astrocytes increases and the astrocyte expression profile as well as its morphology, biochemistry and functionality changes, a process called reactive astrogliosis (32, 33). In turn, reactive astrocytes can send “instructions” to their environment (Figure 3B). The term reactive astrogliosis has been introduced in the nineteenth century to characterize morphological and behavioral changes within astrocytes upon pathophysiological conditions caused by various central nervous system diseases. In the beginning, efforts were focused on the morphological changes astrocytes experience during reactive astrogliosis, but over the past three decades a body of evidence has been collected that support astrogliosis heterogeneity and acknowledges a spectrum of molecular, cellular and functional changes within astrocytes upon reactive astrogliosis (32, 34).

The existence of spatiotemporal and anatomically localized subtypes of astrocytes needs to be taken into account when evaluating astrogliosis in the context of experimental epilepsy models, including the consequential effects on epileptogenesis and related neurobehavioral comorbidities, by employing genetic targeting studies and pharmacological therapies.

Cell-specific transcriptomics have revealed that astrocytes undergo massive changes in gene expression when they switch to a reactive phenotype (33). One of the most prominent changes during reactive astrogliosis is characterized by cell hypertrophy and upregulation of glial fibrillary acidic protein (GFAP), vimentin, nestin, and/or inducible nitric oxide synthase (iNOS) (35, 36). In addition, reactive astrocytes may produce and release a variety of factors, including pro-inflammatory cytokines (37), complement factors (38), gliotransmitters (39–41), reactive oxygen species (ROS) (42), trophic factors (43), and vascular endothelial growth factor (VEGF) [(44); Figure 3].

In particular, pro-inflammatory cytokines may affect astrocytes profoundly and cause changes that perpetuate astrogliosis and promote epileptogenesis (45, 46). Cytokines are widely studied in the context of reactive astrogliosis (47) and epilepsy (48). In this paragraph, we will focus on cytokines that exacerbate epilepsy progression and may therefore be interesting for therapeutic intervention. The most studied cytokines regarding astrogliosis and epilepsy are interleukin-1 beta (IL-1β), IL-6, and tumor necrosis factor-alpha (TNF-α); pro-inflammatory cytokines that can be released by reactive astrocytes (49, 50) and activated microglia (51). In a complex pathology such as epilepsy, more cytokines are playing roles in the alleviation and exacerbation of the disease. Figure 3 shows a fraction of cytokines involved in astrocytosis. For further reading into cytokine involvement in epilepsy the reader is directed to the following reviews (45, 46, 52).

Numerous studies have shown upregulation of IL-1β, IL-6, and TNF-α in animals with (recurrent) seizures (53–59) and patients with epilepsy (60–63). IL-1β can affect neurotransmitter receptors (64, 65), induce calcium influx by N-methyl-D-aspartate (NMDA) and 3-hydroxy-5-methyl-4-isoxazolepropionic acid (AMPA)-mediated mechanisms in neurons (66, 67), lead to alterations in expression of microRNAs in astrocytes (68–70), as well as potassium channels (71), metalloproteinases (72), altered astrocytic glutamate uptake (73, 74) and calcium uptake (75), and induces astrocytic release of other pro-inflammatory cytokines (50). IL-6 activates the Grp130/JAK/STAT pathway and thereby induces the release of additional pro-inflammatory cytokines, further endorsing inflammation (76). In addition, high levels of IL-6 lead to decreased astrocytic glutamate uptake via excitatory amino acid transporter 2 (EAAT2; formerly glutamate transporter 1) and even promote the release of glutamate by improving the activity of the astrocytic cystine/glutamate antiporter (Xc^−^) (76). Finally, IL-6 increases BBB permeability (77).

Similar to IL-6, TNF-α decreases astrocytic uptake of glutamate (78, 79) via purinergic signaling, thereby activating presynaptic NDMA receptors (80). Furthermore, TNF-α increases excitatory strength of AMPA receptors and at the same time decreases the amount of γ-aminobutyric acid (GABA)_A_ receptors in neurons, impairing inhibitory signaling (81). Finally, release of pro-inflammatory cytokines often stimulate additional release of cytokines (50), and it is suggested that this perpetual exacerbation of inflammatory signaling contributes to epilepsy (16, 82, 83). VX-765, a small molecule inhibitor of interleukin-converting enzyme (ICE)/caspase-1, thereby inhibiting biosynthesis of IL-1β, has been shown to reduce the number and duration of seizures in rats (84) and mice (85) and has even been tested in Phase II clinical trials (ClinicalTrials.gov Identifier: NCT01501383). In a Phase IIa randomized double blind placebo-controlled study in drug-resistant focal onset epilepsy, VX-765 had delayed beneficial effects (subjects with ≥50% reduction in seizure frequency) that persisted after drug discontinuation (86). Furthermore, the IL-1 receptor antagonist Kineret (anakinra) showed a drastic improvement of seizure control in patients with super-refractory status epilepticus secondary to febrile infection-related epilepsy syndrome (FIRES) (87–90), as well as in patients with drug-resistant epilepsy (91, 92).

To our knowledge, there is no therapy that directly targets IL-6 or its receptor IL-6R, but it has been shown that the antiepileptic drug valproate affects IL-6 serum levels, hinting at a possible mechanistic involvement of IL-6 (93).

Another example is Adalimumab, a TNF-α monoclonal antibody that has been tested in Rasmussen's encephalitis, leading to seizure improvement in a small cohort of patients (94). Furthermore, n-3 docosapentaenoic acid-derived protectin D1 is a pro-resolving mediator that was administered to epileptic mice, showing subsequent downregulation of IL-1β and TNF-α mRNA and consequently a 50% decrease of seizure activity and a 40% decrease in seizure duration (95). Finally, 1400 W, an inhibitor of inducible nitric oxide synthase (iNOS/NOS-II) showed promising results in a rat model of kainic acid-induced epilepsy, since it suppressed astrogliosis, microgliosis, neurodegeneration, mossy fiber sprouting, and had disease modifying effects (96).

Reactive astrogliosis is implicated in acquired and genetic types of epilepsy, including neurodevelopmental diseases [i.e., tuberous sclerosis complex; (17, 97–99)]. Changes of activity and gene expression of key proteins that are involved in epilepsy pathology such as glutamine synthetase (GS) (100), adenosine kinase (ADK) (101, 102), Aquaporins (AQPs) including AQP4 (103, 104), inward rectifying potassium (K_ir_) channels including K_ir_4.1 (105, 106), and monocarboxylate transporters (MCTs) (107, 108) have been observed in resected brain tissue of patients with temporal lobe epilepsy (TLE). Initially, the astrocytic response can be beneficial for the brain, promoting restoration of brain homeostasis. However, a vicious cycle may lead to persistent astrogliosis which can affect metabolic activity (109–111), ion buffering (112), gap junction (GJ) connectivity (113, 114), neurotransmitter uptake (115, 116), and promotes neuronal death, BBB dysfunction (44), and onset of seizures (117, 118). In the following chapters we will elaborate how these changes can affect neuronal excitability and contribute to epileptogenesis.

Besides affecting molecular pathways, astrocytes participate in bilateral signaling with microglia (119, 120). This interglial crosstalk has implications on both physiological and pathological processes (121, 122). Astrocytes influence microglial behavior by releasing molecules that regulate microglial functions. In turn, microglia are able to drive astrocytes from a neuroprotective to a neurotoxic phenotype (123), thereby potentially affecting the ability of astrocytes to protect against neuronal excitability. This bidirectional crosstalk may induce a persistent inflammatory environment under pathological conditions and may therefore exacerbate disease severity. Recent studies have shown that activated microglia induce neurotoxic phenotypes in astrocytes by secretion of pro-inflammatory mediators such as IL-1α, TNF-α, and complement component subunit 1q (C1q) (119, 124). Crosstalk between astrocytes and microglia may also be involved in epileptogenesis and should be taken into account when conducting studies into the mechanisms that drive epilepsy. Although attention has been primarily focused on astrocyte interactions with other central nervous system cell types, there is recent evidence that astrocyte functionality is influenced by the gut microbiome, and that this cross-talk between gut microbiota and brain, involving astrocytes, may have crucial implications in the development and progression of central nervous system disorders (125, 126). For instance, different types of gut bacteria may positively or negatively modulate the astrocytic inflammatory response (126–128).

## Energy Supply and Metabolism

Under physiological conditions, glucose is the primary metabolic substrate of the brain and is required to maintain the transmembrane potential of neurons (21). Glucose is transferred from the blood into the brain by glucose-transporters (GLUTs). Then, glucose is metabolized into glucose-6-phosphate (G-6P) by hexokinase [(129); Figure 2]. Subsequently, it can undergo two types of metabolization: glycolysis or metabolization by the pentose phosphate pathway. During glycolysis, pyruvate is formed, producing ATP. Pyruvate can then be oxidized in mitochondria by the tricarboxylic acid (TCA) cycle or by oxidative phosphorylation, producing 30–34 molecules of ATP at the cost of oxygen (129). In addition to neuronal energy supply, astrocytes are also equipped with a glucose-metabolism to meet the local energy demand. In fact, in times of hypoglycaemia and during periods of high neuronal activation, astrocytes take over the energy supply completely (130, 131). Astrocytes are able to process glucose by a mechanism similar to that of neurons. Astrocytes express glucose transporter 1 (GLUT1) allowing for glucose uptake (Figure 2). Glucose is then metabolized into G-6P by hexokinase (HK) and further into lactate, via pyruvate by an isoenzyme of lactate dehydrogenase (LDH) 5 (20). Subsequently, lactate is exported from astrocytes into the extracellular space by monocarboxylate transporters (MCTs) 1 and 4 (132) and taken up into neurons, which convert lactate into pyruvate. This alternative pathway constitutes the astrocyte-neuron lactate shuttle (21). Alternatively, G-6P is converted into G-1P by phosphoglucomutase (PGM) and then into uridine triphosphate (UDP)-glucose by UDP-glucose pyrophosphate (UDPGPP) (130). Finally, UDP-Glucose is converted into glycogen by glycogen synthase (GYS). Glycogen can then be stored in glycogen granules, usually clustered in places of great synaptic density (133). When required, glycogen can be metabolized back to G-6P via the same route in reverse or mediated by glycogen phosphatase (GP) (134).

### Glucose Sustains Synaptic Activity During Seizures

During seizures, excessive synaptic activity causes a rapid drop of glucose and a corresponding rise in lactate. Surrounding tissue responds to this by increasing glucose-administration to neurons by increasing blood perfusion and volume (135). At the same time, glucose can be distributed by astrocytes via by gap junctions (GJs) to reach distal neurons [(136); Figure 2]. During the excessive energy demands of seizures, astrocyte-derived lactate becomes an essential energy source for neurons (21). Furthermore, astrocytes can store glycogen which can supply energy to neurons via the lactate shuttle to sustain neuronal activity during seizures. Therefore, reducing brain glucose levels is considered anticonvulsive (137).

One way of achieving this is by the ketogenic diet, which is a low-carbohydrate and high-fat and adequate protein diet (138, 139). Thereby, the brain switches from a glucose-sustained metabolism to ketosis during which ketones, such as β-hydroxybutyrate, acetoacetate, and acetone are formed, which are thought to be important mediators for the suppression of seizures during the diet (140). In addition to the ketogenic diet, other antiepileptic diets have been proposed such as the modified Atkins diet, the medium-chain triglyceride ketogenic diet, and the low glycaemic index treatment (141, 142). Ketogenic diets are quite efficient in the alleviation of seizures in children, but also in adults with refractory epilepsy (138, 143, 144). However, it is difficult to adhere to the diet since it is not palatable. Furthermore, weight loss, constipation, high level of low-density lipoprotein, and elevated total cholesterol are most frequently reported as adverse effects (143, 145). Therefore, alternative approaches to inhibit glycolysis or interfere with lactate formation are studied. For instance, the use of the glycolysis inhibitor such as 2-deoxy-2-glucose has been proposed as a direct mechanism of lowering brain glucose, which has acute anticonvulsant and chronic antiepileptic actions in various epilepsy models (146–148). Furthermore, inhibition of LDH suppresses pilocarpine and kainic acid-induced seizures. Interestingly, LDH is also inhibited by the AED stiripentol (149). Another approach is to utilize GJ blockers that impair astrocytic intercellular glucose trafficking, thereby partially reducing the required energy for epileptiform activity (150). Taken together, these data imply that targeting specific brain glucose-pathways is an ambitious and challenging, but also a promising approach to interfere with epileptogenesis. Reducing glucose levels may be achieved by specific diets, local glycolysis-inhibition or by inhibition of GJs.

### Gap Junctions

Astrocytic GJs are comprised of two “hemichannels” which are made up of 6 subunits or connexins (Cx) (151). Astrocytes predominantly express connexins Cx43, but also Cx30, Cx26, Cx40, Cx45, and Cx46 (152, 153). One of the functional properties of GJs is to facilitate inter-astrocyte transportation of glucose and glucose-metabolites (150). In addition, GJs are able to propagate intercellular Ca^2+^ signaling through release of ATP (153, 154). Furthermore, GJs permit potassium transport between astrocytes, allowing K^+^ influx to redistribute to sites of lower concentration, supporting spatial K^+^ buffering (discussed in detail in a following paragraph). GJs reduce the threshold for seizures by facilitating spatial K^+^ buffering and glutamate transport. The involvement of GJs in spatial K^+^ buffering is reflected in the AQP4^−/−^ mouse model in which increased GJ coupling compensates for the loss of K^+^ uptake assisted by AQP4 (155). In line with this, mice with GJ-coupling deficiencies were shown to develop seizures and have problems with glutamate and K^+^ clearance (156).

Neuroprotective properties of GJs have been reported and therapeutically interfering with GJ functionality may introduce side effects (157). Moreover, uncoupling (loss of connectivity through loss of GJs) of astrocytic endfeet has been found to precede neurodegeneration and spontaneous seizure generation in a mouse model of TLE (158). Different expression patterns have been reported in studies on animal models and human tissue (159, 160). In astrocytes of sclerotic human hippocampal tissue, expression of connexins appears unchanged (161). It has been proposed that instead subcellular reorganization or post-translational modification of connexins accounts for the loss of GJ functionality in TLE. This could explain the variability in connexin expression in TLE found in earlier studies (159).

On the other hand, GJs may fuel synaptic and epileptiform activity by intracellular trafficking of metabolites to sustain neuronal activity at sites of high demand. GJs facilitate the spread of Ca^2+^ waves contributing to epileptogenesis by introducing a feedback loop from neurons to astrocytes (162). Furthermore, neuronal GJs are thought to be involved in the synchronous discharges during seizure activity (163, 164). These data suggest that inhibiting GJs has anticonvulsive effects.

Indeed, it has been shown that GJ blockers such as carbenoxolone (165–167), mefloquine (168), quinine (166, 167, 169), and quinidine (170) alleviate seizure severity in various animal models of epilepsy, although a general consensus on the exact mode of action and the efficacy of these compounds is still lacking. Anandamide and oleamide are fatty acids of the endocannabinoid family that have been demonstrated to inhibit intercellular GJs from glial cells (171, 172). Both anandamide (173, 174) and oleamide (175, 176) have been shown to have anticonvulsant effects *in vivo*, hinting at the involvement of glial networks in seizures.

## Gliotransmission

The concept of “gliotransmission” remains one of the most controversial topics in astrocyte biology. The term gliotransmitter is loosely defined as chemically active transmitters that origin from glial cells which may participate in or affect the excitatory or inhibitory network of neurons. Numerous studies have been performed showing a plethora of astrocytic released gliotransmitters: (1) amino acids including glutamate (177–179), D-serine (180, 181), GABA (182–184) and glycine (185–187), (2) nucleotides, such as adenosine 5′-triphosphate (ATP) (188–190), (3) organic acids including lactate (191–193), taurine (194, 195), and homocysteic acid (196, 197), and (4) peptides such as atrial natriuretic peptide (ANP) (198) and brain-derived neurotrophic factor (BDNF) (199, 200). Some argue that cytokines are in a way also gliotransmitters as they are chemically active too and may affect neuronal excitability, albeit mainly via indirect mechanisms. However, in this review we will further focus on the most studied gliotransmitters: glutamate, D-serine, and ATP and give an insight on how these gliotransmitters affect neuronal excitability.

### Ca^2+^-Dependent Gliotransmitter Release

In the early 1990s the first Ca^2+^ imaging studies were performed, showing increased astrocytic intracellular Ca^2+^ concentrations after local synaptic activity (201–203). A general consensus developed stating that astrocytes are in fact “excitable” cells and may respond to a wide range of neuronal factors and synaptically released spill-over neurotransmitters, and at the same time release so-called gliotransmitters that can communicate to neurons (24). In addition, newer imaging techniques showed that astrocytes appear to facilitate spontaneous focal Ca^2+^ oscillations or transients (204–206) and may even propagate Ca^2+^ signals to adjacent astrocytes (207–209). Unfortunately, due to limitations in experimental approaches required to understand the complexity of gliotransmission, it proved difficult to replicate findings in different models, or translate data from *in vitro* to *in vivo*. A heated debate followed in which contrasting evidence from various studies raised the question whether or not astrocytes contribute to information processing within the neural circuitry under physiological conditions (210). In addition, the dependence on Ca^2+^ signaling has been challenged time and again and is under critical review (211). To go further into this debate is beyond the scope of this review and the reader is directed to excellent literature on the topic (210, 212, 213).

Nowadays, a strong foundation of evidence that supports the bidirectional communication between neurons and astrocytes established the concept of a tripartite system that was originally proposed in the late 1990s (214). Progress on research neuron-glia crosstalk showed that the central role of astrocytes, besides regulation of brain homeostasis, is information processing. A body of evidence supports the existence of coordinated neuron-astrocyte network signaling, in which astrocytes are able to modulate neuronal excitability and synaptic transmission (206, 215–217). In turn, neuronal communication to astrocytes influences astrocytic signaling which may have implications in epilepsy (215, 218).

Two types of astrocyte “excitation” are well documented: neuron-dependent excitation and spontaneous excitation (24). There is evidence of Ca^2+^-dependent astrocytic release of different types of gliotransmitters including glutamate (204, 219–221), D-serine (222–224), and ATP (225, 226). To what extent these mechanisms are in fact dependent on Ca^2+^ or how they may or may not play a role in synaptic transmission under physiological conditions is discussed elsewhere (211, 227, 228).

### Ca^2+^-Independent Mechanisms of Gliotransmitter Release

In addition to Ca^2+^-dependent mechanisms of gliotransmitters, several Ca^2+^-independent mechanisms have been identified for some, but not all gliotransmitters. Astrocytes facilitate glutamate release by targeting the two-pore domain K^+^ channel (TREK-1) (220), through the pannexin-1 (panx-1) (229), and Cx43 hemichannels (230, 231), by volume-regulated anion channels (VRACs) (194), reversible glutamate transporters (232–234), and *in vitro* via the (Xc^−^) (235, 236) and the ionotropic P2X purinoceptor 7 (P_2_X7R) [(237); Figure 2]. Astrocytic ATP is released through GJ channels such as panx-1 (229, 238) and Cx43 hemichannels (231, 239), and in culture via mechanically-induced release of ATP by P_2_X7R (240, 241).

The relevance of these mechanisms is demonstrated by the changes that occur under pathophysiological circumstances such as in the epileptogenic brain (242–244), during astrogliosis (245), or upon swelling of astrocytes (246). Reactive astrocytes display increased expression and activation of hemichannels such as Cx43 (247) and panx-1 (248, 249), which is generally believed to result in increased gliotransmitter release (234). Moreover, during epilepsy, the opening probability of both astrocytic and neuronal hemichannels is increased, augmenting local excitotoxicity (250).

Using transgenic mouse models, ATP release through panx-1 channels has been shown to enhance neuronal excitability (251, 252). Furthermore, panx-1 in conjunction with P_2_X7R potentiates seizure activity in an animal model of epilepsy as well as in brain slices of patients with TLE (252). Panx-1 channels are exciting new targets as global panx-1 inhibition has anticonvulsive effects in animal models of epilepsy (168, 251).

Similar to panx-1, global inhibition of P_2_X7R reduces epilepsy severity after kainic acid-induced epilepsy (253), although additional *in vivo* data indicates that this is mainly due to neuronal effects (244, 254). Inhibiting P_2_X7R presumably affects astrocytes and other glial cells indirectly as well, by blocking P_2_X7R-mediated excitotoxic IL-1β release (255). P_2_X7R inhibitors such as Brilliant Blue G, A438079, AFC-5128, and JNJ-47965567 could attenuate chemically-induced kindling but did not possess remarkable effects in acute screening tests when administrated alone (256, 257).

Taken together, these data indicate that modulating astrocytic gliotransmitter release pathways may affect neuronal excitability. Interestingly, in a recent review several experimental pharmacological agents were highlighted as tools to control astrocyte signaling (258). These agents were tested in preclinical models, but some antiepileptic drugs may exert similar effects. This needs to be studied in more detail, as well as the use of these agents as novel therapeutic approaches. In the following paragraphs we will further elaborate on ways that gliotransmitters influence the pathophysiology of epilepsy.

### Glutamate

Astrocytes are able to influence extracellular concentrations of glutamate and an excess of extracellular glutamate is one of the mechanisms driving hyperexcitability (259, 260). Under physiological conditions, astrocytes restrict the diffusion of glutamate in the synaptic cleft and take up and recycle glutamate in a process called the glutamate/GABA-glutamine cycle [(25, 261); Figure 2]. In this cycle, glutamate is taken up by astrocytic glutamate-uptake channels such as the excitatory amino acid transporter 1 (EAAT1; formerly Na^+^-glutamate cotransporter) and EAAT2. Glutamate is then converted into glutamine by glutamine synthetase (GS) at the cost of ammonia and ATP. Interestingly, astrocyte subpopulations that express GS also co-express EAAT1 and EAAT2, emphasizing the link between the two mechanisms (262). After the conversion, glutamine is shuttled back to neurons through release by N system transporters (SN) 1 and 2 on the astrocytic membrane followed by neuronal uptake through system A transporters (SAT) 1 and 2 (263). It is then converted back into glutamate by neuronal glutaminase. In this cycle there are two steps by which astrocytes regulate glutamatergic excitability: (1) by removing excess glutamate from the extracellular space, and (2) by regulating the glutamine release from the astrocytic cytoplasm. In addition to glutamate uptake and conversion to glutamine, astrocytes are also able to synthesize glutamine *de novo*, by employing glycolytic enzymes and the TCA cycle, which produces glutamate from α-ketoglutarate and can then be converted to glutamine by GS (264).

Under pathophysiological circumstances, the regulation of the glutamate/GABA-glutamine cycle is perturbed, which can contribute to epileptogenesis. *In vivo* microdialysis experiments in the human brain showed that extracellular glutamate concentrations were chronically increased in the epileptogenic hippocampus compared to non-epileptic hippocampus (265, 266). This is likely the result of a failing glutamate uptake system from astrocytes in concert with a decreased ability to convert glutamate to glutamine.

### Glutamate-Uptake Channels

Downregulation of glutamate-uptake channels such as the EAAT1 (267–269) and EAAT2 (267, 269, 270) has been frequently reported in animal models of epilepsy. Furthermore, EAAT1 deletion causes prolonged seizure activity (271) and EAAT2 knockout mice exhibit spontaneous and recurrent seizures (272). In patients with TLE, EAAT1, and EAAT2 are also downregulated and this is colocalized with GFAP and the proliferation marker Ki-67, suggesting that this is dependent on astrogliosis (273, 274). Transcriptional reactivation of EAAT2 by a small molecule reduced the frequency of spontaneous seizures by 50% in a mouse model of tuberous sclerosis complex, postulating that restoring glutamate-uptake channels is seizure ameliorating (275). The loss of EAAT2 is not only evident on mRNA expression level, but the protein itself is also internalized and subsequently degraded (276). Therefore, preventing the degradation of EAAT2 may pose as an effective treatment for epilepsy as was recently shown in a mice model of kainic acid-induced epilepsy (277).

### Glutamine Synthetase

A growing body of evidence supports the notion that pathophysiological events such as epileptic seizures (278, 279) or astrogliosis (280, 281) result in a downregulation and corresponding decrease in immunoreactivity of GS (282). In accordance, chronic treatment with a GS-inhibitor caused spontaneous seizures in rats and increased local extracellular glutamate concentrations by 47%, showing that GS-deficiency alone is enough to evoke ictal events (260).

The exact mechanism of the lowered extracellular glutamate concentration due to GS-deficiency is still unknown, although several hypotheses have been proposed: (1) loss of GS leads to impaired clearance of glutamate because of a reduced conversion to glutamine, and (2) accumulating glutamate in astrocytes constitutes a concentration-dependent gradient that results in astrocytic glutamate release (282).

Indirectly, GS-deficiency may also contribute to hyperexcitability (282). Because glutamine is the precursor for the inhibitory neurotransmitter GABA, a reduction in astrocytic glutamine production evokes a local shortage of GABA. As the main inhibitory neurotransmitter of the brain, a local GABA shortage increases neuronal excitability and neuronal network synchronization (283). A second way that GS-deficiency affects local excitability is that a reduction in the glutamine metabolism consequently consumes less ammonia. Previously, it has been shown that high concentrations of local ammonia is neurotoxic and may even cause excitotoxicity by affecting chloride transport (284, 285). It is presently unclear to what extent these indirect mechanisms contribute to ictogenesis and research into this would certainly contribute to our understanding of how a GS-deficiency could cause epileptic seizures.

Evidence suggests that GS expression is dependent on neuronal survival as downregulation of GS in patients with TLE coincides with neuronal loss (100, 285). It has been proposed that neuronal loss precedes GS downregulation, and in turn, GS downregulation increases excitability (285). In addition, the resulting increase in extracellular glutamate may result in neuronal cell death (286), feeding a disease exacerbating cycle (285).

Other pathological changes within astrocytes such as swelling (194) and ischemia (233) may also affect glutamate release and extracellular concentrations. From all this data it becomes evident that the glutamatergic mechanisms that underlie excitability are exceedingly intricate and complex. Perturbations in any of the aforementioned glutamatergic mechanisms may lead to an increase of excitatory network activity, and eventually epilepsy (287).

### D-Serine

Presently, all mechanisms regarding D-serine release from astrocytes appear to be either directly linked to intracellular Ca^2+^ concentration (i.e., vesicular release), or are receptor activation-dependent, which is indirectly linked to local Ca^2+^ concentration (288). Amongst these are the adenosine type 2A receptor (A_2A_R) (289), bradykinin-type2 (B_2_) receptor (290), ephrinB3 receptor (291), ionotropic (292), and metabotropic (223) glutamate receptors, transforming growth factor (TGF)-β receptor (293), as well as muscarinic (294) and nicotinic (295) acetylcholine receptors. Despite its extensive regulation, it was recently proposed that astrocytic D-serine is not available in sufficient amounts to modulate synaptic activity *in vivo*, under physiological conditions (296). Instead, astrocytic *de novo* synthesized L-serine that is required for the production of D-serine in neurons may affect synaptic activity after conversion to D-serine by neuronal serine racemase (SR) (297, 298). It is noteworthy however, that the profound effects of pathophysiological conditions such as epileptic seizures or astrogliosis dramatically change the behavior and expression profiles of astrocytes, which may in turn affect the dynamics of D-serine production. In culture (224) and in brain slices (222), astrocytes are able to express SR, and most notably, in an animal model of traumatic brain injury, it was shown that the switch from neuronal SR to astrocytic SR was in part responsible for traumatic brain injury-induced synaptic damage (299). Furthermore, increased release of D-serine may contribute directly to neuronal excitotoxicity by acting upon the NMDA receptor as a co-agonist together with glutamate (Figure 2). Indeed, it has been shown that lowering D-serine in epileptic rats by administering a competitive SR-inhibitor resulted in reduced seizure duration and severity, dependent on ERK signaling (300). These data suggest that modulating D-serine production could pose as a strategy for epilepsy treatment.

### ATP

The actions of ATP and its metabolite adenosine arguably extend even further than that of glutamate or D-serine by acting upon purinergic receptors, influencing astrocytes, neurons, microglia, oligodendrocytes, and blood vessels (301). The complexity of ATP-mediated effects is demonstrated by studies that report both excitatory and inhibitory consequences from astrocytic ATP release. For instance, it has been shown that ATP activates the astrocytic ionotropic P_2_X and metabotropic P_2_Y receptors resulting in increased GABA release (302). Furthermore, ATP released by astrocytes may induce action potentials on inhibitory interneurons, thereby decreasing the excitatory network output (303). In contrast, astrocytic ATP negatively regulates GABAergic inhibitory transmission on post-synaptic neurons (226), suggesting that astrocytic ATP release may augment ictogenesis. Moreover, it has been shown that astrocytic ATP activates neuronal P_2_X receptors leading to pro-epileptic effects (304), including enhanced pre-synaptic release of glutamate (305). As argued earlier, purinergic signaling through P_2_X receptors is mediated by ATP release through panx-1 channels. However, it appears there is a clear distinction between astrocytic and neuronal panx-1, and surprisingly, astrocytic panx-1 may even be seizure alleviating (306) [for review see (234, 307)]. It is hypothesized that worsening of seizure activity in mice deficient of astrocytic panx-1 is likely connected to increased ADK levels in astrocytes.

### Adenosine Kinase

ADK is a key metabolic enzyme of astrocytes that catalyses the conversion of adenosine into adenosine monophosphate. Therefore, modulation of ADK expression is of interest in the context of epilepsy. Adenosine is a potent anticonvulsant and is released during seizures (17). It is a substrate for the adenosine receptor family of which the A_1A_R and A_2A_R are the most studied. Anti-epileptic effects are mainly mediated by A_1A_R signaling which activates K_ir_ channels and inhibit Ca^2+^ channels, but also exert astrocyte-function modulating effects by stimulatory coupling to K^+^ and Cl^−^ ion channels (308–310). Since neuronal excitability is modulated by activation of A_1A_, A_2A_, A_2B_, and A_3_ receptors, the equilibrium of intra- and extracellular adenosine critically affects epilepsy severity (311).

Synaptic adenosine is mainly regulated by ADK, because uptake of adenosine into astrocytes is quickly equilibrated by nucleoside transporters (ENTs) (310). Upon brain injury, ADK is transiently downregulated for ca. 2 h, recovering to baseline levels over the course of 24 h (101). This acute response to stress results in increased adenosine levels, enhancing protective effects against brain injury, including status epilepticus and traumatic brain injury, through increased activation of A_1A_R (310). However, elevated synaptic adenosine levels also activate the A_2A_R, which signaling may in turn desensitize and downregulate the A_1A_R (312, 313). Indeed, it has been shown that in epileptogenic circuits, stimulation of A_2A_R downregulates A_1A_R (314, 315). Recently, it has been shown that a 3-fold induction of A_2A_R is present in astrocytes within the hippocampus of patients with TLE (316). Increased A_2A_R signaling promotes astrogliosis by various mechanisms including by increased stimulation of glutamate release, synaptic actions of BDNF in the hippocampus and through the Akt/NF-κB pathway (317–320). The shift in A_1A_R/A_2A_R signaling also causes a change from inhibition to promotion of cell proliferation and may contribute to the development of proliferative scar-forming astrocytes (310). Moreover, after the initial downregulation of ADK, its expression increased in reactive astrocytes (101). This is also confirmed in experimental animal models of epilepsy and human TLE brain slices (102). ADK inhibitors have since been developed (321–323) and tested in animal models of epilepsy (324, 325). Unfortunately, the first line of ADK inhibitors showed liver toxicity side effects, but recently efforts have been made to develop novel ADK inhibitors which may present a viable therapeutic strategy for epilepsy in the future (326).

## Water and Ion Transport

One of the functions of astrocytes is to maintain homeostatic extracellular water and ion balance in the brain. Changes in ion or water balance affect local synaptic activity by modifying the concentration gradient upon which the electrochemical potential is based. Ultimately, this may result in hyperexcitability by mechanisms discussed below. To ensure homeostatic ion balance is preserved, astrocytes express a plethora of passive, ATP-, voltage-, and volume-gated ion channels (Figure 2 and Table 1).

**Table 1:** Selection of ion and water transporters associated with homeostatic astrocyte function and epileptiform activity in disease.

**Ion**	**Transporter**	**Alteration**	**Expression in temporal lobe epilepsy**
H_2_O	AQP1 AQP4 AQP9 EAAT1	Causing astrogliosis (327) Mislocalization (328)	↑ (329) ↑ Overall (330, 331) ↓ Perivascular (331, 332) ↓ (329) No change (100) ↓ Hippocampus (274)
K^+^	BK K_2_P K_ir_4.1 K_ir_5.1 K_ir_2.1 K_v_ Na^+^/K^+^-ATPase NKCC1	Transient upregulation (333, 334)	↓ Mossy fibers (335) ↑ CA1 ↓ Dentate gyrus (336) ↓ Hippocampus, Perivascular (106) No change (337) ↑ CA1, CA3, Dentate gyrus (338) ↑ Hippocampus (339) ↓ (Suggested) (340) ↑ Subiculum, hippocampus (341)
Na^+^	EAAT1 EAAT2 Na^+^/K^+^-ATPase NCX NKCC1 TRPA1 TRPCs TRPV1	Transient upregulation (333, 334)	No change (100), ↓ Hippocampus (274) No change (100), ↓ Hippocampus (274) ↓ (Suggested) (340) ↓ Dentate gyrus (342) ↑ Subiculum, hippocampus (341) ↑ (343) ↑ (344–346) ↑ Cortex, Hippocampus (347)
Ca^2+^	AMPA NMDA NCX P_2_X7 PMCA TRPA1 TRPCs TRPV1	Different splice variant (348) Subcellular relocation Transient upregulation	↑ Hippocampus (349) ↑ Dentate gyrus (350) ↓ Dentate gyrus (342) No change (253) ↑ Dentate gyrus (342) ↑ (343) ↑ (344–346) ↑ Cortex, Hippocampus (347)
Cl^−^	NKCC1	Transient upregulation (333, 334)	↑ Subiculum, hippocampus (341)

*↑: upregulation, ↓: downregulation*.

Intra- to extracellular water balance is of significance for epilepsy because it directly correlates to local osmolarity that plays a role in excitability. Astrocytes are particularly sensitive to changes in extracellular osmolarity (351). For instance, as a result of traumatic brain injury, up to ~30% of patients develop hyponatremia. This causes a decrease in osmolarity, after which astrocytes swell considerably (352, 353). As astrocytes swell up by water uptake, the extracellular space volume decreases. In turn, astrocytes respond by activating VRACs that work to restore the concentration equilibrium by expelling osmolytes and anions such as F^−^ and Cl^−^ [(351, 354, 355); Figure 2]. These mechanisms are of interest, because the volume of the extracellular space affects synaptic activity (26). In addition, opening of VRACs is accompanied with substantial amounts of glutamate (356, 357). These VRACs open primarily in astrocytes with high concentrations of K^+^ or during hypo-osmolar conditions that often occur during ictal events, although it has been proposed that Ca^2+^ signaling may induce swelling and thereby open VRACs as well (39, 354). Due to lack of selectivity and inability to differentiate between astrocytic and neuronal channels, modulation of VRACs has not been tested in animal models of epilepsy, but may pose an interesting avenue for seizure treatment by potentially lowering extracellular glutamate levels. Care should be taken when following this approach as a tight regulation of osmolarity and the volume of the extracellular space is required for homeostatic brain function.

### Spatial Potassium Buffering

The most critical ion flux governed by astrocytes in relation to epilepsy is that of potassium. In a process called spatial potassium buffering, astrocytes clear the extracellular space of excess K^+^ during neuronal repolarization. To ensure rapid uptake of K^+^ ions, astrocytes express different types of K^+^-channels, including K_ir_ channels, Ca^2+^-sensitive potassium (BK) channels, voltage-gated potassium (K_v_) channels, two-pore domain (K_2_P) channels and several co-transporters [(358); Figure 2 and Table 1]. Upon entering astrocytes, K^+^ is dispersed to areas of lower potassium concentration and travels intercellularly to adjacent astrocytes by GJs. The spatial buffering model is based on the fact that the low resting potential of astrocytes provide a driving force for K^+^ uptake in regions of high neuronal activity.

Perturbations in the astrocytic K^+^ buffering is therefore directly responsible for increased neuronal activity and excitability. In addition, high extracellular K^+^ concentrations may affect the activity of ion and water transporters such as AQP4, EAAT2, Na^+^/Ca^2+^-exchanger (NCX), sodium-potassium pump Na^+^/K^+^-ATPase, and Na^+^/K^+^/Cl^−^-cotransporter (NKCC) (355). Furthermore, increases in the extracellular K^+^ concentration induce opening of panx-1 channels, may cause seizure activity by release of ATP and glutamate (252, 307, 355).

### Aquaporins

The integral membrane protein AQP4 is responsible for most of the water uptake by astrocytes (Figure 2), but a total of 13 human AQPs (0–12) have been characterized (26, 359). Of those, expression and protein levels of AQP1, 3, 4, 5, 8, 9, and 11 have been shown in rodent brains (329). Aside from aquaporins, there are additional mechanisms to transport water, for instance via co-transporters such as EAAT1 (360).

It is hypothesized that concomitant water uptake by AQP4 during spatial K^+^ buffering decreases the volume of the extracellular space, thereby inducing an increase of [K^+^]_0_, which in turn stimulates astrocytic K^+^ uptake (358, 361). In epileptic foci, elevated extracellular potassium concentrations due to impaired K^+^ uptake by astrocytes may cause hyperexcitability (following paragraphs) (362). Surprisingly, AQP4 expression is increased in the hippocampus of patients with TLE (329, 330). However, local expression of the protein at perivascular astrocytic endfeet is lost. This is due to downregulation of the dystrophin gene that encodes for the protein responsible for anchoring AQP4, which causes the AQP4 channel to be mislocalized (363, 364). Decrease of perivascular AQP4 channels has been shown to perturbate spatial potassium buffering (103, 365). *In vivo* models of acute epilepsy with AQP4^−/−^ mice showed elevated seizure thresholds, which can be explained by the increase in extracellular space volume from impaired water uptake (366). In addition, prolonged seizure activity was measured, likely due to impaired K^+^ uptake (159, 331). Recently, it was shown that loss of perivascular AQP4 precedes seizure onset after kainic acid-induced epilepsy in rats, suggesting an involvement in epilepsy etiology (367). Taken together, these data suggest a that dysregulation of AQP4 plays an important role in epilepsy pathology.

Expression of other members of the AQP family such as AQP1 and AQP9 is also frequently reported to be altered in animal models of seizures (368), epilepsy (369), and traumatic brain injury (370, 371). In addition, expression changes in resected brain tissue of patients with TLE have been reported. Transcriptome and ELISA analysis showed that AQP1 expression is increased and AQP9 is decreased in human hippocampal sclerotic tissue compared to adjacent neocortex tissue (329). Moreover, AQP1 and AQP4 have been shown to play a role in cell growth and migration, and may be involved in glial scar formation (327, 372). Overexpression of AQP1 may therefore exacerbate disease progression by worsening astrogliosis. AQP9 is an aquaglyceroporin, meaning it is also able to shuttle glycerol, urea, and monocarboxylates such as lactate, suggesting that loss of AQP9 may disrupt local lactate levels which could affect neuronal excitability (327). Evidently, the functionality of aquaporins extends far beyond their primary function of channeling water molecules and it is important that the mechanisms behind these proteins are elucidated, to help understand their impact on epilepsy pathophysiology.

### Inward Rectifying Potassium Channels

Under physiological conditions, the main inward rectifying potassium channel K_ir_4.1 is abundantly expressed in cortical astrocytes, as well as in the hippocampus and thalamus (373, 374). In addition, heteromeric channels of K_ir_4.1/5.1 are expressed in astrocytes of the forebrain. Furthermore, expression of several members of the K_ir_2 and K_ir_6 families have been reported in astrocytes (375, 376).

K_ir_4.1 has been shown to colocalize with AQP4, suggesting a functional role for water transport in relation to K^+^ buffering (104). *In vitro* experiments have shown that K_ir_4.1 is able to directly bind to α-syntrophin, a member of the dystrophin-complex that has been shown to assist in AQP4 localization (377, 378). However, expression and immunoreactivity of K_ir_4.1 is not altered in AQP4^−/−^ mice, nor is AQP4 immunoreactivity altered in K_ir_4.1^−/−^ mice, suggesting that functionality of neither transporter is fully dependent on the other (332, 379). Nevertheless, clearance of extracellular K^+^ by K_ir_4.1 is partially dependent on simultaneous water flux by AQP4, to enable proper osmolarity for K^+^ distribution and uptake. Recently, the synergy between AQP4 and K_ir_4.1 channel mediated K^+^ uptake has been validated by a mathematical model of neuroexcitation (380). Furthermore, in an experiment where heterologous AQP4 and K_ir_4.1/5.1 were co-expressed in *Xenopus* oocytes, cell shrinkage produced K^+^ currents, indicating another, more direct functional coupling between AQP4 and K_ir_ channels (381).

During astrogliosis, proliferative astrocytes are shifted toward an immature phenotype in which they lose K_ir_4.1 and EAAT1 functionality, reducing spatial K^+^ buffering and impairing glutamate uptake (382, 383). Accordingly, K_ir_ is often reported to be downregulated in animal models of epilepsy (71, 384). More specifically, loss of K_ir_ immunoreactivity is located on astrocytic processes within epileptic foci, but not on astrocytes of the surrounding tissue (385). Furthermore, a significant loss of K_ir_4.1 immunoreactivity has been reported in resected hippocampal tissue of TLE patients (106). Interestingly, the loss of K_ir_4.1 was associated with loss of AQP4-associated proteins α-syntrophin and dystrophin, further emphasizing the link between K_ir_4.1, dystrophin-complex, and AQP4 localization. Decrease or loss of K_ir_4.1 or K_ir_4.1/5.1 channels undoubtedly cause perturbations in spatial K^+^ buffering, but functional mechanisms modulating epileptogenesis remain unidentified. Recently, it was shown that antagonism of K_ir_4.1 or suppression of K_ir_4.1 expression by siRNAs induces synthesis of BDNF (386). Expression of BDNF is upregulated in several animal models of epilepsy and in human epileptic disorders (387, 388). One way BDNF is proposed to promote seizures is by reduction of inhibitory synaptic transmission of GABA_A_ receptor signaling (389). In addition, release of BDNF has been shown to downregulate expression of K^+^/Cl^−^-cotransporter (KCC2) (390, 391). Knock-out of KCC2 has been shown to induce hyperexcitability in mice (392). This shows that impaired K_ir_4.1 signaling may result in hyperexcitability by a multitude of mechanisms.

### Other Potassium Channels

BK channels are expressed in astrocytic endfeet and they regulate vasodilation and vasoconstriction (Figure 2). BK channels are sensitive to calcium levels, membrane potential, and certain types of arachidonic acid (AA) metabolites which can lead to vasodilation or vasoconstriction (discussed in one of the following paragraphs) (358, 393). This is an important astrocytic property that supports the dynamic neuronal energy demand. In addition, calcium-dependent (BK_Ca_) channels regulate K^+^ export from astrocytes, directly affecting local excitability (394).

Under physiological conditions, BK channels participate in the spatial K^+^ buffering that is required for normal brain function. A specific subunit of the protein (β_4_) prevents the channel to contribute to neuronal membrane repolarization, which allows the channel to protect the brain from hyperexcitability (395). A gain-of-function mutation or a loss of β_4_ subunit activity removes the protective function and is associated with epileptic seizures (396, 397). In animal models of epilepsy, the β_4_ subunit is downregulated and a switch is made in subtypes of the BK channels, resulting in faster gating (335, 398). BK blockers may reverse the adverse effects of β_4_ subunit loss and have been able to reduce action potential firing in brain slices of epileptic rats (399) and reduce seizure activity *in vivo* in mice (400). Recently, efforts were made to develop BK blockers, that selectively target BK channels with a gain-of-function mutation in the β_4_ subunit, posing as a new strategy for therapy aimed at patients with retractable epilepsy (397).

Specific roles for other K^+^ channels such as K_v_ or K_2_P channels (Figure 2) remain elusive. Downregulation of K_v_ channels in astrocytes upon seizure activity has been reported and agonists of K_v_ have been suggested as anticonvulsant therapies, but additional research is required to understand how K_v_ channels are involved in excitability (339, 401). K_2_P channels are open at rest and thereby aid K_ir_ channels in driving the membrane potential of astrocytes to the K^+^ equilibrium, a feature thought to promote glutamate uptake (358, 402).

### Sodium Channels

Although astrocytes are considered non-excitable cells (in the classical sense that they are unable to produce action potentials), they dynamically express all 9 isoforms of Na_v_ sodium channels, with Na_v_1.5 as the main voltage-gated sodium channel (VGSC) (403). Expression of VGSCs is increased upon brain insult and during astrogliosis and appear to assist via a mechanism involving NCX (403–405). Little is known about the exact function of sodium channels in astrocytes, but it is believed that continuous Na^+^ influx is required to maintain [Na^+^]_i_ for activity of Na^+^/K^+^-ATPase [(406); Figure 2]. Na^+^/K^+^-ATPase assists in extracellular K^+^ buffering by uptake of K^+^ while simultaneously releasing Na^+^ at the cost of ATP. In fact, Na^+^/K^+^-ATPase, rather than K_ir_4.1, is responsible for most of the net uptake of K^+^. Changes in the activity of Na^+^/K^+^-ATPase have been proposed as an underlying mechanism for epilepsy (340, 407). Mutations in the gene encoding Na^+^/K^+^-ATPase were shown to cause seizure activity in animals (408) and patients with epilepsy (409).

Another mechanism supporting spatial K^+^ buffering is through the Na^+^/K^+^/Cl^−^ co-transporter (NKCC1; Figure 2), which has been shown to play a role in astrocytic swelling under conditions of high extracellular K^+^ (410–412). This is another example stressing the importance of the volume regulation of the extracellular space. Na^+^-transport can also be facilitated by members of the transient receptor potential (TRP) family, including “ankyrin” TRPA1, “canonical” TRPC1, TRPC4, TRPC5, and “vanilloid” TRPV4 receptors (413, 414). In addition to VGSCs and ion cotransporters, Na^+^ is transported over the membrane in conjunction with various other mechanisms such as glutamate uptake by EAAT1 (415), glutamine export by Na^+^/H^+^-coupled neutral amino acid transporters (SN1) and SN2 (416), and lactate shuttling by Na^+^/K^+^-ATPase (417).

### Calcium Transporters

Many astrocytes functions occur in response to focal or global Ca^2+^ transients. Therefore, a tight regulation of intra- and extracellular levels of Ca^2+^ is vital for homeostatic astrocytic functionality. Ca^2+^ can permeate the membrane through a variety of channels, including plasmalemmal Ca^2+^-ATPase (PMCA), TRPA1, TRPC1, TRPC4, TRPC5, TRPV1, ionotropic glutamate receptors AMPA and NMDA, purinergic receptors (i.e., P_2_X7) and by several ion exchangers of which the NCX is the most relevant [(153); Table 1]. Of note, astrocytes express all 3 isoforms of NCX and it has been shown that NCX colocalizes with Na^+^/K^+^-ATPase and glutamate receptors (418).

One mechanism in which focal Ca^2+^ transients in astrocytes regulate brain homeostasis is mediated by TRPA1. Influx of Ca^2+^ by TRPA1 regulates GABAergic transmission via the astrocytic GABA3 transporter (419) and D-serine release (420).

TRPCs are involved in store-operated Ca^2+^ entry and have been shown to contribute to Ca^2+^-mediated glutamate release in astrocytes (413). On the other hand, glutamate can activate astrocytic NMDA receptors and thereby induce Ca^2+^ influx, although they are ~2 times less permeable than their neuronal counterparts (421, 422).

### Chloride Transporters

Anions are also transported across astrocytic membranes. Astrocytes express different isoforms of potassium-chloride and cation-chloride cotransporters of the solute carrier 12 (SLC12) gene family, which include NKCC1, Na^+^/Cl^−^-cotransporter (NCC) and KCC1, KCC3, KCC4 (423–425). Mounting evidence suggests KCC2 is neuron-specific, but some experimental data shows that KCC2 may be present in astrocytes (424, 425). The main role of KCCs in astrocytes is volume regulation, whereas in neurons they regulate membrane potential by keeping intracellular Cl^−^ levels low, to enable GABAergic transmission (425, 426).

Astrogliosis causes a downregulation of KCC2 and NKCC1 in cortical pyramidal neurons, thereby preventing the Cl^−^ gradient required for GABAergic transmission (287). In contrast, increased expression of NKCC1 has been found in hippocampal sclerotic tissue of patients with TLE (427). During the development of neurons, the ratio between KCC2 and NKCC1 changes, as KCC2 is upregulated and NKCC1 is downregulated in mature neurons (428). Considering that astrocytes may express both KCC2 and NKCC1, and at the same time appear to differentiate to an immature state during astrogliosis, it is plausible that this change in expression is also reversed in astrocytes in the sclerotic hippocampus. The shift in expression of KCC2 and NKCC1 has been shown in the subiculum of TLE patients, but is yet to be confirmed in astrocytes specifically (341).

Antagonism of NKCC1 reduces seizure frequency in patients with TLE (429). Interestingly, inhibition of NKCC1 with the diuretic bumetanide does not influence K^+^ buffering post-stimulation (430). In this study it was found that neither K_ir_4.1 nor NKCC1 inhibition changed K^+^ buffering after neuronal activation, but that Na^+^/K^+^-ATPase was mostly responsible for the post-stimulation K^+^ uptake. Nevertheless, the development of selective NKCC1 inhibitors may prove rewarding in the clinic (431).

## Blood-Brain Barrier Dysfunction

**Figure 4:**
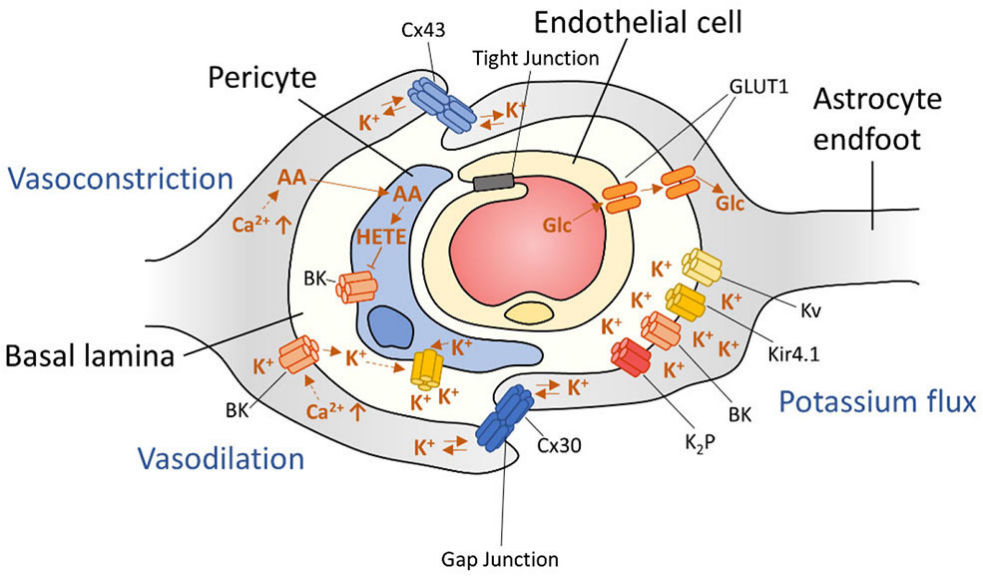
Schematic representation of arteriolar regulation at the neurovascular unit. Astrocyte signaling is able to influence neuronal vascularity by inducing both vasoconstriction and vasodilation through mechanisms that involve BK channels.

The BBB functions as a physical barrier to protect the brain from toxins, undesirable metabolites and ions that could permeate the brain from the blood stream. The BBB is comprised of endothelial cells that are connected via tight junctions [(28); Figure 4]. This physical barrier is considered the “first line of defence” for the brain. Astrocytes ensheath with their endfeet the endothelial cells (Figure 2) and serve as a “second line of defence.” Together with neurons, other glia cells and mural cells, they form the neurovascular unit. The main function of astrocytes at the BBB is the control of nutrient exchange with the bloodstream and maintaining BBB integrity (432).

Endothelial cells at the BBB express several transporter proteins and channels such as GLUT1, several amino acid carriers including EAAT1, EAAT2, EAAT3, and L-system for large neutral amino acids (LAT1), specific transporters (i.e., for nucleosides, nucleobases), non-specific transporters, such as multidrug transporters (MDTs), and organic ion transporters (28, 433). Importantly, astrocytes are able to alter the expression or activity of endothelial transporters including GLUT1 (434, 435) and MDTs such as P-glycoprotein (P-gp) (436, 437). Moreover, astrocytes may affect BBB permeability directly by changing the density of tight junctions (438, 439), for instance through release of angiopoietin 1 and 2, ATP, endothelin-1, fibroblast growth factor, glial cell line-derived neurotrophic factor (GDNF), glutamate, retinoic acid, nitric oxide or VEGF (440, 441).

Astrogliosis and neuroinflammation can lead to BBB dysfunction. Under inflammatory conditions, bradykinin is released in the blood, increasing BBB permeability by acting on endothelial (B_2_) receptors (442). In addition, bradykinin induces IL-6 release from astrocytes through activation of nuclear factor kappa-light-chain-enhancer of activated B cells (NF-κB) (443) resulting in modulation of endothelial tight junctions (77). Moreover, following brain injury astrocyte-mediated inflammation causes transient opening of the BBB (444). BBB dysfunction is common in epilepsy and can contribute to the development and progression of epilepsy (365, 445–448). In the following paragraphs we will discuss several mechanisms by which BBB dysfunction contributes to epilepsy pathophysiology.

### VEGF Signaling

Downregulation or loss of the proteins that make up tight junctions, such as zonula occludens (ZO-1), occludin, and claudins results in opening of the BBB. Loss of tight junctions is shown to be caused by increased expression of the VEGF receptor 1 and 2 in a rat model of pilocarpine-induced epilepsy (449). In addition, in an animal model of kainic acid-induced epilepsy it was shown that upregulation of VEGF-R1 and VEGF-R2 caused downregulation of ZO-1 (450). Furthermore, astrocyte-released VEGF has been shown to downregulate tight junction proteins claudin-5 and occludin (44). Several studies report increased VEGF release and receptor expression in patients suffering from refractory TLE (449, 451). The primary role of VEGF is to induce angiogenesis, which is correlated with seizure frequency (449). Angiogenesis and down-regulation of ZO-1 could be reversed by neutralization of VEGF, suggesting that VEGF signaling is involved in BBB dysfunction. This was demonstrated by oral administration of the VEGF pathway inhibitor sunitinib, which prevented seizures and epilepsy development in pilocarpine-induced seizures in rats, showing the potential of anti-angiogenesis therapies.

### Albumin Leakage

In epilepsy, angiogenesis is spatially correlated to leakage of serum proteins into the brain parenchyma (452). Under pathophysiological conditions, BBB leakage exposes the brain to plasma proteins such as IgG and albumin (445, 449, 453). Subsequently, astrocytes are able to internalize serum albumin by binding to the TGF-β receptors, inducing epileptogenesis via a mechanism similar to TGF-β_1_ induced TGF-β signaling (453). Importantly, TGF-β_1_ was upregulated during gliosis in periods after SE (454). Furthermore, TGF-β_1_ has been shown to downregulate K_ir_2.3 in reactive astrocytes (455). In turn, albumin-induced TGF-β signaling causes impaired GJ coupling and down-regulates K_ir_4.1 (453). This shows that TGF-β signaling interferes with potassium buffering in at least two distinct mechanisms involving inward rectifying channels. In addition, albumin extravasation into the brain has been shown to (1) transiently affect GJ coupling (456), (2) induce GFAP expression (457), (3) upregulate pro-inflammatory cytokine IL-6 (458), (4) reduce astrocyte potassium and glutamate clearance (459), and (5) induce excitatory, but not inhibitory synaptogenesis, contributing to potential hyperexcitability (460). Together, these data show that BBB dysfunction can promote epileptogenesis.

### Multidrug Transporters

BBB opening is associated with increased expression of MDTs (448, 461). Several studies have reported upregulation of genes encoding for MDTs, including P-gp (MDR1 gene), multidrug resistance protein (MRP) 1, MRP2, MRP5, and breast cancer resistance protein (BRCP) in the epileptogenic brain (448, 462–465). Overexpression of MDTs is shown in endothelial cells, but also in astrocytic endfeet and neurons. These transporters have been shown to affect drug transport in the brain and it has been proposed that drug resistance in patients with refractory epilepsy may be due to changes in MDTs (466–468). For instance, increased expression of P-gp causes enhanced efflux of AEDs, impairing AED entry into the brain. Indeed, P-gp blockers can increase AED levels in the brain and overcome pharmacoresistance in animal models of epilepsy (469–471), suggesting co-administration of antiepileptic drugs and a P-gp blocker may prove useful in patients as well. In contrast, recent experiments based on measurements of extracellular fluid unbound drug concentrations and mathematical models predicting drug target site concentrations, suggest that P-gp expression does not translate to BBB permeability for all AEDs, as other factors may affect target-site concentration more profoundly, such as brain tissue binding (472). Moreover, it is unclear whether changes in MDT expression are different between various animal models or correlate to different types of disease progression. Patient data confirms these speculations as it appears that therapeutic success appears to be influenced by the heterogeneity of the etiology of the seizures (473, 474). On the other hand, mounting evidence from patients that were co-administered with AEDs and P-gp blockers (i.e., Verapamil) show improved clinical outcome compared to AED only treatment (475–478).

### Arteriolar Blood Flow Regulation

Astrocytes also aid in the local regulation of vasoconstriction and vasodilation. Changes in intracellular Ca^2+^ at astrocytic endfeet can induce two major arteriolar regulating pathways: (1) The cytoplasmic phospholipase A2 (PLA_2_) pathway and (2) BK channel mediated vascular control [(393, 479); Figure 4]. Increased PLA_2_ activity results in production of AA, which is metabolized into various vasoactive compounds and is also able to pass the cell membrane to pericytes. Inside pericytes, AA is then metabolized into the vasoconstrictive 20-hydroxyeicosa-tetraenoic acid (20-HETE).

Different concentrations of Ca^2+^ in astrocytic endfeet are also able to regulate arteriolar dilation or vasoconstriction by induction of BK-channels that release K^+^ in the perivascular space—a space formed by the envelopment of astrocytic processes around arterioles (31, 393). In addition, BK channels respond to components of the PLA_2_ pathway, such as 20-hydroxyeicosatetraenoic acid (20-HETE), epoxy-eicosatetraenoic acids (EET)s, and prostaglandin E2 (PGE_2_). Importantly, these mechanisms are not mutually exclusive, and even overlap. Efforts of blocking either pathway individually did not result in total impairment of vascular control, emphasizing the extent of vascular control for homeostatic brain function (393). Together, these mechanisms can regulate cerebral blood-flow in the brain.

During epileptic seizures cerebral blood-flow and also cerebral blood volume are transiently increased as a response to the high energy demand of neurons (135). However, these increases are not sufficient to meet metabolic demands of synchronously activated neurons during ictal events. Several studies have found impaired neurovascular coupling in epilepsy (135, 480, 481). In some studies, this is correlated to hypoxia-induced tissue damage. Others suggest the possibility to predict ictal events based on increased cerebral perfusion preceding seizure onset in the clinic (482). Vast Ca^2+^ waves at astrocytic endfeet recorded during ictal events have been shown to regulate local arteriole responses, and these effects could be blocked by pharmacological inhibition of the Ca^2+^ signals in astrocytic processes (483). Although the underlying mechanisms behind the regulation of cerebral microcirculation in epilepsy are poorly defined, these data emphasize how astrocytes may control the neuronal micro environment during seizures.

## Concluding Remarks

Under physiological conditions, astrocytes protect neurons from becoming hyperexcitable. However, under pathophysiological conditions found before and during epilepsy, the evident and complex involvement of astrocytes in the neuronal network is perturbed. In this review we showed how aberrant astrocytic signaling and changes in astrocyte function contribute to the development and aggravation of epilepsy.

Despite an abundance of clues in the vast literature on the mechanistic involvement of astrocytes in epilepsy, there are presently no drugs in the clinic that target these mechanisms. In the near future it is imperative that we continue the development of drugs that specifically target mechanisms that are underlying the etiology of epilepsy and also focus on astrocytes as novel therapeutic targets. So far, pioneering preclinical studies have shown promising results.

Interestingly, recently it was suggested that astrocytes may also be used as biomarkers for epileptogenesis (484, 485). In this review the recently published evidence was reported, supporting the utility of measuring astrocyte activation, the soluble molecules they release, and the associated cognitive deficits during epileptogenesis for early stratification of animals developing epilepsy. Whether this may also be of clinical use needs to be investigated.

## Author Contributions

QV and LH developed the concept and prepared the first draft. EV and EA provided feedback and contributed to the writing of the manuscript together with QV and LH. All authors read, revised, and approved the final manuscript.

## Conflict of Interest

The authors declare that the research was conducted in the absence of any commercial or financial relationships that could be construed as a potential conflict of interest.

## References

[1] Hesdorffer DC Rochester A Katri N Cascino G Hauser W. Estimating risk for developing epilepsy. Neurology. (2010) 76:23–7. 10.1212/WNL.0b013e318204a36a 21205691 PMC3032191

[2] Baulac M De Boer H Elger C Glynn M Kälviäinen R Little A. Epilepsy priorities in Europe: a report of the ILAE-IBE epilepsy advocacy Europe task force. Epilepsia. (2015) 56:1687–95. 10.1111/epi.13201 26415919 PMC5019256

[3] Kimelberg HK Nedergaard M. Functions of astrocytes and their potential as therapeutic targets. Am Soc Exp Neurother. (2010) 7:338–53. 10.1016/j.nurt.2010.07.006 20880499 PMC2982258

[4] Boldog E Bakken TE Hodge RD Novotny M Aevermann BD Baka J. Transcriptomic and morphophysiological evidence for a specialized human cortical GABAergic cell type. Nat Neurosci. (2018) 21:1185–95. 10.1038/s41593-018-0205-2 30150662 PMC6130849

[5] Jäkel S Dimou L. Glial cells and their function in the adult brain : a journey through the history of their ablation. Front Cell Neurosci. (2017) 11:24. 10.3389/fncel.2017.00024 28243193 PMC5303749

[6] Bahney J Herculano-Houzel S Biology C. The search for true numbers of neurons and glial cells in the human brain: a review of 150 years of cell counting. J Comp Neurol. (2017) 524:3865–95. 10.1002/cne.24040 27187682 PMC5063692

[7] Herculano-Houzel S Miller DJ Kaas J Biology C. How to count cells: the advantages and disadvantages of the isotropic fractionator compared with stereology. Cell Tissue Res. (2016) 360:29–42. 10.1007/s00441-015-2127-6 25740200 PMC4380666

[8] Herculano-Houzel S. The glia/neuron ratio : how it varies uniformly across brain structures and species and what that means for brain physiology and evolution. Glia. (2014) 62:1377–91. 10.1002/glia.22683 24807023

[9] Oberheim NA Takano T Han X He W Lin JHC Wang F Uniquely hominid features of adult human astrocytes. J Neurosci. (2009) 29:3276–87. 10.1523/JNEUROSCI.4707-08.2009 19279265 PMC2819812

[10] Robel S Sontheimer H. Glia as drivers of abnormal neuronal activity. Nat Neurosci. (2016) 19:28–33. 10.1038/nn.4184 26713746 PMC4966160

[11] Zeisel A Hochgerner H Lönnerberg P Johnsson A Memic F van der Zwan J. Molecular architecture of the mouse nervous system. Cell. (2018) 174:999–1014. 10.1016/j.cell.2018.06.021 30096314 PMC6086934

[12] Batiuk MY Martirosyan A Wahis J de Vin F Marneffe C Kusserow C. Identification of region-specific astrocyte subtypes at single cell resolution. Nat Commun. (2020) 11:1220. 10.1038/s41467-019-14198-8 32139688 PMC7058027

[13] John Lin CC Yu K Hatcher A Huang TW Lee HK Carlson J. Identification of diverse astrocyte populations and their malignant analogs. Nat Neurosci. (2017) 20:396–405. 10.1038/nn.4493 28166219 PMC5824716

[14] Lanjakornsiripan D Pior BJ Kawaguchi D Furutachi S Tahara T Katsuyama Y. Layer-specific morphological and molecular differences in neocortical astrocytes and their dependence on neuronal layers. Nat Commun. (2018) 9:1623. 10.1038/s41467-018-03940-3 29691400 PMC5915416

[15] Duran RCD Wang CY Zheng H Deneen B Wu JQ Brain region-specific gene signatures revealed by distinct astrocyte subpopulations unveil links to glioma and neurodegenerative diseases. eNeuro. (2019) 6:1–19. 10.1523/ENEURO.0288-18.2019 PMC6449165 30957015

[16] Vezzani A Masa L Baram TZ. The role of inflammation in epilepsy. Nat Rev Neurol. (2011) 7:31–40. 10.1038/nrneurol.2010.178 21135885 PMC3378051

[17] Devinsky O Vezzani A Najjar S De Lanerolle NC Rogawski MA. Glia and epilepsy: excitability and inflammation. Trends Neurosci. (2013) 36:174–84. 10.1016/j.tins.2012.11.008 23298414

[18] Chen Y Qin C Huang J Tang X Liu C Huang K. The role of astrocytes in oxidative stress of central nervous system: a mixed blessing. Cell Prolif. (2020) 53:1–13. 10.1111/cpr.12781 32035016 PMC7106951

[19] Pellerin L Bouzier-Sore A Aubert A Serres S Merle M Costalat R. Activity-dependent regulation of energy metabolism by astrocytes an update. Glia. (2007) 55:1251–62. 10.1002/glia.20528 17659524

[20] Falkowska A Gutowska I Goschorska M Nowacki P. Energy metabolism of the brain, including the cooperation between astrocytes and neurons, especially in the context of glycogen metabolism. Int J Mol Sci. (2015) 16:25959–81. 10.3390/ijms161125939 26528968 PMC4661798

[21] Boison D Steinhäuser C. Epilepsy and astrocyte energy metabolism. Glia. (2018) 66:1235–43. 10.1016/j.physbeh.2017.03.040 29044647 PMC5903956

[22] Brunel N Volterra A Pitta MDE. Astrocytes: orchestrating synaptic plasticity? Neuroscience. (2016) 323:43–61. 10.1016/j.neuroscience.2015.04.001 25862587

[23] Hussaini SMQ Jang MH. New roles for old glue : astrocyte function in synaptic plasticity and neurological disorders. Int Neurourol J. (2018) 22:106–14. 10.5213/inj.1836214.107 30396259 PMC6234728

[24] Volterra A Meldolesi J. Astrocytes, from brain glue to communication elements: the revolution continues. Nat Rev Neurosci. (2005) 6:626–40. 10.1038/nrn1722 16025096

[25] Bak LK Schousboe A Waagepetersen HS. The glutamate/GABA-glutamine cycle: aspects of transport, neurotransmitter homeostasis and ammonia transfer. J Neurochem. (2006) 98:641–53. 10.1111/j.1471-4159.2006.03913.x 16787421

[26] Haj-Yasein NN Jensen V Ostby I Omholt SW Voipio J Kaila K. Aquaporin-4 regulates extracellular space volume dynamics during high-frequency synaptic stimulation A gene deletion study in mouse hippocampus. Glia. (2012) 60:867–74. 10.1002/glia.22319 22419561

[27] Vasile F Dossi E Rouach N. Human astrocytes : structure and functions in the healthy brain. Brain Struct Funct. (2017) 222:2017–29. 10.1007/s00429-017-1383-5 28280934 PMC5504258

[28] Abbott NJ Rönnbäck L Hansson E. Astrocyte-endothelial interactions at the blood-brain barrier. Nat Rev Neurosci. (2006) 7:41–53. 10.1038/nrn1824 16371949

[29] Serlin Y Shelef I Knyazer B Friedman A Biology C Sciences B. Anatomy and physiology of the blood-brain barrier yonatan. Semin Cell Dev Biol. (2015) 38:2–6. 10.1016/j.semcdb.2015.01.002 25681530 PMC4397166

[30] Gordon GRJ Mulligan SJ Vicar BAMAC. Astrocyte control of the cerebrovasculature. Glia. (2007) 1221:1214–21. 10.1002/glia.20543 17659528

[31] Iadecola C Nedergaard M. Glial regulation of the cerebral microvasculature. Nat Neurosci. (2007) 10:1369–76. 10.1038/nn2003 17965657

[32] Sofroniew MV Astrogliosis. Cold Spring Harb Perspect Biol. (2015) 7:1–16. 10.1101/cshperspect.a020420 PMC4315924 25380660

[33] Escartin C Guillemaud O Carrillo-de Sauvage MA. Questions and (some) answers on reactive astrocytes. Glia. (2019) 67:2221–47. 10.1002/glia.23687 31429127

[34] Sofroniew MV Vinters HV. Astrocytes: biology and pathology. Acta Neuropathol. (2010) 119:7–35. 10.1007/s00401-009-0619-8 20012068 PMC2799634

[35] Kettenmann H Ransom BR Neuroglia. 3rd ed New York, NY: Oxford University Press (2013).

[36] Robel S. Astroglial scarring and seizures : a cell biological perspective on epilepsy. Neurosci. (2017) 23:152–68. 10.1177/1073858416645498 27118807

[37] Brambilla R Bracchi-Ricard V Hu W Frydel B Bramwell A Karmally S. Inhibition of astroglial nuclear factor kappaB reduces inflammation and improves functional recovery after spinal cord injury. J Exp Med. (2005) 202:145–56. 10.1084/jem.20041918 15998793 PMC2212896

[38] Lian H Yang L Cole A Sun L Chiang AC Fowler SW. NFκB-activated astroglial release of complement C3 compromises neuronal morphology and function associated with alzheimer's disease. Neuron. (2015) 85:101–15. 10.1016/j.neuron.2014.11.018 25533482 PMC4289109

[39] Takano T Kang J Jaiswal JK Simon SM Lin JH Yu Y. Receptor-mediated glutamate release from volume sensitive channels in astrocytes. Proc Natl Acad Sci USA. (2005) 102:16466–71. 10.1073/pnas.0506382102 16254051 PMC1283436

[40] Jo S Yarishkin O Hwang YJ Chun YE Park M Woo DH. GABA from reactive astrocytes impairs memory in mouse models of Alzheimer's disease. Nat Med. (2014) 20:886–96. 10.1038/nm.3639 24973918 PMC8385452

[41] Wu Z Guo Z Gearing M Chen G. Tonic inhibition in dentate gyrus impairs long-term potentiation and memory in an Alzheimer's disease model. Nat Commun. (2014) 5:4159. 10.1038/ncomms5159 24923909 PMC4159602

[42] Hamby ME Hewett JA Hewett SJ. TGF-β1 potentiates astrocytic nitric oxide production by expanding the population of astrocytes that express NOS-2. Glia. (2006) 54:566–77. 10.1002/glia.20411 16921522

[43] Chou SY Weng JY Lai HL Liao F Sun SH Tu PH. Expanded-polyglutamine huntingtin protein suppresses the secretion and production of a chemokine (CCL5/RANTES) by astrocytes. J Neurosci. (2008) 28:3277–90. 10.1523/JNEUROSCI.0116-08.2008 18367595 PMC6670608

[44] Tadesse A Gurfein BT Zhang Y Zameer A John GR. VEGF-mediated disruption of endothelial CLN-5 promotes blood-brain barrier breakdown. Proc Natl Acad Sci USA. (2009) 106:1977–82. 10.1073/pnas.0808698106 19174516 PMC2644149

[45] Galic MA Riazi K Pittman QJ. Cytokines and brain excitability. Front Neuroendocr. (2012) 33:116–25. 10.1016/j.yfrne.2011.12.002 22214786 PMC3547977

[46] Vezzani A Viviani B. Neuromodulatory properties of inflammatory cytokines and their impact on neuronal excitability. Neuropharmacology. (2015) 96:70–82. 10.1016/j.neuropharm.2014.10.027 25445483

[47] Sofroniew MV. Multiple roles for astrocytes as effectors of cytokines and inflammatory mediators. Neuroscientist. (2014) 20:160–72. 10.1177/1073858413504466 24106265

[48] Vezzani A Balosso S Ravizza T. Neuroinflammatory pathways as treatment targets and biomarkers in epilepsy. Nat Rev Neurol. (2019) 15:459–72. 10.1038/s41582-019-0217-x 31263255

[49] Lau LOKT Yu AC. Astrocytes produce and release interleukin-1, interleukin-6, tumor necrosis factor alpha and interferon-gamma following traumatic and metabolic injury. J Neurotrauma. (2001) 18:351–9. 11284554 10.1089/08977150151071035

[50] Hyvärinen T Hagman S Ristola M Sukki L Vijula K Kreutzer J Co-stimulation with IL-1β and TNF-α induces an inflammatory reactive astrocyte phenotype with neurosupportive characteristics in a human pluripotent stem cell model system. Sci Rep. (2019) 9:16944 10.1038/s41598-019-53414-9 31729450 PMC6858358

[51] Bianco F Pravettoni E Colombo A Möller T Matteoli M Bianco F. Astrocyte-derived ATP induces vesicle shedding and IL-1 beta release from microglia. J Immunol. (2005) 174:7268–77. 10.4049/jimmunol.174.11.7268 15905573

[52] Terrone G Balosso S Pauletti A Ravizza T Vezzani A. Inflammation and reactive oxygen species as disease modifiers in epilepsy. Neuropharmacology. (2019) 167:107742. 10.1016/j.neuropharm.2019.107742 31421074

[53] Minami M Kuraishi Y Satoh M. Effects of kainic acid on messenger RNA levels of IL-1b, IL-6, TNFa and LIFE in the rat brain. Biochem Biophys Res Commun. (1991) 176:593–8. 10.1016/s0006-291x(05)80225-6 1709015

[54] Vezzani A Conti M De Luigi A Ravizza T Moneta D Marchesi F. Interleukin-1β immunoreactivity and microglia are enhanced in the rat hippocampus by focal kainate application: functional evidence for enhancement of electrographic seizures. J Neurosci. (1999) 19:5054–65. 10.1523/jneurosci.19-12-05054.1999 10366638 PMC6782637

[55] Simoni MG De Perego C Ravizza T Moneta D Conti M Marchesi F. Inflammatory cytokines and related genes are induced in the rat hippocampus by limbic status epilepticus. Eur J Neurosci. (2000) 12:2623–33. 10947836 10.1046/j.1460-9568.2000.00140.x

[56] Merbl Y Sommer A Chai O Aroch I Zimmerman G Friedman A. Tumor necrosis factor- a and interleukin-6 concentrations in cerebrospinal fluid of dogs after seizures. J Vet Intern Med. (2014) 28:1775–81. 10.1111/jvim.12462 25308784 PMC4895630

[57] Patel DC Wallis G Dahle EJ McElroy PB Thomson KE Tesi RJ. Hippocampal TNFα signaling contributes to seizure generation in an infection-induced mouse model of limbic epilepsy. Disord Nerv Syst Hippocampal. (2017) 4:1–20. 10.1523/ENEURO.0105-17.20171 28497109 PMC5422919

[58] Semple BD Brien TJO Gimlin K Wright DK Kim SE Casillas-Espinosa PM. Interleukin-1 receptor in seizure susceptibility after traumatic injury to the pediatric brain. J Neurosci. (2017) 37:7864–77. 10.1523/JNEUROSCI.0982-17.2017 28724747 PMC5559762

[59] Kostic D Carlson R Henke D Rohn K Tipold A. Evaluation of IL-1β levels in epilepsy and traumatic brain injury in dogs. BMC Neurosci. (2019) 20:29. 10.1186/s12868-019-0509-5 31208341 PMC6580646

[60] Ichiyama T Nishikawa M Yoshitomi T Furukawa S Tumor necrosis factor-alfa, interleukin-1beta, and interleukin-6 in cerebrospinal fluid from children with prolonged febrile seizures comparison with acute encephalitis/encephalopathy. Neurology. (1998) 50:407–12. 9484363 10.1212/wnl.50.2.407

[61] Peltola J Palmio J Korhonen L Suhonen J Miettinen A. Interleukin-6 and interleukin-1 receptor antagonist in cerebrospinal fluid from patients with recent tonic-clonic seizures. Epilepsy Res. (2000) 41:205–11. 10962211 10.1016/s0920-1211(00)00140-6

[62] Uludag I Duksal T Tiftikcioglu B Zorlu Y Ozkaya F Guldal K. IL-1β, IL-6 and IL1Ra levels in temporal lobe epilepsy. Seizure Eur J Epilepsy. (2015) 26:22–5. 10.1016/j.seizure.2015.01.009 25799897

[63] Alapirtti T Lehtimäki K Nieminen R Mäkinen R Raitanen J Moilanen E. The production of IL-6 in acute epileptic seizure: a video-EEG study. J Neuroimmunol. (2017) 316:50–55. 10.1016/j.jneuroim.2017.12.008 29273305

[64] Wang SAM Cheng Q Malik S Yang JAY. Interleukin-1beta Inhibits gamma-aminobutyric acid type A (GABA A) receptor current in cultured hippocampal neurons. J Pharmacol Exp Ther. (2000) 292:497–504. 10640285

[65] Yang S Liu Z Wen L Qiao H Zhou W Zhang Y. Interleukin-1beta enhances NMDA receptor-mediated current but inhibits excitatory synaptic transmission. Brain Res. (2005) 1034:172–9. 10.1016/j.brainres.2004.11.018 15713269

[66] Viviani B Bartesaghi S Gardoni F Vezzani A Behrens MM Bartfai T. Interleukin-1β enhances NMDA receptor-mediated intracellular calcium increase through activation of the Src family of kinases. J Neurosci. (2003) 23:8692–700. 10.1523/jneurosci.23-25-08692.2003 14507968 PMC6740426

[67] Balosso S Maroso M Sanchez-Alavez M Ravizza T Frasca A Bartfai T. A novel non-transcriptional pathway mediates the proconvulsive effects of interleukin-1β. Brain. (2008) 131:3256–65. 10.1093/brain/awn271 18952671 PMC2724908

[68] Scheppingen J Van Iyer AM Prabowo AS Muhlebner A Giordano F Genitori L. Expression of MicroRNAs miR21, miR146a, and miR155 in tuberous sclerosis complex cortical tubers and their regulation in human astrocytes and SEGA-derived cell cultures. Glia. (2016) 64:1066–82. 10.1002/glia.22983 27014996

[69] Scheppingen J Van Mills JD Zimmer TS Broekaart DWM Iori V Bongaarts A. miR147b: a novel key regulator of interleukin 1 beta-mediated inflammation in human astrocytes. Glia. (2018) 66:1082–97. 10.1002/glia.23302 29384235

[70] Korotkov A Baayen JC Aronica E Banchaewa L Anink JJ Vliet EA. microRNA-132 is overexpressed in glia in temporal lobe epilepsy and reduces the expression of pro-epileptogenic factors in human cultured astrocytes. Glia. (2020) 68:60–75. 10.1002/glia.23700 31408236 PMC6899748

[71] Zurolo E de Groot M Iyer A Anink J van Vliet EA Heimans JJ. Regulation of Kir4.1 expression in astrocytes and astrocytic tumors: a role for interleukin-1 β. J Neuroinflamm. (2012) 9:1–17. 10.1186/1742-2094-9-280 23270518 PMC3538650

[72] Korotkov A Broekaart DWM Scheppingen J Van Anink JJ Baayen JC Idema S. Increased expression of matrix metalloproteinase 3 can be attenuated by inhibition of microRNA-155 in cultured human astrocytes. J Neuroinflam. (2018) 15:1–14. 10.1186/s12974-018-1245-y 30031401 PMC6054845

[73] Ye ZC Sontheimer H. Cytokine modulation of glial glutamate uptake: a possible involvement of nitric oxide. Neuroreport. (1996) 7:2181–5. 10.1097/00001756-199609020-00025 8930985

[74] Hu S Sheng WS Ehrlich LC Peterson PK Chao CC. Cytokine effects on glutamate uptake by human astrocytes. Neuroimmunomodulation. (2000) 7:153–9. 10.1159/000026433 10754403

[75] Pita I Jelaso AM Ide CF. IL-1β increases intracellular calcium through an IL-1 type 1 receptor mediated mechanism in C6 astrocytic cells. Int J Devl Neurosci. (1999) 17:813–20. 10.1016/S0736-5748(99)00063-5 10593617

[76] Sanz P Garcia-Gimeno MA. Reactive glia inflammatory signaling pathways and epilepsy. Int J Mol Sci. (2020) 21:1–17. 10.3390/ijms21114096 32521797 PMC7312833

[77] Zhang J Sadowska GB Chen X Park SY Kim JE Bodge CA. Anti-IL-6 neutralizing antibody modulates blood-brain barrier function in the ovine fetus. FASEB J. (2015) 29:1739–53. 10.1096/fj.14-258822 25609424 PMC4771067

[78] Fine SM Angel RA Seth W Epstein LG Rothstein JD Dewhurst S. Tumor necrosis factor α inhibits glutamate uptake by primary human astrocytes. J Biol Chem. (1996) 271:15303–6. 10.1074/jbc.271.26.15303 8663435

[79] Clark IA Vissel B. Excess cerebral TNF causing glutamate excitotoxicity rationalizes treatment of neurodegenerative diseases and neurogenic pain by anti-TNF agents. J Neuroinflamm. (2016) 13:1–16. 10.1186/s12974-016-0708-2 27596607 PMC5011997

[80] Bedner P Steinhäuser C. TNFα-driven astrocyte purinergic signaling during epileptogenesis. Trends Mol Med. (2019) 25:70–2. 10.1016/j.molmed.2018.12.001 30581052

[81] Stellwagen D Beattie EC Seo JY Malenka RC. Differential regulation of AMPA receptor and GABA receptor trafficking by tumor necrosis factor-α. J Neurosci. (2005) 25:3219–28. 10.1523/JNEUROSCI.4486-04.2005 15788779 PMC6725093

[82] Aronica E Crino PB. Inflammation in epilepsy: clinical observations. Epilepsia. (2011) 52:26–32. 10.1111/j.1528-1167.2011.03033.x 21542843

[83] Das A Iv GCW Holmes C Mcdowell ML Smith JA Marshall JD. Hippocampal tissue of patients with refractory temporal lobe epilepsy is associated with astrocyte activation, inflammation, and altered expression of channels and receptors. Neuroscience. (2012) 220:237–46. 10.1016/j.neuroscience.2012.06.002 22698689 PMC3412889

[84] Ravizza T Lucas S Balosso S Bernardino L Ku G No F. Inactivation of Caspase-1 in rodent brain : a novel anticonvulsive strategy. Epilepsia. (2006) 47:1160–8. 10.1111/j.1528-1167.2006.00590.x 16886979

[85] Maroso M Balosso S Ravizza T Iori V Wright CI French J. Interleukin-1β biosynthesis inhibition reduces acute seizures and drug resistant chronic epileptic activity in mice. J Am Soc Exp Neurother. (2011) 8:304–15. 10.1007/s13311-011-0039-z 21431948 PMC3101825

[86] Bialer M Johannessen SI Levy RH Perucca E Tomson T White HS. Progress report on new antiepileptic drugs: a summary of the eleventh eilat conference (EILAT XI). Epilepsy Res. (2013) 103:2–30. 10.1016/j.eplepsyres.2012.10.001 23219031

[87] Lafrance-Corey RG Ho M Muskardin TW. Super-refractory status epilepticus and febrile infection-related epilepsy syndrome treated with anakinra. Ann Neurol. (2016) 80:939–45. 10.1002/ana.24806.Super-refractory 27770579 PMC5225882

[88] Kenney-Jung DL Vezzani A Kahoud RJ Lafrance-Corey RG Ho M Muskardin TW FIRES induced status epilepticus treated with anakinra. Ann Neurol. (2016) 80:939–45. 10.1002/ana.24806 27770579 PMC5225882

[89] Dilena R Mauri E Aronica E Bernasconi P Bana C Cappelletti C. Therapeutic effect of Anakinra in the relapsing chronic phase of febrile infection – related epilepsy syndrome. Epilepsia Open. (2019) 4:344–50. 10.1002/epi4.12317 31168503 PMC6546072

[90] Westbrook C Subramaniam T Seagren RM Tarula E Co D Furstenberg-Knauff M. Febrile infection-related epilepsy syndrome (FIRES) treated successfully with anakinra in A 21-year-old woman. WMJ. (2019) 118:135–9. 10.1111/j.1528-1167.2011.03250.x 31682750 PMC7082129

[91] Jyonouchi H Geng L Intractable epilepsy (IE) and responses to anakinra, a human recombinant IL-1 receptor agonist (IL-1ra): case reports. J Clin Cell Immunol. (2016) 7:1–5. 10.4172/2155-9899.1000456

[92] Desena AD Do T Schulert GS. Systemic autoinflammation with intractable epilepsy managed with interleukin-1 blockade. J Neuroinflamm. (2018) 15:1–6. 10.1186/s12974-018-1063-2 29426321 PMC5807745

[93] Steinborn B Zarowksi M Winczewska-Wiktor A Wójcicka M Mlodzikowska-Albrecht J Losy J. Concentration of Il-1β, Il-2, Il-6, TNFα in the blood serum in children with generalized epilepsy treated by valproate. Pharmacol Rep. (2014) 66:972–5. 10.1016/j.pharep.2014.06.005 25443723

[94] Lagarde S Villeneuve N Lepine A Mcgonigal A Roubertie A Barthez MJ. Anti – tumor necrosis factor alpha therapy (adalimumab) in rasmussen's encephalitis: an open pilot study. Epilepsia. (2016) 57:956–66. 10.1111/epi.13387 27106864

[95] Frigerio F Pasqualini G Craparotta I Marchini S Vliet EA Van Foerch P. n-3 Docosapentaenoic acid-derived protectin D1 promotes resolution of neuroinflammation and arrests epileptogenesis. Brain. (2018) 141:3130–43. 10.1093/brain/awy247 30307467 PMC6202571

[96] Puttachary S Sharma S Verma S Yang Y Putra M Thippeswamy A. 1400W, a highly selective inducible nitric oxide synthase inhibitor is a potential disease modifier in the rat kainate model of temporal lobe epilepsy. Neurobiol Dis. (2016) 93:184–200. 10.1016/j.nbd.2016.05.013 27208748

[97] Boison D Aronica E. Comorbidities in neurology: is adenosine the common link? Neuropharmacology. (2015) 97:18–34. 10.1016/j.gde.2016.03.011 25979489 PMC4537378

[98] Pekny M Pekna M Messing A Steinhäuser C Lee JM Parpura V. Astrocytes: a central element in neurological diseases. Acta Neuropathol. (2016) 131:323–45. 10.1007/s00401-015-1513-1 26671410

[99] Binder DK. Astrocytes: stars of the sacred disease. Epilepsy Curr. (2018) 18:172–9. 10.5698/1535-7597.18.3.172 29950942 PMC6017684

[100] Eid T Thomas MJ Spencer DD Lai JCK Malthankar GV Kim JH. Loss of glutamine synthetase in the human epileptogenic hippocampus : possible mechanism for raised extracellular glutamate in mesial temporal lobe epilepsy. Lancet. (2004) 363:28–37. 10.1016/s0140-6736(03)15166-5 14723991

[101] Gouder N Scheurer L Fritschy J Boison D. Overexpression of adenosine kinase in epileptic hippocampus contributes to epileptogenesis. J Neurosci. (2004) 24:692–701. 10.1523/JNEUROSCI.4781-03.2004 14736855 PMC6729249

[102] Aronica E Zurolo E Iyer A Groot M De Anink J. Upregulation of adenosine kinase in astrocytes in experimental and human temporal lobe epilepsy. Epilepsia. (2012) 52:1645–55. 10.1111/j.1528-1167.2011.03115.x 21635241 PMC3169746

[103] Amiry-Moghaddam M Williamson A Palomba M Eid T Lanerolle NC De Nagelhus EA. Delayed K^+^ clearance associated with aquaporin-4 mislocalization : phenotypic defects in brains of alfa-syntrophin-null mice. Proc Natl Acad Sci USA. (2003) 100:13615–20. 10.1073/pnas.2336064100 14597704 PMC263862

[104] Binder DK Nagelhus EA Ottersen OLEP Aquaporin-4 and epilepsy. Glia. (2012) 1214:1203–14. 10.1002/glia.22317 22378467

[105] Bordey A Sontheimer H. Properties of human glial cells associated with epileptic seizure *foci*. Epilepsy Res. (1998) 32:286–303. 9761328 10.1016/s0920-1211(98)00059-x

[106] Heuser K Eid T Lauritzen F Thoren AE Vindedal GF Tauboll E. Loss of perivascular kir4.1 potassium channels in the sclerotic hippocampus of patients with mesial temporal lobe epilepsy. J Neuropathol Exp Neurol. (2012) 71:814–25. 10.1097/NEN.0b013e318267b5af 22878665 PMC3470834

[107] Lauritzen F Heuser K Lanerolle NCDE Lee TW Spencer DD Kim JH. Redistribution of monocarboxylate transporter 2 on the surface of astrocytes in the human epileptogenic *hippocampus*. Gila. (2012) 1181:1172–81. 10.1002/glia.22344 22535546 PMC3664041

[108] Lauritzen F Perez EL Melillo ER Roh J Zaveri HP Lee TW. Neurobiology of disease altered expression of brain monocarboxylate transporter 1 in models of temporal lobe epilepsy. Neurobiol Dis. (2012) 45:165–76. 10.1016/j.nbd.2011.08.001 21856423 PMC3351090

[109] Escartin C Pierre K Colin A Brouillet E Delzescaux T Guillermier M. Activation of astrocytes by CNTF induces metabolic plasticity and increases resistance to metabolic insults. J Neurosci. (2007) 27:7094–104. 10.1523/JNEUROSCI.0174-07.2007 17611262 PMC6794576

[110] Gavillet M Allaman I Magistretti PJ. Modulation of astrocytic metabolic phenotype by proinflammatory cytokines. Glia. (2008) 56:975–89. 10.1002/glia.20671 18383346

[111] Valenza M Leoni V Karasinska JM Petricca L Fan J Carroll J. Cholesterol defect is marked across multiple rodent models of huntington's disease and is manifest in astrocytes. J Neurosci. (2010) 30:10844–50. 10.1523/JNEUROSCI.0917-10.2010 20702713 PMC3842469

[112] Rossi D Volterra A. Astrocytic dysfunction: Insights on the role in neurodegeneration. Brain Res Bull. (2009) 80:224–32. 10.1016/j.brainresbull.2009.07.012 19631259

[113] Escartin C Rouach N. Astroglial networking contributes to neurometabolic coupling. Front Neuroenergetics. (2013) 5:4. 10.3389/fnene.2013.00004 23637659 PMC3636502

[114] Pannasch U Rouach N. Emerging role for astroglial networks in information processing: from synapse to behavior. Trends Neurosci. (2013) 36:405–17. 10.1016/j.tins.2013.04.004 23659852

[115] Escartin C Brouillet E Gubellini P Trioulier Y Jacquard C Smadja C. Ciliary neurotrophic factor activates astrocytes, redistributes their glutamate transporters GLAST and GLT-1 to raft microdomains, and improves glutamate handling *in vivo*. J Neurosci. (2006) 26:5978–89. 10.1523/JNEUROSCI.0302-06.2006 16738240 PMC6675222

[116] Sheldon AL Robinson MB. The role of glutamate transporters in neurodegenerative diseases and potential opportunities for intervention. Neurochem Int. (2007) 51:333–55. 10.1016/j.neuint.2007.03.012 17517448 PMC2075474

[117] Tian G Azmi H Takano T Xu Q Peng W Lin J. An astrocytic basis of epilepsy. Nat Med. (2005) 11:973–81. 10.1038/nm1277 16116433 PMC1850946

[118] Sofroniew MV. Molecular dissection of reactive astrogliosis and glial scar formation. Trends Neurosci. (2010) 32:638–47. 10.1016/j.tins.2009.08.002.Molecular 19782411 PMC2787735

[119] Liddelow SA Barres BA. Reactive astrocytes: production, function, and therapeutic potential. Immunity. (2017) 46:957–67. 10.1016/j.immuni.2017.06.006 28636962

[120] Jha MK Jo M Kim JH Suk K. Microglia-astrocyte crosstalk: an intimate molecular conversation. Neuroscientist. (2019) 25:227–40. 10.1177/1073858418783959 29931997

[121] Domingues HS Portugal CC Socodato R Relvas JB Oligodendrocyte, astrocyte, and microglia crosstalk in myelin development, damage, and repair. Front Cell Dev Biol. (2016) 4:71 10.3389/fcell.2016.00071 27551677 PMC4923166

[122] Vainchtein ID Molofsky AV. Astrocytes and microglia: in sickness and in health. Trends Neurosci. (2020) 43:144–54. 10.1016/j.tins.2020.01.003 32044129 PMC7472912

[123] Burda JE Sofroniew MV. Seducing astrocytes to the dark side. Cell Res. (2017) 27:726–7. 10.1038/cr.2017.37 28303889 PMC5518868

[124] Liddelow SA Guttenplan KA Clarke LE Bennett FC Bohlen CJ Schirmer L. Neurotoxic reactive astrocytes are induced by activated microglia. Nature. (2017) 541:481–7. 10.1038/nature21029 28099414 PMC5404890

[125] Heiss CN Olofsson LE. The role of the gut microbiota in development, function and disorders of the central nervous system and the enteric nervous system. J Neuroendocrinol. (2019) 31:1–11. 10.1111/jne.12684 30614568

[126] Ma Q Xing C Long W Wang HY Liu Q Wang RF. Impact of microbiota on central nervous system and neurological diseases: the gut-brain axis. J Neuroinflamm. (2019) 16:1–14. 10.1186/s12974-019-1434-3 30823925 PMC6397457

[127] Wikoff WR Anfora AT Liu J Schultz PG Lesley SA Peters EC. Metabolomics analysis reveals large effects of gut microflora on mammalian blood metabolites. Proc Natl Acad Sci USA. (2009) 106:3698–703. 10.1073/pnas.0812874106 19234110 PMC2656143

[128] Zhang J Yu C Zhang X Chen H Dong J Lu W. Porphyromonas gingivalis lipopolysaccharide induces cognitive dysfunction, mediated by neuronal inflammation via activation of the TLR4 signaling pathway in C57BL/6 mice. J Neuroinflamm. (2018) 15:1–14. 10.1186/s12974-017-1052-x 29426327 PMC5810193

[129] Bélanger M Allaman I Magistretti PJ. Brain energy metabolism : focus on astrocyte-neuron metabolic cooperation. Cell Metab. (2011) 14:724–38. 10.1016/j.cmet.2011.08.016 22152301

[130] Brown AM Ransom BR. Astrocyte glycogen and brain energy metabolism. Glia. (2007) 1271:1263–71. 10.1002/glia.20557 17659525

[131] Suh SW Bergher JP Anderson CM Treadway JL Fosgerau K Swanson RA. Astrocyte glycogen sustains neuronal activity during hypoglycemia: studies with the glycogen phosphorylase inhibitor CP-316,819 ([R-R^*^,S^*^]-5-Chloro-N-[2-hydroxy-3-(methoxymethylamino)-3-oxo-1-(phenylmethyl)propyl]-1H-indole-2-carboxamide). J Pharmacol Exp Ther. (2007) 321:45–50. 10.1124/jpet.106.115550 17251391

[132] Pellerin L Halestrap AP Pierre K Cellular and subcellular distribution of monocarboxylate transporters in cultured brain cells and in the adult brain. J Neurosci Res. (2005) 64:55–64. 10.1002/jnr.20307 15573400

[133] Phelps CREIGHTONH. Barbiturate-induced glycogen accumulation in brain. an electron microscopic study. Brain Res. (1972) 39:225–34. 10.1016/0006-8993(72)90797-4 5025645

[134] Pfeiffer-Guglielmi B Fleckenstein B Hamprecht B. Immunocytochemical localization of glycogen phosphorylase isozymes in rat nervous tissues by using isozyme-specific antibodies. J Neurochem. (2003) 85:73–81. 10.1046/j.1471-4159.2003.01644.x 12641728

[135] Schwartz TH. Neurovascular coupling and epilepsy: hemodynamic markers for localizing and predicting seizure onset. Epilepsy Curr. (2007) 7:91–4. 10.1111/j.1535-7511.2007.00183.x 17694162 PMC1941907

[136] Wong M. Astrocyte networks and epilepsy: when stars collide. Curr Lit Basic Sci. (2009) 9:113–5. 10.1111/j.1535-7511.2009.01310.x 19693329 PMC2728489

[137] Greene AE Todorova MT Seyfried TN. Perspectives on the metabolic management of epilepsy through dietary reduction of glucose and elevation of ketone bodies. J Neurochem. (2003) 86:529–37. 10.1046/j.1471-4159.2003.01862.x 12859666

[138] Henderson CB Filloux FM Alder SC Lyon JL Caplin DA. Efficacy of the ketogenic diet as a treatment option for epilepsy : meta-analysis. J Child Neurol. (2006) 21:193–8. 10.2310/7010.2006.00044 16901419

[139] Kossoff EH Rho JM. Ketogenic diets : evidence for short- and long-term efficacy. Am Soc Exp Neurother. (2009) 6:406–14. 10.1016/j.nurt.2009.01.005 19332337 PMC4071763

[140] Rho JM. How does the ketogenic diet induce anti-seizure effects? Neurosci Lett. (2017) 637:4–10. 10.1016/j.neulet.2015.07.034 26222258

[141] Kossoff EH Dorward JL The modified atkins diet. Epilepsia. (2008) 49:37–41. 10.1111/j.1528-1167.2008.01831.x 19049584

[142] Pfeifer HH Lyczkowski DA Thiele EA. Low glycemic index treatment : implementation and new insights into efficacy. Epilepsia. (2008) 49:42–5. 10.1111/j.1528-1167.2008.01832.x 19049585

[143] Guzel O Uysal U Arslan N. Efficacy and tolerability of olive oil-based ketogenic diet in children with drug-resistant epilepsy: a single center experience from Turkey. Eur J Paediatr Neurol. (2019) 23:143–51. 10.1016/j.ejpn.2018.11.007 30497921

[144] Masino SA Rho JM. Metabolism and epilepsy: ketogenic diets as a homeostatic link. Brain Res. (2019) 1703:26–30. 10.1016/j.brainres.2018.05.049 29883626 PMC6281876

[145] Liu H Yang Y Wang Y Tang H Zhang F Zhang Y. Ketogenic diet for treatment of intractable epilepsy in adults: a meta-analysis of observational studies. Epilepsia Open. (2018) 3:9–17. 10.1002/epi4.12098 29588983 PMC5839310

[146] Garriga-Canut M Schoenike B Qazi R Bergendahl K Daley TJ Pfender RM. 2-Deoxy-D-glucose reduces epilepsy progression by NRSF-CtBP–dependent metabolic regulation of chromatin structure. Nat Neurosci. (2006) 9:1382–7. 10.1038/nn1791 17041593

[147] Stafstrom CE Ockuly JC Murphree L Valley MT Roopra A Sutula TP. Anticonvulsant and antiepileptic actions of 2-deoxy-D-glucose in epilepsy models. Ann Neurol. (2009) 65:435–47. 10.1002/ana.21603 19399874 PMC2910719

[148] Ockuly JC Gielissen JM Levenick CV Zeal C Groble K Munsey K. Behavioral, cognitive, and safety profile of 2-deoxy-2-glucose (2DG) in adult rats. Epilepsy Res. (2012) 101:246–52. 10.1016/j.eplepsyres.2012.04.012 22578658

[149] Sada N Lee S Katsu T Otsuki T Inoue T. Targeting LDH enzymes with a stiripentol analog to treat epilepsy. Science. (2015) 347:1362–7. 10.1126/science.aaa1299 25792327

[150] Rouach N Koulakoff A Abudara V Willecke K Giaume C. Astroglial metabolic networks sustain hippocampal synaptic transmission. Science. (2008) 322:1551–6. 10.1126/science.1164022 19056987

[151] Dermietzel R Spray DC. Gap jundions in the brain: where, what type, how many and why? TINS. (1993) 16:186–92. 7685944 10.1016/0166-2236(93)90151-b

[152] Dermietzel R Gao Y Scemes E Vieira D Urban M Kremer M. Connexin43 null mice reveal that astrocytes express multiple connexins. Brain Res Rev. (2000) 32:45–56. 10.1016/s0165-0173(99)00067-3 10751656

[153] Parpura V Verkhratsky A. Homeostatic function of astrocytes: Ca^2+^ and Na^+^ signalling. Transl Neurosci. (2012) 3:334–44. 10.2478/s13380-012-0040-y 23243501 PMC3520132

[154] Stout CE Costantin JL Naus CCG Charles AC. Intercellular calcium signaling in astrocytes via ATP release through connexin hemichannels. J Biol Chem. (2002) 277:10482–8. 10.1074/jbc.M109902200 11790776

[155] Strohschein S Uttmann KH Gabriel S Binder DK. Impact of aquaporin-4 channels on K^+^ buffering and gap junction coupling in the hippocampus. Glia. (2011) 980:973–80. 10.1002/glia.21169 21446052

[156] Wallraff A Heinemann U Theis M Willecke K Steinha C. The impact of astrocytic gap junctional coupling on potassium buffering in the hippocampus. J Neurosci. (2006) 26:5438–47. 10.1523/JNEUROSCI.0037-06.2006 16707796 PMC6675300

[157] Huguet G Joglekar A Messi LM Buckalew R Wong S Terman D. Neuroprotective role of gap junctions in a neuron astrocyte network model. Biophys J. (2016) 111:452–62. 10.1016/j.bpj.2016.05.051 27463146 PMC4968398

[158] Mu J Herde MK Bedner P Dupper A Hu K Haas CA. Astrocyte uncoupling as a cause of human temporal lobe epilepsy. Brain. (2015) 138:1208–22. 10.1093/brain/awv067 25765328 PMC5963418

[159] Crunelli V Carmignoto G Steinhäuser C. Novel astrocyte targets : new avenues for the therapeutic treatment of epilepsy. Neurosci. (2015) 21:62–83. 10.1177/1073858414523320 24609207 PMC4361461

[160] Li Q Li Q-Q Jia J-N Liu Z-Q Zhou H-H Mao X-Y. Targeting gap junction in epilepsy: perspectives and challenges. Biomed Pharmacother. (2019) 109:57–65. 10.1016/j.biopha.2018.10.068 30396092

[161] Deshpande T Li T Herde MK Becker A Vatter H Schwarz MK. Subcellular reorganization and altered phosphorylation of the astrocytic gap junction protein connexin43 in human and experimental temporal lobe epilepsy. Glia. (2017) 65:1809–20. 10.1002/glia.23196 28795432

[162] Losi G Chiavegato A Zonta M Brondi M Vetri F Uva L. An excitatory loop with astrocytes contributes to drive neurons to seizure threshold. PLoS Biol. (2010) 8:e1000352. 10.1371/journal.pbio.1000352 20405049 PMC2854117

[163] Jacobson GM Voss LJ Melin SM Mason JP Cursons RT Steyn-Ross DA. Connexin36 knockout mice display increased sensitivity to pentylenetetrazol-induced seizure-like behaviors. Brain Res. (2010) 1360:198–204. 10.1016/j.brainres.2010.09.006 20833151

[164] Curti S Hoge G Nagy JI Pereda AE. Synergy between electrical coupling and membrane properties promotes strong synchronization of neurons of the mesencephalic trigeminal nucleus. J Neurosci. (2012) 32:4341–59. 10.1523/JNEUROSCI.6216-11.2012 22457486 PMC3339267

[165] Hosseinzadeh H Nassiri Asl M. Anticonvulsant, sedative and muscle relaxant effects of carbenoxolone in mice. BMC Pharmacol. (2003) 3:3. 10.1186/1471-2210-3-3 12720572 PMC156636

[166] Ventura-Mejía C Medina-Ceja L. Decreased fast ripples in the hippocampus of rats with spontaneous recurrent seizures treated with carbenoxolone and quinine. Biomed Res Int. (2014) 2014:282490. 10.1155/2014/282490 25276773 PMC4168142

[167] Ran X Xiang J Song P Jiang L Liu B. Effects of gap junctions blockers on fast ripples and connexin in rat hippocampi after status epilepticus. Epilepsy Res. (2018) 146:28–35. 10.1016/j.eplepsyres.2018.07.010 30056318

[168] Franco-Pérez J Ballesteros-Zebadúa P Manjarrez-Marmolejo J. Anticonvulsant effects of mefloquine on generalized tonic-clonic seizures induced by two acute models in rats. BMC Neurosci. (2015) 16:7. 10.1186/s12868-015-0145-7 25886955 PMC4411716

[169] Nassiri-Asl M Zamansoltani F Torabinejad B. Antiepileptic effects of quinine in the pentylenetetrazole model of seizure. Seizure. (2009) 18:129–32. 10.1016/j.seizure.2008.08.002 18786839

[170] Manjarrez-Marmolejo J Franco-Pérez J. Gap Junction blockers : an overview of their effects on induced seizures in animal models. Curr Neuropharmacol. (2016) 14:759–71. 10.2174/1570159X14666160603115 27262601 PMC5050393

[171] Venance L Plomellit D Glowinski J Giaume C. Inhibition by anandamide of gap junctions and intercellular calcium signalling in striatal astrocytes. Nature. (1995) 376:590–4. 10.1038/376590a0 7637807

[172] Guan X Cravatt BF Ehring GR Hall JE Boger DL Lerner RA. The sleep-inducing lipid oleamide deconvolutes gap junction communication and calcium wave transmission in glial cells. J Cell Biol. (1997) 139:1785–92. 10.1083/jcb.139.7.1785 9412472 PMC2132638

[173] Lambert DM Vandevoorde S Diependaele G Govaerts SJ Robert AR Anticonvulsant activity of N-palmitoylethanolamide, a putative endocannabinoid, in mice. Epilepsia. (2001) 42:321–7. 10.1046/j.1528-1157.2001.41499.x 11442148

[174] Wallace MJ Martin BR DeLorenzo RJ. Evidence for a physiological role of endocannabinoids in the modulation of seizure threshold and severity. Eur J Pharmacol. (2002) 452:295–301. 10.1016/S0014-2999(02)02331-2 12359270

[175] Wu C-F Li C-L Song H-R Zhang H-F Yang J-Y Wang Y-L. Selective effect of oleamide, an endogenous sleepinducing lipid amide, on pentylenetetrazole-induced seizures in mice. J Pharm Pharmacol. (2003) 55:1159–62. 10.1211/0022357021431 12956907

[176] Solomonia R Nozadze M Mikautadze E Kuchiashvili N Kiguradze T Abkhazava D. Effect of oleamide on pentylenetetrazole-induced seizures in rats. Bull Exp Biol Med. (2008) 145:225–7. 10.1007/s10517-008-0056-z 19023975

[177] Parpura V Basarsky TA Liu F Jeftinija K Jeftinija S Haydon PG. Glutamate-mediated astrocyte-neuron signalling. Nature. (1994) 369:744–7. 10.1038/369744a0 7911978

[178] Angulo MC Kozlov AS Charpak S Audinat E. Glutamate released from glial cells synchronizes neuronal activity in the hippocampus. J Neurosci. (2004) 24:6920–7. 10.1523/JNEUROSCI.0473-04.2004 15295027 PMC6729611

[179] Fellin T Carmignoto G. Neurone-to-astrocyte signalling in the brain represents a distinct multifunctional unit. J Physiol. (2004) 559:3–15. 10.1113/jphysiol.2004.063214 15218071 PMC1665073

[180] Schell MJ Brady RO Molliver ME Snyder SH. D-serine as a neuromodulator: regional and developmental localizations in rat brain glia resemble NMDA receptors. J Neurosci. (1997) 17:1604–15. 10.1523/jneurosci.17-05-01604.1997 9030620 PMC6573391

[181] Beltrán-Castillo S Olivares MJ Contreras RA Zúñiga G Llona I Von Bernhardi R. D-serine released by astrocytes in brainstem regulates breathing response to CO_2_ levels. Nat Commun. (2017) 8:838. 10.1038/s41467-017-00960-3 29018191 PMC5635109

[182] Barakat L Bordey A. GAT-1 and reversible GABA transport in bergmann glia in slices. J Neurophysiol. (2002) 88:1407–19. 10.1152/jn.2002.88.3.1407 12205162

[183] Jiménez-González C Pirttimaki T Cope DW Parri HR. Non-neuronal, slow GABA signalling in the ventrobasal thalamus targets δ-subunit-containing GABAA receptors. Eur J Neurosci. (2011) 33:1471–82. 10.1111/j.1460-9568.2011.07645.x 21395866 PMC3110310

[184] Yoon BE Lee CJ. GABA as a rising gliotransmitter. Front Neural Circuits. (2014) 8:141. 10.3389/fncir.2014.00141 25565970 PMC4269106

[185] Eulenburg V Gomeza J. Neurotransmitter transporters expressed in glial cells as regulators of synapse function. Brain Res Rev. (2010) 63:103–12. 10.1016/j.brainresrev.2010.01.003 20097227

[186] Bardóczi Z Pál B Koszeghy Á Wilheim T Watanabe M Záborszky L. Glycinergic input to the mouse basal forebrain cholinergic neurons. J Neurosci. (2017) 37:9534–49. 10.1523/JNEUROSCI.3348-16.2017 28874448 PMC5618268

[187] Shibasaki K Hosoi N Kaneko R Tominaga M Yamada K. Glycine release from astrocytes via functional reversal of GlyT1. J Neurochem. (2017) 140:395–403. 10.1111/jnc.13741 27419919

[188] Newman EA. Glial cell inhibition of neurons by release of ATP. J Neurosci. (2003) 23:1659–66. 10.1523/jneurosci.23-05-01659.2003 12629170 PMC2322877

[189] Zhang JM Wang HK Ye CQ Ge W Chen Y Jiang ZL. ATP released by astrocytes mediates glutamatergic activity-dependent heterosynaptic suppression. Neuron. (2003) 40:971–82. 10.1016/S0896-6273(03)00717-7 14659095

[190] Gordon GRJ Baimoukhametova DV Hewitt SA Rajapaksha WRAKJS Fisher TE Bains JS. Norepinephrine triggers release of glial ATP to increase postsynaptic efficacy. Nat Neurosci. (2005) 8:1078–86. 10.1038/nn1498 15995701

[191] Pellerin L Magistretti PJ. Glutamate uptake into astrocytes stimulates aerobic glycolysis: a mechanism coupling neuronal activity to glucose utilization. Proc Natl Acad Sci USA. (1994) 91:10625–9. 10.1073/pnas.91.22.10625 7938003 PMC45074

[192] Tang F Lane S Korsak A Paton JFR Gourine AV Kasparov S. Lactate-mediated glia-neuronal signalling in the mammalian brain. Nat Commun. (2014) 5:3284. 10.1038/ncomms4284 24518663 PMC3926012

[193] DiNuzzo M. Astrocyte-neuron interactions during learning may occur by lactate signaling rather than metabolism. Front Integr Neurosci. (2016) 10:2. 10.3389/fnint.2016.00002 26858613 PMC4731513

[194] Kimelberg HK Goderie SK Higman S Pang S Waniewski RA. Swelling-induced release of glutamate, aspartate, and taurine from astrocyte cultures. J Neurosci. (1990) 10:1583–91. 10.1523/jneurosci.10-05-01583.1990 1970603 PMC6570070

[195] Choe KY Olson JE Bourque CW. Taurine release by astrocytes modulates osmosensitive glycine receptor tone and excitability in the adult supraoptic nucleus. J Neurosci. (2012) 32:12518–27. 10.1523/JNEUROSCI.1380-12.2012 22956842 PMC6621246

[196] Do KQ Benz B Sorg O Pellerin L Magistretti PJ. β-Adrenergic stimulation promotes homocysteic acid release from astrocyte cultures: evidence for a role of astrocytes in the modulation of synaptic transmission. J Neurochem. (1997) 68:2386–94. 10.1046/j.1471-4159.1997.68062386.x 9166732

[197] Benz B Grima G Do KQ. Glutamate-induced homocysteic acid release from astrocytes: Possible implication in glia-neuron signaling. Neuroscience. (2004) 124:377–86. 10.1016/j.neuroscience.2003.08.067 14980387

[198] Krzan M Stenovec M Kreft M Pangršič T Grilc S Haydon PG. Calcium-dependent exocytosis of atrial natriuretic peptide from astrocytes. J Neurosci. (2003) 23:1580–3. 10.1523/jneurosci.23-05-01580.2003 12629160 PMC6741952

[199] Desai NS Rutherford LC Turrigiano GG. BDNF regulates the intrinsic excitability of cortical neurons. Learn Mem. (1999) 6:284–91. 10.1101/lm.6.3.284 10492010 PMC311300

[200] Jean YY Lercher LD Dreyfus CF. Glutamate elicits release of BDNF from basal forebrain astrocytes in a process dependent on metabotropic receptors and the PLC pathway. Neuron Glia Biol. (2008) 4:35–42. 10.1017/S1740925X09000052 19267952

[201] Dani JW Chernjavsky A Smith SJ. Neuronal activity triggers calcium waves in hippocampal astrocyte networks. Neuron. (1992) 8:429–40. 10.1016/0896-6273(92)90271-e 1347996

[202] Porter JT McCarthy KD. Hippocampal astrocytes *in situ* respond to glutamate released from synaptic terminals. J Neurosci. (1996) 16:5073–81. 10.1523/JNEUROSCI.16-16-05073.1996 8756437 PMC6579292

[203] Pasti L Volterra A Pozzan T Carmignoto G. Intracellular calcium oscillations in astrocytes: A highly plastic, bidirectional form of communication between neurons and astrocytes *in situ*. J Neurosci. (1997) 17:7817–30. 10.1523/jneurosci.17-20-07817.1997 9315902 PMC6793927

[204] Wang X Lou N Xu Q Tian GF Peng WG Han X. Astrocytic Ca^2+^ signaling evoked by sensory stimulation *in vivo*. Nat Neurosci. (2006) 9:816–23. 10.1038/nn1703 16699507

[205] Takata N Hirase H. Cortical layer 1 and layer 2/3 astrocytes exhibit distinct calcium dynamics *in vivo*. PLoS ONE. (2008) 3:e2525. 10.1371/journal.pone.0002525 18575586 PMC2424136

[206] Perea G Navarrete M Araque A. Tripartite synapses: astrocytes process and control synaptic information. Trends Neurosci. (2009) 32:421–31. 10.1016/j.tins.2009.05.001 19615761

[207] Nimmerjahn A Kirchhoff F Kerr JND Helmchen F. Sulforhodamine 101 as a specific marker of astroglia in the neocortex *in vivo*. Nat Methods. (2004) 1:31–7. 10.1038/nmeth706 15782150

[208] Hoogland TM Kuhn B Gobel W Huang W Nakai J Helmchen F. Radially expanding transglial calcium waves in the intact cerebellum. Proc Natl Acad Sci USA. (2009) 106:3496–501. 10.1073/pnas.0809269106 19211787 PMC2651231

[209] Kuga N Sasaki T Takahara Y Matsuki N Ikegaya Y. Large-scale calcium waves traveling through astrocytic networks *in vivo*. J Neurosci. (2011) 31:2607–14. 10.1523/JNEUROSCI.5319-10.2011 21325528 PMC6623677

[210] Savtchouk I Volterra A. Gliotransmission: beyond black and white. J Neurosci. (2018) 38:14–25. 10.1523/JNEUROSCI.0017-17.2017 29298905 PMC6705815

[211] Fiacco TA McCarthy KD Multiple lines of evidence indicate that gliotransmission does not occur under physiological conditions. J Neurosci. (2018) 38:3–13. 10.1523/JNEUROSCI.0016-17.2017 29298904 PMC5761435

[212] Hubbard JA Binder DK (2010). Astrocytes and *epilepsy*. Neurotherapeutics. (2010) 7:424–38. 10.1016/j.nurt.2010.08.002 20880506 PMC5084304

[213] Volterra A Liaudet N Savtchouk I. Astrocyte Ca^2+^ signalling: an unexpected complexity. Nat Neurosci. (2014) 15:327–35. 10.1038/nrn3725 24739787

[214] Araque A Parpura V Sanzgiri RP Haydon PG. Tripartite synapses: glia, the unacknowledged partner. Trends Neurosci. (1999) 22:208–15. 10.1016/S0166-2236(98)01349-6 10322493

[215] Santello M Calì C Bezzi P Synaptic plasticity: dynamics, development and disease. In: Kreutz MR Sala C editors. Advances in Experimental Medicine and Biology. Vienna: Springer (2012). p. 945–61. 10.1007/978-3-7091-0932-8_14

[216] Arizono M Inavalli VVGK Panatier A Pfeiffer T Angibaud J Levet F Structural basis of astrocytic Ca^2+^ signals at tripartite synapses. Nat Commun. (2020) 11:1906 10.1038/s41467-020-15648-4 32312988 PMC7170846

[217] Lorenzo J Vuillaume R Binczak S Jacquir S. Spatiotemporal model of tripartite synapse with perinodal astrocytic process. J Comput Neurosci. (2020) 48:1–20. 10.1007/s10827-019-00734-4 31797200

[218] Halassa MM Fellin T Haydon PG. The tripartite synapse: roles for gliotransmission in health and disease. Trends Mol Med. (2007) 13:54–63. 10.1016/j.molmed.2006.12.005 17207662

[219] Jourdain P Bergersen LH Bhaukaurally K Bezzi P Santello M Domercq M. Glutamate exocytosis from astrocytes controls synaptic strength. Nat Neurosci. (2007) 10:331–9. 10.1038/nn1849 17310248

[220] Woo DH Han K Shim JW Yoon B Kim E Bae JY. TREK-1 and best1 channels mediate fast and slow glutamate release in astrocytes upon GPCR activation. Cell. (2012) 151:25–40. 10.1016/j.cell.2012.09.005 23021213

[221] Park H Han KS Oh SJ Jo S Woo J Yoon BE. High glutamate permeability and distal localization of Best1 channel in CA1 hippocampal astrocyte. Mol Brain. (2013) 6:1–9. 10.1186/1756-6606-6-54 24321245 PMC4029177

[222] Yang Y Ge W Chen Y Zhang Z Shen W Wu C. Contribution of astrocytes to hippocampal long-term potentiation through release of D-serine. Proc Natl Acad Sci USA. (2003) 100:15194–9. 10.1073/pnas.2431073100 14638938 PMC299953

[223] Mothet JP Pollegioni L Ouanounou G Martineau M Fossier P Baux G. Glutamate receptor activation triggers a calcium-dependent and SNARE protein-dependent release of the gliotransmitter D-serine. Proc Natl Acad Sci USA. (2005) 102:5606–11. 10.1073/pnas.0408483102 15800046 PMC556243

[224] Martineau M Shi T Puyal J Knolhoff AM Dulong J Gasnier B. Storage and uptake of D-serine into astrocytic synaptic-like vesicles specify gliotransmission. J Neurosci. (2013) 33:3413–23. 10.1523/JNEUROSCI.3497-12.2013 23426669 PMC3772647

[225] Pascual O Casper KB Kubera C Zhang J Revilla R Sul J. Astrocytic purinergic signaling coordinates synaptic networks. Science. (2005) 310:113–6. 10.1126/science.1116916 16210541

[226] Lalo U Palygin O Rasooli-Nejad S Andrew J Haydon PG Pankratov Y. Exocytosis of ATP from astrocytes modulates phasic and tonic inhibition in the neocortex. PLoS Biol. (2014) 12:e1001747. 10.1371/journal.pbio.1001747 24409095 PMC3883644

[227] Bazargani N Attwell D. Astrocyte calcium signaling : the third wave. Nat Neurosci. (2016) 19:182–9. 10.1038/nn.4201 26814587

[228] Xiong Y Sun S Teng S Jin M Zhou Z. Ca^2+^-Dependent and Ca^2+^-independent ATP release in astrocytes. Front Mol Neurosci. (2018) 11:224. 10.3389/fnmol.2018.00224 30079012 PMC6063199

[229] Heinrich A Andõ RD Túri G Rõzsa B Sperlágh B. K^+^ depolarization evokes ATP, adenosine and glutamate release from glia in rat hippocampus: a microelectrode biosensor study. Br J Pharmacol. (2012) 167:1003–20. 10.1111/j.1476-5381.2012.01932.x 22394324 PMC3492983

[230] Orellana JA Froger N Ezan P Jiang JX Bennet MVL Naus CC. ATP and glutamate released via astroglial connexin 43 hemichannels mediate neuronal death through activation of pannexin 1 hemichannels. J Neurochem. (2011) 118:826–40. 10.1111/j.1471-4159.2011.07210.x 21294731 PMC3108012

[231] Stehberg J Moraga-Amaro R Salazar C Becerra A Echeverría C Orellana JA. Release of gliotransmitters through astroglial connexin 43 hemichannels is necessary for fear memory consolidation in the basolateral amygdala. FASEB J. (2012) 26:3649–57. 10.1096/fj.11-198416 22665389

[232] Szatkowski M Barbour B Attwell D. Non-vesicular release of glutamate from glial cells by reversed electrogenic glutamate uptake. Nature. (1990) 348:443–6. 10.1038/348443a0 2247147

[233] Rossi DJ Oshima T Attwell D. Glutamate release in severe brain ischaemia is mainly by reversed uptake. Nature. (2000) 403:316–21. 10.1038/35002090 10659851

[234] Abudara V Retamal MA Del Rio R Orellana JA. Synaptic functions of hemichannels and pannexons: A double-edged sword. Front Mol Neurosci. (2018) 11:435. 10.3389/fnmol.2018.00435 30564096 PMC6288452

[235] Warr O Takahashi M Attwell D. Modulation of extracellular glutamate concentration in rat brain slices by cystine-glutamate exchange. J Physiol. (1999) 514:783–93. 10.1111/j.1469-7793.1999.783ad.x 9882750 PMC2269108

[236] Bridges RJ Natale NR Patel SA. System x c- cystine/glutamate antiporter: an update on molecular pharmacology and roles within the CNS. Br J Pharmacol. (2012) 165:20–34. 10.1111/j.1476-5381.2011.01480.x 21564084 PMC3252963

[237] Duan S Anderson CM Keung EC Chen Y Chen Y Swanson RA. P2X 7 receptor-mediated release of excitatory amino acids from astrocytes. J Neurosci. (2003) 23:1320–8. 10.1523/JNEUROSCI.23-04-01320.2003 12598620 PMC6742264

[238] Iglesias R Dahl G Qiu F Spray DC Scemes E. Pannexin 1: the molecular substrate of astrocyte “hemichannels.” J Neurosci. (2009) 29:7092–7. 10.1523/JNEUROSCI.6062-08.2009 19474335 PMC2733788

[239] Kang J Kang N Lovatt D Torres A Zhao Z Lin J. Connexin 43 hemichannels are permeable to ATP. J Neurosci. (2008) 28:4702–11. 10.1523/JNEUROSCI.5048-07.2008 18448647 PMC3638995

[240] Suadicani SO Brosnan CF Scemes E. P2X7 receptors mediate ATP release and amplification of astrocytic intercellular Ca^2+^ signaling. J Neurosci. (2006) 26:1378–85. 10.1523/JNEUROSCI.3902-05.2006 16452661 PMC2586295

[241] Xiong Y Teng S Zheng L Sun S Li J Guo N. Stretch-induced Ca^2+^ independent ATP release in hippocampal astrocytes. J Physiol. (2018) 596:1931–47. 10.1113/JP275805 29488635 PMC5978314

[242] Mylvaganam S Zhang L Wu C Zhang ZJ Samoilova M Eubanks J. Hippocampal seizures alter the expression of the pannexin and connexin transcriptome. J Neurochem. (2010) 112:92–102. 10.1111/j.1471-4159.2009.06431.x 19840216

[243] Jiang T Long H Ma Y Long L Li Y Li F. Altered expression of pannexin proteins in patients with temporal lobe epilepsy. Mol Med Rep. (2013) 8:1801–6. 10.3892/mmr.2013.1739 24146091

[244] Jimenez-Pacheco A Mesuret G Sanz-Rodriguez A Tanaka K Mooney C Conroy R. Increased neocortical expression of the P2X7 receptor after status epilepticus and anticonvulsant effect of P2X7 receptor antagonist A-438079. Epilepsia. (2013) 54:1551–61. 10.1111/epi.12257 23808395

[245] Grygorowicz T Wełniak-Kamińska M Struzyńska L. Early P2X7R-related astrogliosis in autoimmune encephalomyelitis. Mol Cell Neurosci. (2016) 74:1–9. 10.1016/j.mcn.2016.02.003 26921791

[246] Evanko DS Zhang Q Zorec R Haydon PG. Defining pathways of loss and secretion of chemical messengers from astrocytes. Glia. (2004) 47:233–40. 10.1002/glia.20050 15252812

[247] Retamal MA Froger N Palacios-Prado N Ezan P Sáez PJ Sáez JC. Cx43 hemichannels and gap junction channels in astrocytes are regulated oppositely by proinflammatory cytokines released from activated microglia. J Neurosci. (2007) 27:13781–92. 10.1523/JNEUROSCI.2042-07.2007 18077690 PMC6673621

[248] Bennet MV Garré JM Orellana JA Bukauskas FF Nedergaard M Sáez JC. Connexin and pannexin hemichannels in inflammatory responses of glia and neurons. Brain Res. (2012) 3:3–15. 10.1161/CIRCULATIONAHA.110.956839 22975435 PMC3627726

[249] Orellana JA Von Bernhardi R Giaume C Sáez JC. Glial hemichannels and their involvement in aging and neurodegenerative diseases. Rev Neurosci. (2012) 23:163–77. 10.1515/revneuro-2011-0065 22499675

[250] Medina-Ceja L Salazar-Sánchez JC Ortega-Ibarra J Morales-Villagrán A. Connexins-based hemichannels/channels and their relationship with inflammation, seizures and epilepsy. Int J Mol Sci. (2019) 20:1–17. 10.3390/ijms20235976 31783599 PMC6929063

[251] Santiago MF Veliskova J Patel NK Lutz SE Caille D Charollais A. Targeting pannexin1 improves seizure outcome. PLoS ONE. (2011) 6:e25178. 10.1371/journal.pone.0025178 21949881 PMC3175002

[252] Dossi E Blauwblomme T Moulard J Chever O Vasile F Guinard E. Pannexin-1 channels contribute to seizure generation in human epileptic brain tissue and in a mouse model of epilepsy. Sci Transl Med. (2018) 10:1–14. 10.1126/scitranslmed.aar3796 29848662

[253] Engel T Gomez-Villafuertes R Tanaka K Mesuret G Sanz-Rodriguez A Garcia-Huerta P. Seizure suppression and neuroprotection by targeting the purinergic P2X7 receptor during status epilepticus in mice. FASEB J. (2012) 26:1616–28. 10.1096/fj.11-196089 22198387

[254] Amhaoul H Ali I Mola M Van Eetveldt A Szewczyk K Missault S. P2X7 receptor antagonism reduces the severity of spontaneous seizures in a chronic model of temporal lobe epilepsy. Neuropharmacology. (2016) 105:175–85. 10.1016/j.neuropharm.2016.01.018 26775823

[255] Lord B Aluisio L Shoblock JR Neff RA Varlinskaya EI Ceusters M. Pharmacology of a novel central nervous system-penetrant P2X7 antagonist JNJ-42253432. J Pharmacol Exp Ther. (2014) 351:628–41. 10.1124/jpet.114.218487 25271258

[256] Engel T Alves M Sheedy C Henshall DC. ATPergic signalling during seizures and epilepsy. Neuropharmacology. (2016) 104:140–53. 10.1016/j.neuropharm.2015.11.001 26549853

[257] Fischer W Franke H Krügel U Müller H Dinkel K. Critical evaluation of P2X7 receptor antagonists in selected seizure models. PLoS ONE. (2016) 11:e0156468. 10.1371/journal.pone.0156468 27281030 PMC4900628

[258] Riquelme J Wellmann M Sotomayor-Zárate R Bonansco C. Gliotransmission: a novel target for the development of antiseizure drugs. Neuroscientist. (2020) 26:293–309. 10.1177/1073858420901474 31976817

[259] Choi DW. Glutamate receptors and the induction of excitotoxic neuronal death. Prog Brain Res. (1994) 100:47–51. 10.1016/S0079-6123(08)60767-0 7938533

[260] Perez EL Lauritzen F Wang Y Lee TSW Kang D Zaveri HP. Evidence for astrocytes as a potential source of the glutamate excess in temporal lobe epilepsy. Neurobiol Dis. (2012) 47:331–7. 10.1016/j.nbd.2012.05.010 22659305 PMC3392431

[261] Syková E Nicholson C. Diffusion in brain extracellular space. Physiol Rev. (2008) 88:1277–340. 10.1152/physrev.00027.2007 18923183 PMC2785730

[262] Sosunov AA Wu X Tsankova NM Guilfoyle E McKhann GM Goldman JE. Phenotypic heterogeneity and plasticity of isocortical and hippocampal astrocytes in the human brain. J Neurosci. (2014) 34:2285–98. 10.1523/JNEUROSCI.4037-13.2014 24501367 PMC3913872

[263] Zielinska M Dabrowska K Hadera MG Sonnewald U Albrecht J. System N transporters are critical for glutamine release and modulate metabolic fluxes of glucose and acetate in cultured cortical astrocytes: changes induced by ammonia. J Neurochem. (2016) 136:329–38. 10.1111/jnc.13376 26442479

[264] Parpura V Schousboe A Verkhratsky A. Glutamate and ATP at the interface of metabolism and signaling in the brain. Adv Neurobiol. (2014) 11:11–30. 10.1007/978-3-319-08894-5 25236721

[265] Cavus I Kasoff WS Cassaday MP Jacob R Gueorguieva R Sherwin RS. Extracellular metabolites in the cortex and hippocampus of epileptic patients. Ann Neurol. (2005) 57:226–35. 10.1002/ana.20380 15668975

[266] Cavus I Pan JW Hetherington HP Abi-Saab W Zaveri HP Vives KP. Decreased hippocampal volume on MRI is associated with increased extracellular glutamate in epilepsy patients. Epilepsia. (2008) 49:1358–66. 10.1111/j.1528-1167.2008.01603.x 18410365

[267] Ueda Y Doi T Tokumaru J Yokoyama H Nakajima A Mitsuyama Y. Collapse of extracellular glutamate regulation during epileptogenesis: down-regulation and functional failure of glutamate transporter function in rats with chronic seizures induced by kainic acid. J Neurochem. (2001) 76:892–900. 10.1046/j.1471-4159.2001.00087.x 11158261

[268] Gorter JA Van Vliet EA Proper EA De Graan PNE Ghijsen WEJM Lopes Da Silva FH. Glutamate transporters alterations in the reorganizing dentate gyrus are associated with progressive seizure activity in chronic epileptic rats. J Comp Neurol. (2002) 442:365–77. 10.1002/cne.10101 11793340

[269] Lopes MW Soares FMS De Mello N Nunes JC Cajado AG De Brito D. Time-dependent modulation of AMPA receptor phosphorylation and mRNA expression of NMDA receptors and glial glutamate transporters in the rat hippocampus and cerebral cortex in a pilocarpine model of epilepsy. Exp Brain Res. (2013) 226:153–63. 10.1007/s00221-013-3421-8 23392471

[270] Samuelsson C Kumlien E Flink R Lindholm D Ronne-Engström E. Decreased cortical levels of astrocytic glutamate transport protein GLT- 1 in a rat model of posttraumatic epilepsy. Neurosci Lett. (2000) 289:185–8. 10.1016/S0304-3940(00)01284-2 10961660

[271] Watanabe T Morimoto K Hirao T Suwaki H Watase K Tanaka K. Amygdala-kindled and pentylenetetrazole-induced seizures in glutamate transporter GLAST-deficient mice. Brain Res. (1999) 845:92–6. 10.1016/S0006-8993(99)01945-9 10529447

[272] Tanaka K Watase K Manabe T Yamada K Watanabe M Takahashi K. Epilepsy and exacerbation of brain injury in mice lacking the glutamate transporter GLT-1. Science. (1997) 276:1699–702. 10.1126/science.276.5319.1699 9180080

[273] Sepkuty JP Cohen AS Eccles C Rafiq A Behar K Ganel R. A neuronal glutamate transporter contributes to neurotransmitter GABA synthesis and epilepsy. J Neurosci. (2002) 22:6372–9. 10.1523/JNEUROSCI.22-15-06372.2002 12151515 PMC2483507

[274] Sarac S Afzal ÃS Broholm ÃH Madsen FF. Excitatory amino acid transporters EAAT-1 and EAAT-2 in temporal lobe and hippocampus in intractable temporal lobe epilepsy. APMIS. (2009) 291–301. 10.1111/j.1600-0463.2009.02443.x 19338517

[275] Zeng L-H Bero AW Zhang B Holtzman DM Wong M. Modulation of astrocyte glutamate transporters decreases seizures in a mouse model of tuberous sclerosis complex. Neurobiol Dis. (2010) 37:764–71. 10.1016/j.nbd.2009.12.020 20045054 PMC2823985

[276] Susarla BTS Robinson MB. Internalization and degradation of the glutamate transporter GLT-1 in response to phorbol ester. Neurochem Int. (2008) 52:709–22. 10.1016/j.neuint.2007.08.020 17919781 PMC2292111

[277] Sha L Wang X Li J Shi X Wu L Shen Y. Pharmacologic inhibition of Hsp90 to prevent GLT-1 degradation as an effective therapy for epilepsy. J Exp Med. (2017) 214:547–63. 10.1084/jem.20160667 28028152 PMC5294855

[278] Eid T Thomas MJ Spencer DD Rundén-Pran E Lai JCK Malthankar GV. Loss of glutamine synthetase in the human epileptogenic hippocampus: possible mechanism for raised extracellular glutamate in mesial temporal lobe epilepsy. Lancet. (2004) 363:28–37. 10.1016/S0140-6736(03)15166-5 14723991

[279] Van Der Hel WS Notenboom RGE Bos IWM Van Rijen PC Van Veelen CWM De Graan PNE. Reduced glutamine synthetase in hippocampal areas with neuron loss in temporal lobe epilepsy. Neurology. (2005) 64:326–33. 10.1212/01.WNL.0000149636.44660.99 15668432

[280] Ortinski PI Dong J Mungenast A Yue C Takano H Watson DJ. Selective induction of astrocytic gliosis generates deficits in neuronal inhibition. Nat Publ Gr. (2010) 13:584–91. 10.1038/nn.2535 20418874 PMC3225960

[281] Zou J Wang YX Dou FF Lü HZ Ma ZW Lu PH. Glutamine synthetase down-regulation reduces astrocyte protection against glutamate excitotoxicity to neurons. Neurochem Int. (2010) 56:577–84. 10.1016/j.neuint.2009.12.021 20064572 PMC2831119

[282] Eid T Lee TSW Patrylo P Zaveri HP. Astrocytes and glutamine synthetase in epileptogenesis. J Neurosci Res. (2019) 97:1345–62. 10.1002/jnr.24267 30022509 PMC6338538

[283] Khazipov R. GABAergic synchronization in epilepsy. Cold Spring Harb Perspect Med. (2016) 6:1–13. 10.1101/cshperspect.a022764 26747834 PMC4743071

[284] Navazio F Gerritsen T Wright GJ. Relationship of ammonia intoxication to convulsions and coma in rats. J Neurochem. (1961) 8:146–51. 10.1111/j.1471-4159.1961.tb13536.x 14478630

[285] Papageorgiou IE Valous NA Lahrmann B Janova H Klaft ZJ Koch A. Astrocytic glutamine synthetase is expressed in the neuronal somatic layers and down-regulated proportionally to neuronal loss in the human epileptic hippocampus. Glia. (2018) 66:920–33. 10.1002/glia.23292 29350438

[286] Kritis AA Stamoula EG Paniskaki KA Vavilis TD. Researching glutamate – induced cytotoxicity in different cell lines: a comparative/collective analysis/study. Front Cell Neurosci. (2015) 9:91. 10.3389/fncel.2015.00091 25852482 PMC4362409

[287] Robel S Buckingham XSC Boni XJL Campbell SL Danbolt NC Riedemann T. Reactive astrogliosis causes the development of spontaneous seizures. J Neurosci. (2015) 35:3330–45. 10.1523/JNEUROSCI.1574-14.2015 25716834 PMC4339349

[288] Martineau M Parpura V Mothet JP. Cell-type specific mechanisms of D-serine uptake and release in the brain. Front Synaptic Neurosci. (2014) 6:12. 10.3389/fnsyn.2014.00012 24910611 PMC4039169

[289] Scianni M Antonilli L Chece G Cristalli G Di Castro MA Limatola C. Fractalkine (CX3CL1) enhances hippocampal N-methyl-d-aspartate receptor (NMDAR) function via d-serine and adenosine receptor type A2 (A2AR) activity. J Neuroinflamm. (2013) 10:1–15. 10.1186/1742-2094-10-108 23981568 PMC3765929

[290] Martineau M Galli T Baux G Mothet JP. Confocal imaging and tracking of the exocytotic routes for D-serine-mediated gliotransmission. Glia. (2008) 56:1271–84. 10.1002/glia.20696 18615566

[291] Zhuang Z Yang B Theus MH Sick JT Bethea JR Sick TJ. EphrinBs regulate D-serine synthesis and release in astrocytes. J Neurosci. (2010) 30:16015–24. 10.1523/JNEUROSCI.0481-10.2010 21106840 PMC3073557

[292] Schell MJ Molliver ME Snyder SH. D-serine, an endogenous synaptic modulator: localization to astrocytes and glutamate-stimulated release. Proc Natl Acad Sci USA. (1995) 92:3948–52. 10.1073/pnas.92.9.3948 7732010 PMC42079

[293] Diniz LP Almeida JC Tortelli V Lopes CV Setti-Perdigão P Stipursky J. Astrocyte-induced synaptogenesis is mediated by transforming growth factor β signaling through modulation of d-serine levels in cerebral cortex neurons. J Biol Chem. (2012) 287:41432–45. 10.1074/jbc.M112.380824 23055518 PMC3510841

[294] Takata N Mishima T Hisatsune C Nagai T Ebisui E Mikoshiba K. Astrocyte calcium signaling transforms cholinergic modulation to cortical plasticity *in vivo*. J Neurosci. (2011) 31:18155–65. 10.1523/JNEUROSCI.5289-11.2011 22159127 PMC6634158

[295] López-Hidalgo M Salgado-Puga K Alvarado-Martínez R Medina AC Prado-Alcalá RA García-Colunga J. Nicotine uses neuron-glia communication to enhance hippocampal synaptic transmission and long-term memory. PLoS ONE. (2012) 7:e49998. 10.1371/journal.pone.0049998 23185511 PMC3503711

[296] Wolosker H Balu DT Coyle JT. The rise and fall of the D-serine-mediated gliotransmission hypothesis. Trends Neurosci. (2016) 39:712–21. 10.1016/j.tins.2016.09.007 27742076 PMC5113294

[297] Ehmsen JT Ma TM Sason H Rosenberg D Ogo T Furuya S. D-serine in glia and neurons derives from 3-phosphoglycerate dehydrogenase. J Neurosci. (2013) 33:12464–9. 10.1523/JNEUROSCI.4914-12.2013 23884950 PMC3721849

[298] Wolosker H Radzishevsky I. The serine shuttle between glia and neurons: implications for neurotransmission and neurodegeneration. Biochem Soc Trans. (2013) 41:1546–50. 10.1042/BST20130220 24256252

[299] Perez EJ Tapanes SA Loris ZB Balu DT Sick TJ Coyle JT. Enhanced astrocytic d-serine underlies synaptic damage after traumatic brain injury. J Clin Invest. (2017) 127:3114–25. 10.1172/JCI92300 28714867 PMC5531405

[300] Ma T Wu Y Chen B Zhang W Jin L Shen C. D-Serine contributes to seizure development via ERK signaling. Front Neurosci. (2019) 13:254. 10.3389/fnins.2019.00254 30971878 PMC6443828

[301] Losi G Cammarota M Carmignoto G. The role of astroglia in the epileptic brain. Front Pharmacol. (2012) 3:132. 10.3389/fphar.2012.00132 22807916 PMC3395023

[302] Wang CM Chang YY Kuo JS Sun SH. Activation of P2x7 receptors induced [3H]GABA release from the RBA-2 type-2 astrocyte cell line through a Cl-/HCO3- - dependent mechanism. Glia. (2002) 37:8–18. 10.1002/glia.10004 11746779

[303] Torres A Wang F Xu Q Fujita T Dobrowolski R Willecke K. Extracellular Ca^2+^ acts as a mediator of communication from neurons to glia. Sci Signal. (2012) 5:ra28. 10.1126/scisignal.2002160 22275221 PMC3548660

[304] Kumaria A Tolias CM Burnstock G. ATP signalling in epilepsy. Purinergic Signal. (2008) 4:339–46. 10.1007/s11302-008-9115-1 18568425 PMC2583203

[305] Gordon GRJ Iremonger KJ Kantevari S Ellis-Davies GCR MacVicar BA Bains JS. Astrocyte-mediated distributed plasticity at hypothalamic glutamate synapses. Neuron. (2009) 64:391–403. 10.1016/j.neuron.2009.10.021 19914187 PMC4107870

[306] Scemes E Velíšek L Velíšková J. Astrocyte and neuronal pannexin1 contribute distinctly to seizures. ASN Neuro. (2019) 11:1–12. 10.1177/1759091419833502 30862176 PMC6415468

[307] Aquilino MS Whyte-Fagundes P Zoidl G Carlen PL. Pannexin-1 channels in epilepsy. Neurosci Lett. (2019) 695:71–5. 10.1016/j.neulet.2017.09.004 28886985

[308] Rudolphi KA Schubert P. Modulation of neuronal and glial cell function by adenosine and neuroprotection in vascular dementia. Behav Brain Res. (1997) 83:123–8. 10.1016/s0166-4328(97)86055-x 9062670

[309] Calker D Van Biber K. The role of glial adenosine receptors in neural resilience and the neurobiology of mood disorders. Neurochem Res. (2005) 30:1205–17. 10.1007/s11064-005-8792-1 16341582

[310] Boison D Astrogliosis and adenosine kinase: a glial basis of epilepsy. Futur Neurol. (2008) 3:221–4. 10.2217/14796708.3.3.221

[311] Boison D. Adenosinergic signaling in epilepsy. Neuropharmacology. (2016) 104:131–9. 10.1016/j.neuropharm.2015.08.046 26341819 PMC4775444

[312] Lopes LV Cunha RA Ribeiro JA. Cross talk between A 1 and A 2A adenosine receptors in the hippocampus and cortex of young adult and old rats. J Neurophysiol. (1999) 82:3196–203. 10.1152/jn.1999.82.6.3196 10601453

[313] Ciruela F Casado V Rodrigues RJ Luja R Burguen J Canals M Presynaptic control of striatal glutamatergic neurotransmission by adenosine A 1 – A 2A receptor heteromers. J Neurosci. (2006) 26:2080–7. 10.1523/JNEUROSCI.3574-05.2006 16481441 PMC6674939

[314] Glass M Faull RLM Jansen K Walker EB Synek BJL Dragunow M. Loss of A1 adenosine receptors in human temporal lobe epilepsy. Brain Res. (1996) 710:56–68. 10.1016/0006-8993(95)01313-x 8963679

[315] Ekonomou A Sperk G Kostopoulos G Angelatou F. Reduction of A1 adenosine receptors in rat hippocampus after kainic acid-induced limbic seizures. Neurosci Lett. (2000) 284:49–52. 10.1016/s0304-3940(00)00954-x 10771159

[316] Barros-Barbosa AR Ferreirinha F Oliveira A Mendes M Lobo MG Santos A. Adenosine A2A receptor and ecto-5′-nucleotidase-CD73 are upregulated in hippocampal astrocytes of human MTLE. Purinergic Signal. (2016) 12:719–34. 10.1007/s11302-016-9535-2 27650530 PMC5124012

[317] Hindley S Herman MAR Rathbone MP Stimulation of reactive astrogliosis *in vivo* by extracellular adenosine diphosphate or an adenosine A2 receptor agonist. J Neurosci Res. (1994) 38:399–406. 10.1002/jnr.490380405 7932872

[318] Popoli P Blum D Martire A Ledent C Ceruti S Abbracchio MP. Functions, dysfunctions and possible therapeutic relevance of adenosine A 2A receptors in Huntington' s disease. Prog Neurobiol. (2007) 81:331–48. 10.1016/j.pneurobio.2006.12.005 17303312

[319] Ribeiro JA Dio MJ Sebastia AM. Influence of age on BDNF modulation of hippocampal synaptic transmission : interplay with adenosine A 2A receptors. Hippocampus. (2007) 17:577–85. 10.1002/hipo 17421024

[320] Ke R Xiong J Liu Y Ye Z. Adenosine A2a receptor induced gliosis via Akt/NF-kB pathway *in vitro*. Neurosci Res. (2009) 65:280–5. 10.1016/j.neures.2009.08.002 19666061

[321] Erion MD Ugarkar BG Dare J Catellino AJ Fujitaki JM Dixon R Design, synthesis and anticonvulsant activity of the potent adenosine kinase inhibitor GP3269. Nucleic Acids. (1998) 16:1013–21. 10.1080/07328319708006124

[322] Ugarkar BG DaRe JM Kopcho JJ Browne CE Schanzer JM Wiesner JB. Adenosine kinase inhibitors. 1. Synthesis, enzyme inhibition, and antiseizure activity of 5-iodotubercidin analogues. J Med Chem. (2000) 43:2883–93. 10.1021/jm000024g 10956196

[323] Ugarkar BG Castellino AJ DaRe JM Kopcho JJ Wiesner JB Schanzer JM. Adenosine kinase inhibitors. 2. Synthesis, enzyme inhibition, and antiseizure activity of diaryltubercidin analogues. J Med Chem. (2000) 43:2894–905. 10.1021/jm0000259 10956197

[324] Zhang G Franklin PH Murray TF. Manipulation of endogenous adenosine in the rat prepiriform cortex modulates seizure susceptibility. J Pharmacol Exp Ther. (1993) 264:1415–24. 8450475

[325] McGaraughty S Cowart M Jarvis M Berman R. Anticonvulsant and antinociceptive actions of novel adenosine kinase inhibitors. Curr Top Med Chem. (2005) 5:43–58. 10.2174/1568026053386845 15638777

[326] Köse M Schiedel AC Bauer AA Poschenrieder H Burbiel JC Akkinepally RR. Focused screening to identify new adenosine kinase inhibitors. Bioorganic Med Chem. (2016) 24:5127–33. 10.1016/j.bmc.2016.08.026 27595538

[327] Arciénega II Brunet JF Bloch J Badaut J. Cell locations for AQP1, AQP4 and 9 in the non-human primate brain. Neuroscience. (2010) 167:1103–14. 10.1016/j.neuroscience.2010.02.059 20226845

[328] Eid T Lee TW Thomas MJ Amiry-Moghaddam M Bjørnsen LP Spencer DD. Loss of perivascular aquaporin 4 may underlie deficient water and K^+^ homeostasis in the human epileptogenic hippocampus. Proc Natl Acad Sci USA. (2005) 102:1193–8. 10.1073/pnas.0409308102 15657133 PMC545857

[329] Sheilabi MA Bhattacharyya D Kitchen P Conner AC Salman MM Bill RM. Transcriptome analysis suggests a role for the differential expression of cerebral aquaporins and the MAPK signalling pathway in human temporal lobe epilepsy. Eur J Neurosci. (2017) 46:2121–32. 10.1111/ejn.13652 28715131

[330] Lee TS Eid T Mane S Kim JH Spencer DD Ottersen OP. Aquaporin-4 is increased in the sclerotic hippocampus in human temporal lobe epilepsy. Acta Neuropathol. (2004) 108:493–502. 10.1007/s00401-004-0910-7 15517312

[331] Binder DK Auser CS Words KEY. Functional changes in astroglial cells in epilepsy. Glia. (2006) 368:358–68. 10.1002/glia.20394 16886201

[332] Coulter DA Steinha C. Role of astrocytes in epilepsy. Cold Spring Harb Perspect Med. (2015) 5:649–71. 10.1101/cshperspect.a022434 25732035 PMC4355248

[333] Li X Zhou J Chen Z Chen S Zhu F Liemin Z. Long-term expressional changes of Na^+^-K^+^-Cl^−^ co-transporter NKCC1 and KCC2 in CA1 region of hippo following pilo epilepsy. Brain Res. (2008) 141–6. 10.1016/j.brainres.2008.04.047 18550034

[334] Brandt C Nozadze M Heuchert N Rattka M Löscher W. Disease-modifying effects of phenobarbital and the NKCC1 inhibitor bumetanide in the pilocarpine model of temporal lobe epilepsy. J Neurosci. (2010) 30:8602–12. 10.1523/JNEUROSCI.0633-10.2010 20573906 PMC6634618

[335] Otalora LFP Hernandez EF Arshadmansab MF Sebastian F Willis M Ermolinsky B. Downregulation of BK channel expression in the pilocarpine model of temporal lobe epilepsy. Brain Res. (2009) 1200:116–31. 10.1016/j.brainres.2008.01.017 18295190 PMC2346580

[336] Yeo JKS Jin H. Changes in TWIK-related acid sensitive K^+^−1 and−3 channel expressions from neurons to glia in the hippocampus of temporal lobe epilepsy patients and experimental animal model. Neurochem Res. (2011) 36:2155–68. 10.1007/s11064-011-0540-0 21710317

[337] Nagao Y Harada Y Mukai T Shimizu S Okuda A Fujimoto M. Expressional analysis of the astrocytic Kir4. 1 channel in a pilocarpine-induced temporal lobe epilepsy model. Front Cell Neurosci. (2013) 7:104. 10.3389/fncel.2013.00104 23922547 PMC3734973

[338] Kang SJ Cho S Park K Yi J Yoo SJ Shin KS. Expression of Kir2. 1 channels in astrocytes under pathophysiological conditions. Mol Cells. (2008) 25:124–30. 18319624

[339] Kim D Kim J Kwak S Won MH Kang T. Seizure activity affects neuroglial Kv1 channel immunoreactivities in the gerbil hippocampus. Brain Res. (2007) 1151:172–87. 10.1016/j.brainres.2007.03.017 17397809

[340] Grisar T Guillaume D Delgado-Escueta AV. Contribution of Na^+^, K^+^-ATPase to focal epilepsy: a brief review. Epilepsy Res. (1992) 12:141–9. 10.1016/0920-1211(92)90034-q 1327744

[341] Palma E Amici M Sobrero F Spinelli G Angelantonio S Di Ragozzino D. Anomalous levels of Cl- transporters in the hippocampal subiculum from temporal lobe epilepsy patients make GABA excitatory. Proc Natl Acad Sci USA. (2006) 103:8465–8. 10.1073/pnas.0602979103 16709666 PMC1482515

[342] Ketelaars SOM Gorter JA Aronica E Wadman WJ. Calcium extrusion protein expression in the hippocampal formation of chronic epileptic rats after kainate-induced status epilepticus. Epilepsia. (2004) 45:1189–201. 10.1111/j.0013-9580.2004.03304.x 15461673

[343] Lin YW Hsieh CL Auricular electroacupuncture reduced inflammation-related epilepsy accompanied by altered trpa1, ppkc ppkc ε, and perk1/2 signaling pathways in kainic acid-treated rats. Mediators Inflamm. (2014) 2014:1–9. 10.1155/2014/493480 PMC4131505 25147437

[344] Tai C Hines DJ Choi HB MacVicar BA. Plasma membrane insertion of TRPC5 channels contributes to the cholinergic plateau potential in hippocampal CA1 pyramidal neurons. Hippocampus. (2011) 21:958–67. 10.1002/hipo.20807 20865744

[345] Xu GZ Shu H Yue HY Zheng DH Guo W Yang H. Increased expression of TRPC5 in cortical lesions of the focal cortical dysplasia. J Mol Neurosci. (2014) 55:561–9. 10.1007/s12031-014-0390-8 25085710

[346] Zeng C Zhou P Jiang T. Upregulation and diverse roles of TRPC3 and TRPC6 in synaptic reorganization of the mossy fiber pathway in temporal lobe epilepsy. Mol Neurobiol. (2015) 52:562–72. 10.1007/s12035-014-8871-x 25213992

[347] Sun F Guo W Zheng D Zhang C Increased expression of TRPV1 in the cortex and hippocampus from patients with mesial temporal lobe epilepsy. J Mol Neurosci. (2013) 1:182–93. 10.1007/s12031-012-9878-2 22936245

[348] Seifert G Schröder W Hinterkeuser S Schumacher T Schramm J Steinhäuser C. Changes in flip/flop splicing of astroglial AMPA receptors in human temporal lobe epilepsy. Epilepsia. (2002) 43:162–7. 10.1046/j.1528-1157.43.s.5.10.x 12121314

[349] Seifert G Hu K Schramm J Steinha C. Enhanced relative expression of glutamate receptor 1 flip AMPA receptor subunits in hippocampal astrocytes of epilepsy patients with ammon' s horn sclerosis. J Neurosci. (2004) 24:1996–2003. 10.1523/JNEUROSCI.3904-03.2004 14985442 PMC6730392

[350] Naylor DE Lio H Niquet J Wasterlain CG. Rapid surface accumulation of NMDA receptors increases glutamatergic excitation during status epilepticus. Neurobiol Dis. (2013) 54:225–38. 10.1016/j.nbd.2012.12.015 23313318 PMC5444936

[351] Simard M Nedergaard M. The neurobiology of glia in the context of water and ion homeostasis. Neuroscience. (2004) 129:877–96. 10.1016/j.neuroscience.2004.09.053 15561405

[352] Ke C Poon WS Ng HK Lai FMM Tang NLS Pang JCS. Impact of experimental acute hyponatremia on severe traumatic brain injury in rats : influences on injuries, permeability of blood – brain. Exp Neurol. (2002) 206:194–206. 10.1006/exnr.2002.8037 12504879

[353] Pasantes-Morales H Franco R Ordaz B Ochoa LD. Mechanisms counteracting swelling in brain cells during hyponatremia. Arch Med Res. (2002) 33:237–44. 10.1016/s0188-4409(02)00353-3 12031627

[354] Murphy TR Binder DK Fiacco TA. Turning down the volume: astrocyte volume change in the generation and termination of epileptic seizures. Neurobiol Dis. (2017) 104:24–32. 10.1016/j.nbd.2017.04.016 28438505 PMC5522738

[355] Wang F Qi X Zhang J Huang J. Astrocytic modulation of potassium under seizures. Neural Regen Res. (2020) 15:980–7. 10.4103/1673-5374.270295 31823867 PMC7034283

[356] Abdullaev IF Rudkouskaya A Schools GP Kimelberg HK Mongin AA. Pharmacological comparison of swelling-activated excitatory amino acid release and Cl- currents in cultured rat astrocytes. J Physiol. (2006) 572:677–89. 10.1113/jphysiol.2005.103820 16527858 PMC1780004

[357] Haskew-Layton RE Rudkouskaya A Jin Y Feustel PJ Kimelberg HK Mongin AA. Two distinct modes of hypoosmotic medium-induced release of excitatory amino acids and taurine in the rat brain *in vivo*. PLoS ONE. (2008) 3:e3543. 10.1371/journal.pone.0003543 18958155 PMC2568819

[358] Seifert G Henneberger C Steinhäuser C. Diversity of astrocyte potassium channels : an update. Brain Res Bull. (2018) 136:26–36. 10.1016/j.brainresbull.2016.12.002 27965079

[359] Day RE Kitchen P Owen DS Bland C Marshall L Conner AC. Human aquaporins : regulators of transcellular water flow. BBA Gen Subj. (2014) 1840:1492–506. 10.1016/j.bbagen.2013.09.033 24090884

[360] Macaulay N Hamann S Zeuthen T. Water transport in the brain: role of cotransporters. Neuroscience. (2004) 129:1031–44. 10.1016/j.neuroscience.2004.06.045 15561418

[361] Papadopoulos MC Verkman AS. Aquaporin water channels in the nervous system. Nat Rev Neurosci. (2014) 14:265–77. 10.1038/nrn3468 23481483 PMC3732112

[362] Wetherington J Serrano G Dingledine R. Astrocytes in the epileptic brain. Neuron. (2008) 58:168–78. 10.1016/j.neuron.2008.04.002 18439402 PMC4124883

[363] Frigeri A Nicchia GP Nico B Quondamatteo F Herken R RONCALI L. Aquaporin-4 deficiency in skeletal muscle and brain of dystrophic mdx mice. FASEB J. (2001) 15:90–8. 10.1096/fj.00-0260com 11149896

[364] Wertz K Stødkilde-Jørgensen H Sulyok E Vajda Z Pedersen M Fu E. Delayed onset of brain edema and mislocalization of aquaporin-4 in dystrophin-null transgenic mice. Proc Natl Acad Sci USA. (2002) 99:13131–6. 10.1073/pnas.192457099 12232046 PMC130598

[365] Marchi N Granata T Ghosh C Janigro D. Blood–brain barrier dysfunction and epilepsy: pathophysiologic role and therapeutic approaches. Epilepsia. (2012) 53:1877–86. 10.1111/j.1528-1167.2012.03637.x 22905812 PMC4842020

[366] Lee DJ Hsu MS Seldin MM Arellano JL Binder DK. Decreased expression of the glial water channel aquaporin-4 in the intrahippocampal kainic acid model of epileptogenesis. Exp Neurol. (2013) 235:246–55. 10.1016/j.expneurol.2012.02.002 22361023 PMC3334411

[367] Alvestad S Hammer J Hellstrøm E Skare Ø Sonnewald U Amiry-Moghaddam M. Mislocalization of AQP4 precedes chronic seizures in the kainate model of temporal lobe epilepsy. Epilepsy Res. (2013) 105:30–41. 10.1016/j.eplepsyres.2013.01.006 23357720

[368] Han X Huang Q Liu L Sha X Hu B Liu H. Changes in the expression of AQP4 and AQP9 in the hippocampus following eclampsia-like seizure. Int J Mol Sci. (2018) 19:1–12. 10.3390/ijms19010300 29351212 PMC5796245

[369] Kim JE Ryu HJ Yeo SI Seo CH Lee BC Choi IG. Differential expressions of aquaporin subtypes in astroglia in the hippocampus of chronic epileptic rats. Neuroscience. (2009) 163:781–9. 10.1016/j.neuroscience.2009.07.028 19619613

[370] Liu H Yang M Qiu G Zhuo F Yu W Sun S. Aquaporin 9 in rat brain after severe traumatic brain injury. Arq Neuropsiquiatr. (2012) 70:214–20. 10.1590/s0004-282x2012000300012 22392116

[371] Shenaq M Kassem H Peng C Schafer S Ding JY Fredrickson V. Neuronal damage and functional deficits are ameliorated by inhibition of aquaporin and HIF1α after traumatic brain injury (TBI). J Neurol Sci. (2012) 323:134–40. 10.1016/j.jns.2012.08.036 23040263

[372] Saadoun S Papadopoulos MC Watanabe H Yan D Manley GT Verkman AS. Involvement of aquaporin-4 in astroglial cell migration and glial scar formation. J Cell Sci. (2005) 118:5691–8. 10.1242/jcs.02680 16303850

[373] Hibino H Fujita A Iwai K Yamada M Kurachi Y. Differential assembly of inwardly rectifying K^+^ channel subunits, Kir4.1 and Kir5.1, in brain astrocytes. J Biol Chem. (2004) 279:44065–73. 10.1074/jbc.M405985200 15310750

[374] Kucheryavykh YV Kucheryavykh LY Nichols CG Maldonado HM Baksi K Reichenback A Downregulation of Kir4.1 inward rectifying potassium channel subunits by RNAi impairs potassium transfer and glutamate uptake by cultured cortical astrocytes. Glia. (2007) 281:274–81. 10.1002/glia.20455 17091490

[375] Li X Potts EA Chen M Perilla PR Bredt DS Simard JM. Inward rectifier K^+^ channel Kir2.3 (IRK3) in reactive astrocytes from adult rat brain. Glia. (2000) 192:181–92. 10.1002/1098-1136(200008)31:2<181::aid-glia90>3.0.co;2-8 10878604

[376] Thomzig A Wenzel M Karschin C Eaton MJ Skatchkov SN Karschin A. Kir6.1 is the principal pore-forming subunit of astrocyte but not neuronal plasma membrane K-ATP channels. Mol Cell Neurosci. (2001) 690:671–90. 10.1006/mcne.2001.1048 11749042

[377] Connors NC Adams ME Froehner SC Kofuji P. The potassium channel Kir4. 1 associates with the dystrophin- glycoprotein complex via alfa-syntrophin in glia. J Biol Chem. (2004) 279:28387–92. 10.1074/jbc.M402604200 15102837

[378] Bragg AD Amiry-Moghaddam M Ottersen OLEP Adams ME Assembly of a perivascular astrocyte protein scaffold at the mammalian blood – brain barrier is dependent on a -syntrophin. Glia. (2006) 890:879–90. 10.1002/glia.20347 16609960

[379] Hubbard JA Hsu MS Seldin MM Binder DK. Expression of the astrocyte water channel aquaporin-4 in the mouse brain. ASN Neuro. (2015) 7:1759091415605486. 10.1177/1759091415605486 26489685 PMC4623559

[380] Jin B Zhang H Binder DK Verkman AS. Aquaporin-4 – dependent K^+^ and water transport modeled in brain extracellular space following neuroexcitation. J Gen Physiol. (2013) 141:119–32. 10.1085/jgp.201210883 23277478 PMC3536523

[381] Soe R Macaulay N Arne D. Modulation of Kir4. 1 and Kir4. 1 – Kir5. 1 channels by small changes in cell volume. Neurosci Lett. (2009) 457:80–84. 10.1016/j.neulet.2009.04.010 19429167

[382] Bringmann A Francke M Pannicke T Biedermann B Kodal H Faude F. Role of *Glia*l K^+^ channels in ontogeny and gliosis: a hypothesis based upon studies on müller cells. Glia. (2000) 44:35–44. 10.1002/(sici)1098-1136(20000101)29:1<35::aid-glia4>3.0.co;2-a 10594921

[383] Olsen ML Campbell SC Mcferrin MB Floyd CL Sontheimer H. Spinal cord injury causes a wide-spread, persis- tent loss of Kir4.1 and glutamate transporter 1: benefit of 17 beta-oestradiol treatment. Brain. (2010) 133:1013–25. 10.1093/brain/awq049 20375134 PMC2850584

[384] Frigerio F Frasca A Weissberg I Parrella S Friedman A Vezzani A. Long-lasting pro-ictogenic effects induced *in vivo* by rat brain exposure to serum albumin in the absence of concomitant pathology. Epilepsia. (2012) 53:1887–97. 10.1111/j.1528-1167.2012.03666.x 22984896 PMC3651831

[385] Stewart TH Eastman CL Groblewski PA Fender JS Verley DR Cook DG. Chronic dysfunction of astrocytic inwardly rectifying K^+^ channels specific to the neocortical epileptic focus after fluid percussion injury in the rat. J Neurophysiol. (2010) 104:3345–60. 10.1152/jn.00398.2010 20861444 PMC3007644

[386] Kinboshi M Mukai T Nagao Y Matsuba Y Tsuji Y Adamo MCD. Inhibition of inwardly rectifying potassium (Kir) 4.1 channels facilitates brain-derived neurotrophic factor (BDNF) expression in astrocytes. Front Mol Neurosci. (2017) 10:408. 10.3389/fnmol.2017.00408 29358904 PMC5768989

[387] Murray KD Isackson PJ Eskin TA King MA Montesinos SP Abraham LA. Altered mRNA expression for brain- derived neurotrophic factor and type II calcium/calmodulin-dependent protein kinase in the hippocampus of patients with intractable temporal lobe epilepsy. J Comp Neurol. (2000) 422:411–22. 10.1002/(sici)1096-9861(20000320)418:4<411::aid-cne4>3.0.co;2-f 10713570

[388] Heinrich C Lähteinen S Suzuki F Anne-Marie L Huber S Häussler U. Neurobiology of disease increase in BDNF-mediated TrkB signaling promotes epileptogenesis in a mouse model of mesial temporal lobe epilepsy. Neurobiol Dis. (2011) 42:35–47. 10.1016/j.nbd.2011.01.001 21220014

[389] Tanaka T Saito H Matsuki N. Inhibition of GABA A synaptic responses by brain-derived neurotrophic factor (BDNF) in rat hippocampus. J Neurosci. (1997) 17:2959–66. 10.1523/JNEUROSCI.17-09-02959.1997 9096132 PMC6573653

[390] Rivera C Li H Thomas-Crusells J Lahtinen H Viitanen T Nanobashvili A. BDNF-induced TrkB activation down-regulates the K^+^-Cl^−^ cotransporter KCC2 and impairs neuronal Cl– extrusion. J Cell Biol. (2002) 159:747–52. 10.1083/jcb.200209011 12473684 PMC2173387

[391] Kahle KT Deeb TZ Puskarjov M Silayeva L Liang B. Modulation of neuronal activity by phosphorylation of the K-Cl cotransporter KCC2. Trends Neurosci. (2015) 36:726–37. 10.1016/j.tins.2013.08.006 24139641 PMC4381966

[392] Woo N Lu J England R Mcclellan R Dufour S Mount DB. Hyperexcitability and epilepsy associated with disruption of the mouse neuronal-specific K–Cl cotransporter gene. Hippocampus. (2002) 268:258–68. 10.1002/hipo.10014 12000122

[393] Girouard H Bonev AD Hannah RM Meredith A Aldrich RW Nelson MT. Astrocytic endfoot Ca^2+^and BK channels determine both arteriolar dilation and constriction. Proc Natl Acad Sci USA. (2009) 107:1–6. 10.1073/pnas.0914722107 20133576 PMC2840528

[394] N'Gouemo P. Targeting BK (big potassium) channels in epilepsy. Expert Opin Ther Targets. (2012) 15:1283–95. 10.1517/14728222.2011.620607 21923633 PMC3219529

[395] Brenner R Chen QH Vilaythong A Toney GM Noebels JL Aldrich RW. BK channel β4 subunit reduces dentate gyrus excitability and protects against temporal lobe seizures. Nat Neurosci. (2005) 8:1752–9. 10.1038/nn1573 16261134

[396] Shruti S Clem RL Barth AL. A seizure-induced gain-of-function in BK channels is associated with elevated firing activity in neocortical pyramidal neurons. Neurobiol Dis. (2008) 30:323–30. 10.1016/j.nbd.2008.02.002 18387812 PMC2665726

[397] Liu X Tao J Zhang S Lan W Wang C Ji Y. Selective blockade of neuronal BK (α ^+^ β4) channels preventing epileptic seizure. J Med Chem. (2020) 63:216–30. 10.1021/acs.jmedchem.9b01241 31838846

[398] Whitmire LE Ling L Bugay V Carver CM Timilsina S Chuang HH. Downregulation of KCNMB4 expression and changes in BK channel subtype in hippocampal granule neurons following seizure activity. PLoS ONE. (2017) 12:e0188064. 10.1371/journal.pone.0188064 29145442 PMC5690595

[399] Mehranfard N Gholamipour-Badie H Motamedi F Janahmadi M Naderi N. Long-term increases in BK potassium channel underlie increased action potential firing in dentate granule neurons following pilocarpine-induced status epilepticus in rats. Neurosci Lett. (2015) 585:88–91. 10.1016/j.neulet.2014.11.041 25434869

[400] Shirazi-Zand Z Ahmad-Molaei L Motamedi F Naderi N. The role of potassium BK channels in anticonvulsant effect of cannabidiol in pentylenetetrazole and maximal electroshock models of seizure in mice. Epilepsy Behav. (2013) 28:1–7. 10.1016/j.yebeh.2013.03.009 23644464

[401] Wickenden AD. Potassium channels as anti-epileptic drug targets. Neuropharmacology. (2002) 43:1055–60. 10.1016/S0028-3908(02)00237-X 12504910

[402] Dvorzhak A Vagner T Kirmse K Grantyn R. Functional indicators of glutamate transport in single striatal astrocytes and the influence of Kir4. 1 in normal and huntington mice. J Neurosci. (2016) 36:4959–75. 10.1523/JNEUROSCI.0316-16.2016 27147650 PMC6601850

[403] Pappalardo LW Samad OA Black JA Waxman SG. Voltage-gated sodium channel Nav 1.5 contributes to astrogliosis in an *in vitro* model of glial injury via reverse Na^+^/Ca^2+^ exchange. Glia. (2015) 62:1162–75. 10.1002/glia.22671 24740847 PMC4060891

[404] Black JA Newcombe J Waxman SG. Astrocytes within multiple sclerosis lesions upregulate sodium channel Nav1.5. Brain. (2010) 133:835–46. 10.1093/brain/awq003 20147455

[405] Pappalardo LW Shujun L Black JA Waxman SG. Dynamics of sodium channel Nav 1.5 expression in astrocytes in mouse models of multiple sclerosis. Neuroreport. (2014) 25:1208–15. 10.1097/WNR.0000000000000249 25144393 PMC4159404

[406] Pappalardo LW Black JA Waxman SG Haven N Haven W. Sodium channels in astroglia and microglia. Glia. (2016) 64:1628–45. 10.1002/glia.22967 26919466 PMC5730353

[407] Vaillend C Mason SE Cuttle MF Alger BE. Mechanisms of neuronal hyperexcitability caused by partial inhibition of Na^+^-K^+^-ATPases in the rat CA1 hippocampal region. J Neurophysiol. (2002) 88:2963–78. 10.1152/jn.00244.2002 12466422

[408] Clapcote SJ Duffy S Xie G Kirshenbaum G Bechard AR Schack VR. Mutation I810N in the α3 isoform of Na^+^, K^+^-ATPase causes impairments in the sodium pump and hyperexcitability in the CNS. Proc Natl Acad Sci USA. (2009) 106:14085–90. 10.1073/pnas.0904817106 19666602 PMC2729024

[409] Gallanti A Tonelli A Cardin V Bussone G Bresolin N Bassi MT. A novel *de novo* nonsense mutation in ATP1A2 associated with sporadic hemiplegic migraine and epileptic seizures. J Neurol Sci. (2008) 273:123–6. 10.1016/j.jns.2008.06.006 18644608

[410] Su GUI Haworth RA Dempsey RJ Sun D Haworth RA Dempsey RJ. Regulation of Na^+^-K^+^-Cl^−^ cotransporter in primary astrocytes by dibutyryl cAMP and high [K^+^] o. Am J Physiol Cell Physiol. (2000) 297:1710–21. 10.1152/ajpcell.2000.279.6.C1710 11078685

[411] Su GUI Kintner DB Flagella M Shull GE Sun D Kintner DB. Astrocytes from Na^+^ -K^+^ -Cl - cotransporter-null mice exhibit absence of swelling and decrease in EAA release. Am J Physiol Cell Physiol. (2001) 53792:1147–60. 10.1152/ajpcell.00538.2001 11940530

[412] Østby I Øyehaug L Einevoll GT Nagelhus EA Plahte E Zeuthen T. Astrocytic mechanisms explaining neural-activity-induced shrinkage of extraneuronal space. PLoS Comput Biol. (2009) 5:e1000272. 10.1371/journal.pcbi.1000272 19165313 PMC2613522

[413] Malarkey EB Ni Y Parpura V. Ca^2+^ entry through TRPC1 channels contributes to intracellular Ca^2+^ dynamics and consequent glutamate release from rat astrocytes. Glia. (2008) 835:821–35. 10.1002/glia.20656 18338793

[414] Verkhratsky A Reyes RC Parpura V. TRP channels coordinate ion signalling in astroglia. Rev Physiol Biochem Pharmacol. (2013) 166:1–16. 10.1007/112 23784619 PMC4331456

[415] Anderson CM Swanson RA Astrocyte glutamate transport : review of properties, regulation, and physiological functions. Glia. (2000) 14:1–14. 10.1002/1098-1136(200010)32:1<1::AID-GLIA10>3.0.CO;2-W 10975906

[416] Uwechue NM Marx M Chevy Q Billups B. Activation of glutamate transport evokes rapid glutamine release from perisynaptic astrocytes. J Physiol. (2012) 10:2317–31. 10.1113/jphysiol.2011.226605 22411007 PMC3424755

[417] Kleene R Loers G Langer J Frobert Y Buck F Schachner M. Prion protein regulates glutamate-dependent lactate transport of astrocytes. J Neurosci. (2007) 27:12331–40. 10.1523/JNEUROSCI.1358-07.2007 17989297 PMC6673274

[418] Minelli A Castaldo P Gobbi P Salucci S Magi S Amoroso S. Cellular and subcellular localization of Na^+^- Ca^2+^ exchanger protein isoforms, NCX1, NCX2, and NCX3 in cerebral cortex and hippocampus of adult rat. Cell Calcium. (2007) 41:221–34. 10.1016/j.ceca.2006.06.004 16914199

[419] Shigetomi E Tong X Kwan KY Corey DP Baljit S. TRPA1 channels regulate astrocyte resting calcium and inhibitory synapse efficacy through GAT-3. Nat Neurosci. (2012) 15:70–80. 10.1038/nn.3000.TRPA1 22158513 PMC3282183

[420] Shigetomi E Jackson-Weaver O Huckstepp RT O'Dell TJ Khakh BS. TRPA1 channels are regulators of astrocyte basal calcium levels and long-term potentiation via constitutive d-serine release. J Neurosci. (2013) 33:10143–53. 10.1523/JNEUROSCI.5779-12.2013 23761909 PMC3682388

[421] Lee M Ting KK Adams S Brew BJ Chung R Guillemin GJ. Characterisation of the expression of NMDA receptors in human astrocytes. PLoS ONE. (2010) 5:e14123. 10.1371/journal.pone.0014123 21152063 PMC2994931

[422] Palygin O Lalo U Verkhratsky A Pankratov Y. Ionotropic NMDA and P2X 1/5 receptors mediate synaptically induced Ca 2 ^+^ signalling in cortical astrocytes. Cell Calcium. (2010) 48:225–31. 10.1016/j.ceca.2010.09.004 20926134

[423] Mikawa S Wang C Shu F Wang T Fukuda A Sato K. Developmental changes in KCC1, KCC2 and NKCC1 mRNAs in the rat cerebellum. Dev Brain Res. (2002) 136:93–100. 10.1016/s0165-3806(02)00345-0 12101026

[424] Gagnon KBE Adragna NC Fyffe REW Lauf PK. Characterization of glial cell K-Cl cotransport. Cell Physiol Biochem. (2007) 20:121–30. 10.1159/000104160 17595522

[425] Ringel F Plesnila N. Expression and functional role of potassium-chloride cotransporters (KCC) in astrocytes and C6 glioma cells. Neurosci Lett. (2008) 442:219–23. 10.1016/j.neulet.2008.07.017 18638521

[426] Kaila K Price TJ Payne JA Puskarjov M Voipio J. Cation-chloride cotransporters in neuronal development, plasticity and disease. Nat Rev Neurosci. (2015) 15:637–54. 10.1038/nrn3819 25234263 PMC4294553

[427] Mu A Pablo M Defelipe J Alvarez-Leefmans FJ. Cation-chloride cotransporters and GABA-ergic innervation in the human epileptic hippocampus. Epilepsia. (2007) 48:663–73. 10.1111/j.1528-1167.2007.00986.x 17319917

[428] Chen L Wan L Wu Z Ren W Yian H Qian B. KCC2 downregulation facilitates epileptic seizures. Sci Rep. (2017) 7:156. 10.1038/s41598-017-00196-7 28279020 PMC5427808

[429] Eftekhari S Habibabadi M Ziarani N Sohrab S. Bumetanide reduces seizure frequency in patients with temporal lobe *epilepsy*. Epilepsia. (2013) 54:10–13. 10.1111/j.1528-1167.2012.03654.x 23061490

[430] Larsen BR Assentoft M Cotrina ML Hua SZ Nedergaard M Kaila K. Contributions of the Na^+^/K^+^-ATPase, NKCC1, and Kir4.1 to hippocampal K^+^ clearance and volume responses. Glia. (2014) 62:608–22. 10.1002/glia.22629 24482245 PMC4302754

[431] Gharaylou Z Shafaghi L Oghabian MA Yoonessi A Tafakhori A Ananloo ES. Longitudinal effects of bumetanide on neuro-cognitive functioning in drug-resistant epilepsy. Front Neurol. (2019) 10:1–11. 10.3389/fneur.2019.00483 31133976 PMC6517515

[432] Kim SY Buckwalter M Soreq H Vezzani A Kaufer D. Blood–brain barrier dysfunction–induced inflammatory signaling in brain pathology and epileptogenesis. Epilepsia. (2012) 53:37–44. 10.1111/j.1528-1167.2012.03701.x 23134494 PMC3703535

[433] Abbott NJ Patabendige AAK Dolman DEM Yusof SR Begley DJ. Structure and function of the blood-brain barrier. Neurobiol Dis. (2010) 37:13–25. 10.1016/j.nbd.2009.07.030 19664713

[434] Sobue K Yamamoto N Yoneda K Hodgson ME Yamashiro K Tsuruoka N. Induction of blood-brain barrier properties in immortalized bovine brain endothelial cells by astrocytic factors. Neurosci Res. (1999) 35:155–64. 10.1016/S0168-0102(99)00079-6 10616919

[435] Régina A Morchoisne S Borson ND McCall AL Drewes LR Roux F. Factor(s) released by glucose-deprived astrocytes enhance glucose transporter expression and activity in rat brain endothelial cells. Biochim Biophys Acta Mol Cell Res. (2001) 1540:233–42. 10.1016/S0167-4889(01)00133-1 11583818

[436] Schinkel AH. P-Glycoprotein, a gatekeeper in the blood-brain barrier. Adv Drug Deliv Rev. (1999) 36:179–94. 10.1016/S0169-409X(98)00085-4 10837715

[437] Gaillard PJ Van Der Sandt ICJ Voorwinden LH Vu D Nielsen JL De Boer AG Astrocytes increase the functional expression of P-glycoprotein in an *in vitro* model of the blood-brain barrier. Pharm Res. (2000) 17:1198–205. 10.1023/A:1026406528530 11145224

[438] Dehouck M-P Méresse S Delorme P Fruchart J-C Cecchelli R An easier, reproducible, and mass-production method to study the blood–brain barrier *in vitro*. J Neurochem. (1990) 54:1798–801. 10.1111/j.1471-4159.1990.tb01236.x 2182777

[439] Haseloff RF Blasig IE Bauer HC Bauer H. In search of the astrocytic factor(s) modulating blood-brain barrier functions in brain capillary endothelial cells *in vitro*. Cell Mol Neurobiol. (2005) 25:25–39. 10.1007/s10571-004-1375-x 15962507 PMC11529558

[440] Abbott NJ. Dynamics of CNS Barriers: Evolution, Differentiation, and Modulation. Cell Mol Neurobiol. (2005) 25:5–23. 10.1007/s10571-004-1374-y 15962506 PMC11529509

[441] Alvarez JI Katayama T Prat A. *Glia*l influence on the blood brain barrier. Glia. (2013) 61:1939–58. 10.1002/glia.22575 24123158 PMC4068281

[442] Abbott NJ. Inflammatory mediators and modulation of blood-brain barrier permeability. Cell Mol Neurobiol. (2000) 20:131–47. 10.1023/A:1007074420772 10696506 PMC11537513

[443] Schwaninger M Sallmann S Petersen N Schneider A Prinz S Libermann TA. Bradykinin induces interleukin-6 expression in astrocytes through activation of nuclear factor-κB. J Neurochem. (1999) 73:1461–6. 10.1046/j.1471-4159.1999.0731461.x 10501190

[444] Smith NM Giacci MK Gough A Bailey C McGonigle T Black AMB. Inflammation and blood-brain barrier breach remote from the primary injury following neurotrauma. J Neuroinflamm. (2018) 15:1–18. 10.1186/s12974-018-1227-0 29981582 PMC6035802

[445] Vliet EA Van Arau SC Redeker S Schaik R Van Aronica E Gorter JA. Blood – brain barrier leakage may lead to progression of temporal lobe epilepsy. Brain. (2007) 130:521–34. 10.1093/brain/awl318 17124188

[446] Tomkins O Feintuch A Benifla M Cohen A Friedman A Shelef I. Blood-brain barrier breakdown following traumatic brain injury : a possible role in posttraumatic epilepsy. Cardiovasc Psychiatry Neurol. (2011) 2011:1–11. 10.1155/2011/765923 21436875 PMC3056210

[447] Marchi N Angelov L Masaryk T Fazio V Granata T Hernandez N. Seizure-promoting effect of blood–brain barrier *disruption*. Epilepsia. (2014) 48:732–42. 10.1111/j.1528-1167.2007.00988.x 17319915 PMC4135474

[448] van Vliet EA Aronica E Gorter JA. Role of blood-brain barrier in temporal lobe epilepsy and pharmacoresistance. Neuroscience. (2014) 277:455–73. 10.1016/j.neuroscience.2014.07.030 25080160

[449] Rigau V Morin M Rousset M-C de Bock F Lebrun A Coubes P. Angiogenesis is associated with blood-brain barrier permeability in temporal lobe epilepsy. Brain. (2007) 130:1942–56. 10.1093/brain/awm118 17533168

[450] Lebrun A Rousset M Fagni L Bock D Lerner-Natoli M. Epileptiform activity induces vascular remodeling and zonula occludens 1 downregulation in organotypic hippocampal cultures : role of VEGF signaling pathways me. J Neurosci. (2011) 31:10677–88. 10.1523/JNEUROSCI.5692-10.2011 21775611 PMC6622643

[451] Castañeda-Cabral JL Beas-Zárate C Rocha-Arrieta LL Orozco-Suárez SA Alonso-Vanegas M Guevara-Guzmán R. Increased protein expression of VEGF-A, VEGF-B, VEGF-C and their receptors in the temporal neocortex of pharmacoresistant temporal lobe epilepsy patients. J Neuroimmunol. (2019) 328:68–72. 10.1016/j.jneuroim.2018.12.007 30597392

[452] Montpellier D Cerebrovascular remodeling and epilepsy. Neuroscientist. (2013) 19:304–12. 10.1177/1073858412462747 23072899 PMC3701121

[453] Ivens S Kaufer D Flores LP Bechmann I Zumsteg D Tomkins O. TGF- b receptor-mediated albumin uptake into astrocytes is involved in neocortical epileptogenesis. Brain. (2007) 130:535–47. 10.1093/brain/awl317 17121744

[454] Aronica E Vliet EA Van Mayboroda OA Troost D Lopes FH Gorter JA. Upregulation of metabotropic glutamate receptor subtype mGluR3 and mGluR5 in reactive astrocytes in a rat model of mesial temporal lobe epilepsy. Eur J Neurosci. (2000) 12:2333–44. 10.1046/j.1460-9568.2000.00131.x 10947812

[455] Perillan PR Chen M Potts EA Simard JM Given DM. Transforming growth factor-beta 1 regulates Kir2.3 inward rectifier K^+^ channels via phospholipase C and protein kinase c-delta in reactive astrocytes from adult rat brain. J Biol Chem. (2002) 277:1974–80. 10.1074/jbc.M107984200 11713246

[456] Braganza O Bedner P Hüttmann K Staden von E Friedman A Seifert G. Albumin is taken up by hippocampal NG2 cells and astrocytes and decreases gap junction coupling. Epilepsia. (2013) 53:1898–906. 10.1111/j.1528-1167.2012.03665.x 22967085 PMC3651829

[457] Seiffert E Dreier JP Ivens S Bechmann I Tomkins O Heinemann U. Lasting blood – brain barrier disruption induces epileptic focus in the rat somatosensory cortex. J Neurosci. (2004) 24:7829–36. 10.1523/JNEUROSCI.1751-04.2004 15356194 PMC6729929

[458] Levy N Milikovsky DZ Baranauskas G Vinogradov E David Y Ketzef M. Differential TGF- β signaling in glial subsets underlies IL-6 – mediated epileptogenesis in mice. J Immunol. (2015) 195:1713–22. 10.4049/jimmunol.1401446 26136430

[459] Kimple AJ Yasgar A Hughes M Jadhav A Willard FS Robin E. A high-throughput fluorescence polarization assay for inhibitors of the goloco motif/G-alpha interaction. Comb Chem High Throughput Screen. (2009) 11:396–409. 10.2174/138620708784534770 18537560 PMC2440576

[460] Weissberg I Wood L Kamintsky L Vazquez O Milikovsky DZ Alexander A Neurobiology of disease albumin induces excitatory synaptogenesis through astrocytic TGF- β/ALK5 signaling in a model of acquired epilepsy following blood – brain barrier dysfunction. Neurobiol Dis. (2015) 78:115–25. 10.1016/j.nbd.2015.02.029 25836421 PMC4426044

[461] Vliet EA Van Zibell G Pekcec A Schlichtiger J Edelbroek PM Holtman L. COX-2 inhibition controls P-glycoprotein expression and promotes brain delivery of phenytoin in chronic epileptic rats. Neuropharmacology. (2010) 58:404–12. 10.1016/j.neuropharm.2009.09.012 19786037

[462] Dombrowski SM Desai SY Marroni M Cucullo L Goodrich K Bingaman W. Overexpression of multiple drug resistance genes in endothelial cells from patients with refractory Epilepsy. (2001) 42:1501–6. 10.1046/j.1528-1157.2001.12301.x 11879359

[463] Löscher W Potschka H. Role of multidrug transporters in pharmacoresistance to antiepileptic drugs. J Pharmacol Exp Ther. (2002) 301:7–14. 10.1124/jpet.301.1.7 11907151

[464] Sisodiya SM Lin W Harding BN Squier MV Thom M. Drug resistance in epilepsy: expression of drug resistance proteins in common causes of refractory epilepsy. Brain. (2002) 125:22–31. 10.1093/brain/awf002 11834590

[465] Aronica E Sisodiya SM Gorter JA. Cerebral expression of drug transporters in epilepsy. Adv Drug Deliv Rev. (2012) 64:919–29. 10.1016/j.addr.2011.11.008 22138133

[466] Schinkel AH Jonker JW. Mammalian drug efflux transporters of the ATP binding cassette (ABC) family: an overview. Adv Drug Deliv Rev. (2003) 55:3–29. 10.1016/s0169-409x(02)00169-2 12535572

[467] Sun H Dai H Shaik N Elmquist WF. Drug efflux transporters in the CNS. Adv Drug Deliv Rev. (2003) 55:83–105. 10.1016/s0169-409x(02)00172-2 12535575

[468] Löscher W. Drug transporters in the epileptic brain. Epilepsia. (2007) 48:8–13. 10.1111/j.1528-1167.2007.00993.x 17316407

[469] Brandt C Bethmann K Gastens AM Löscher W. The multidrug transporter hypothesis of drug resistance in epilepsy: proof-of-principle in a rat model of temporal lobe epilepsy. Neurobiol Dis. (2006) 24:202–11. 10.1016/j.nbd.2006.06.014 16928449

[470] Van Vliet EA Van Schaik R Edelbroek PM Redeker S Aronica E Wadman WJ. Inhibition of the multidrug transporter P-glycoprotein improves seizure control in phenytoin-treated chronic epileptic rats. Epilepsia. (2006) 47:672–80. 10.1111/j.1528-1167.2006.00496.x 16650133

[471] Vliet EA Van Schaik R Van Edelbroek PM Voskuyl RA Redeker S Aronica E. Region-specific overexpression of P-glycoprotein at the blood-brain barrier affects brain uptake of phenytoin in epileptic *rats*. J Pharmacol Exp Ther. (2007) 322:141–7. 10.1124/jpet.107.121178 17392402

[472] Lange ECM De Berg DJ Bellanti F Voskuyl RA Syvänen S. P-glycoprotein protein expression versus functionality at the blood-brain barrier using immunohistochemistry, microdialysis and mathematical modeling. Eur J Pharm Sci. (2018) 124:61–70. 10.1016/j.ejps.2018.08.022 30144528

[473] Borlot F Wither RG Ali A Wu N Verocai F Andrade DM. A pilot double-blind trial using verapamil as adjuvant therapy for refractory seizures. Epilepsy Res. (2014) 108:1642–51. 10.1016/j.eplepsyres.2014.08.009 25223728

[474] Nicita F Spalice A Papetti L Nikanorova M Iannetti P Parisi P. Efficacy of verapamil as an adjunctive treatment in children with drug-resistant epilepsy : a pilot study. Seizure Eur J Epilepsy. (2014) 23:36–40. 10.1016/j.seizure.2013.09.009 24113539

[475] Summers MA Moore JL Mcauley JW. Use of verapamil as a potential P-glycoprotein inhibitor in a patient with refractory epilepsy. Ann Pharmacother. (2004) 38:1631–4. 10.1345/aph.1E068 15328394

[476] Pirker S Baumgartner C. Termination of refractory focal status epilepticus by the P-glycoprotein inhibitor verapamil. Eur J Neurol. (2011) 18:e151. 10.1111/j.1468-1331.2011.03513.x 22097953

[477] Asadi-Pooya AA Ali SM Abdi-Ardekani A Sperling MR. Epilepsy and behavior adjunctive use of verapamil in patients with refractory temporal lobe epilepsy : a pilot study. Epilepsy Behav. (2013) 29:150–4. 10.1016/j.yebeh.2013.07.006 23973639

[478] Narayanan J Frech R Walters S Patel V Frigerio R Maraganore DM. Low dose verapamil as an adjunct therapy for medically refractory epilepsy – an open label pilot study. Epilepsy Res. (2016) 126:197–200. 10.1016/j.eplepsyres.2016.07.004 27513375

[479] Metea MR Newman EA. Glial cells dilate and constrict blood vessels: a mechanism of neurovascular coupling. J Neurosci. (2006) 26:2862–70. 10.1523/JNEUROSCI.4048-05.2006 16540563 PMC2270788

[480] Fabene PF Marzola P Sbarbati A Bentivoglio M. Magnetic resonance imaging of changes elicited by status epilepticus in the rat brain : diffusion-weighted and T2-weighted images, regional blood volume maps, and direct correlation with tissue and cell damage. Neuroimage. (2003) 18:375–89. 10.1016/s1053-8119(02)00025-3 12595191

[481] Winkler MKL Chassidim Y Lublinsky S Revankar GS Major S Kang E Impaired neurovascular coupling to ictal epileptic activity and spreading depolarization in a patient with subarachnoid hemorrhage: possible link to blood–brain barrier dysfunction. Epilepsia. (2013) 53:22–30. 10.1111/j.1528-1167.2012.03699.x PMC3625730 23134492

[482] Zhao M Suh M Ma H Perry C Geneslaw A Schwartz TH. Focal increases in perfusion and decreases in hemoglobin oxygenation precede seizure onset in spontaneous human epilepsy. Epilepsia. (2007) 48:2059–67. 10.1111/j.1528-1167.2007.01229.x 17666071

[483] Gómez-Gonzalo M Losi G Brondi M Uva L Sato SS Mansvelder HD Ictal but not interictal epileptic discharges activate astrocyte endfeet and elicit cerebral arteriole responses. Front Cell Neurosci. (2011) 5:8 10.3389/fncel.2011.00008 21747758 PMC3128928

[484] Vezzani A Pascente R Ravizza T. Biomarkers of epileptogenesis: the focus on glia and cognitive dysfunctions. Neurochem Res. (2017) 42:2089–98. 10.1007/s11064-017-2271-3 28434163

[485] van Vliet EA Aronica E Vezzani A Ravizza T. Review: neuroinflammatory pathways as treatment targets and biomarker candidates in epilepsy: emerging evidence from preclinical and clinical studies. Neuropathol Appl Neurobiol. (2018) 44:91–111. 10.1111/nan.12444 28977690

